# Mathematical modeling of the combined effects of thermal burn and local irradiation

**DOI:** 10.1371/journal.pone.0341595

**Published:** 2026-02-10

**Authors:** Quintessa Hay, Rachel Jennings, Amy Creel, Kyle Gaffney, Christina Wagner, Kidist Maxwell, Rafael Henriquez-Rivera, Ginu Unnikrishnan, Tyler Dant

**Affiliations:** 1 Algorithms, Modeling and Assessments Division, Applied Research Associates, Inc., Arlington, Virginia, United States of America; 2 Takeda Pharmaceuticals, Cambridge, Massachusetts, United States of America; 3 Rocky Mountain Division, Applied Research Associates, Inc., Littleton, Colorado, United States of America; 4 Software Enterprise Division; Applied Research Associates, Inc., Raleigh, North Carolina, United States of America; Universidad Especializada de las Americas, PANAMA

## Abstract

Radiation combined injury (RCI), resulting from ionizing radiation exposure accompanied by other injuries such as burn, laceration, or fracture, is associated with higher rates of mortality and severe effects. Current available models lack the ability to capture synergistic effects associated with RCI and/or rely on data-driven approaches that are limited in their predictive capabilities. To address this, we developed a mechanistic mathematical model for local radiation exposure combined with burn injury that captures the inflammatory response and early fibroblast activity associated with injury resolution. We utilized sensitivity analysis and parameter sampling methods to leverage limited data. The model was able to reproduce inflammatory and fibroblast behavior consistent with observed thermal injury and combined injury profiles. The formulation of a mechanistic model for combined injury adds increased modeling flexibility allowing for further exploration of the underlying inflammatory mechanisms and provides a framework for leveraging minimal data for improved predictive models of RCI.

## Introduction

In the context of nuclear accidents, such as Chernobyl, radiation combined injury (RCI) describes a condition wherein radiation exposure is combined with additional injuries such as burns, wounds, and other trauma [1–3] Combined injuries are associated with greater tissue damage and an increased risk of adverse outcomes, such as lethality [4,5], secondary infection [6], and sepsis [7–9]. Moreover, synergistic effects have also been observed [4,9] where the combined effects are more severe than the sum of the individual injuries.

The pathophysiology of individual burn injuries has been adequately captured using mathematical models [10–12], which determine the resulting tissue damage and underlying cellular activity during injury resolution. Similarly, mathematical models of injury from ionizing radiation have also been developed [13–18]. These models typically address radiation at the cellular level and provide estimations for direct cell damage, myelopoietic recovery, and survival. However, a combined injury model that captures the synergistic pathophysiologic effects between a thermal burn combined with radiation is not currently available. This is especially crucial since local ionizing radiation exposure has been observed to delay the burn injury wound healing process [19–22] resulting in depleted circulating immune cells and additional damage to resident cell populations. Local ionizing radiation exposure has also been evidenced to damage dermal fibroblasts [23] and microvasculature [24], which provide additional causes of delay and dysregulation of the inflammatory response [25,26]. These effects of both the burn injury and local radiation exposure contribute synergistically to delayed resolution of injury and inflammatory imbalances.

Given the lack of mechanistic mathematical models, empirical models have been primarily used to estimate injury prevalence [27] and probability of survival [28]. However, empirical models are bound by their data and rarely maintain accuracy when extrapolated beyond recorded time points. Alternatively, mechanistic models can capture system dynamics that allow for exploration past the constraints of available data as well as the capability to estimate related effects such as medical outcomes associated with the injury.

For our model, we developed a system of ordinary differential equations (ODEs) which capture the immune response to radiation combined injury with burn. A variety of methods have been used to mechanistically model the immune response to injury including systems of ODEs [29–32], partial differential equations (PDEs) [33,34], and agent-based models [35–37]. Each method has advantages and disadvantages, mainly regarding biological complexity and computation time. PDEs and agent-based models, for example, are often chosen due to their ability to incorporate spatial gradients important in immune cell infiltration at the wound site. However, this added complexity can often result in increased computation time. For this reason, we have selected a system of ODEs that can sufficiently capture cellular population dynamics within the injury site and surrounding tissues while keeping computation time reasonable. This supports future integration into predictive software for modeling combined injury in nuclear incidents.

To model the effects of thermal burn combined with local radiation, we developed a time-dependent physiological model (TDPM), which includes immune cell infiltration, inflammation, and early fibroblast activity. We employed a combination of data, medical trends, and sensitivity analysis to generate plausible parameter sets and conducted numerical simulations to demonstrate the ability of the model to reproduce observed combined injury dynamics.

## Methods

The mathematical model is a system of ODEs representing the various immune cell populations, damaged tissue and cellular debris, and foreign pathogen. A system of ODEs was chosen to capture nonlinearities and feedback loops present in inflammatory signaling and immune cell response while keeping computation time reasonable. ODEs, however, cannot account for spatial complexity and assume the elements in the environment are well-mixed and evenly distributed throughout the space. One way to resolve this is to use a compartmental model in which cells and cellular mediators can travel between compartments. This method is employed in the present model.

**Fig 1:**
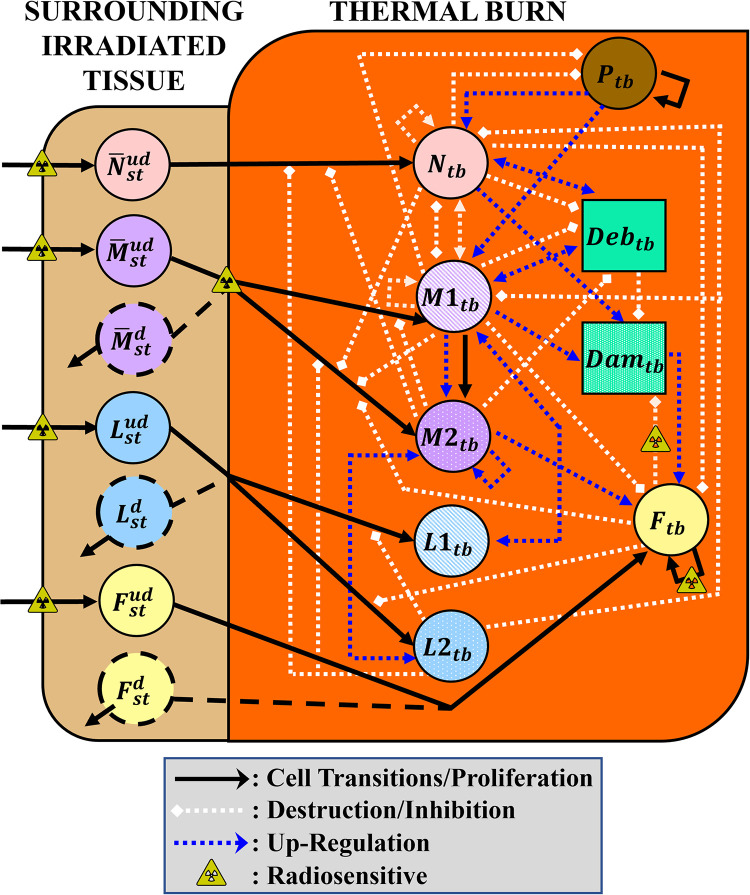
Schematic diagram of locally irradiated superficial thermal burn, the state variables, and their relationships. The model consists of the thermal burn compartment where cell populations experience damage from thermal fluence and the surrounding tissue compartment where resident immune cell populations experience damage from a prompt radiation dose. The variables model the inflammatory and early proliferation phases of wound healing. State variables are represented using circles and squares. The solid black lines indicate transitions between different cellular states. Solid lines that feedback into the state variable indicate proliferation. Initiated cellular interactions are captured by dotted lines and remain locally concentrated. Blue and white colors are used to distinguish between upregulation (i.e., increasing in response to the cells themselves) and destruction/inhibition, respectively. The toxic triangles indicate cell transitions and interactions affected by ionizing radiation that have been incorporated into the model.

In the mathematical model for a locally irradiated thermal burn injury, we consider two compartments: 1) the thermal burn compartment with activated immune cells, fibroblasts, and foreign pathogen, and 2) a surrounding irradiated tissue compartment, which serves as an intermediate compartment between circulating immune cells in the bloodstream and their activation at the injury site. Cell populations and their associated cytokine and chemokine biomolecular signals are represented with compartmental ODEs. A model schematic of the variables and interactions can be viewed in Fig 1. Detailed descriptions of model variable descriptions and symbols are available in Table 1. Parameter descriptions along with numerical values used in model simulations are provided in S1 Table.

**Table 1:** State variables with descriptions.

SYMBOL	DESCRIPTION
t	Time (in hours)
$D_{R}$	Radiation absorbed dose (in Gray)
*Surrounding Irradiated Tissue*	
${\overset{―}{N}}_{st}^{ud}$	Resting neutrophils in the surrounding tissue
${\overset{―}{M}}_{st}^{ud}$	Undamaged resident monocytes/macrophages in the surrounding tissue
${\overset{―}{M}}_{st}^{d}$	Damaged resident monocytes/macrophages in the surrounding tissue
$F_{st}^{ud}$	Undamaged resident fibroblasts in the surrounding tissue
$F_{st}^{d}$	Damaged resident fibroblasts in the surrounding tissue
$L_{st}^{ud}$	Undamaged resident T lymphocytes in the surrounding tissue
$L_{st}^{d}$	Damaged resident T lymphocytes in the surrounding tissue
*Local Thermal Burn*	
${D𝐚𝐦}_{tb}$	Damaged tissue (unitless)
${Deb}_{tb}$	Debris (unitless)
$N_{tb}$	Activated neutrophils at the wound site
${M1}_{tb}$	Classically activated macrophages (M1) at the wound site
${M2}_{tb}$	Alternatively activated macrophages (M2) at the wound site
$F_{tb}$	Fibroblasts at the wound site
${L1}_{tb}$	Pro-inflammatory T lymphocytes, including γδ-T cells, TH1 cells, and TH17 cells, at the wound site
${L2}_{tb}$	Anti-inflammatory T lymphocytes, including TH2 cells and regulatory T cells, at the wound site
$P_{tb}$	Pathogen levels at the wound site

The subscripts st and tb represent the surrounding tissue compartment and the thermal burn compartment, respectively. The superscripts ud and d represent undamaged and damaged cell populations, respectively. The damaged populations occur only in the surrounding tissue compartment as a result of DNA damage caused by radiation.

### Model equations

The system of ODEs were developed using terms and model structure similar to those found in previous models of inflammation [29–31] and proliferation [32] with modifications made to incorporate radiation effects, which are discussed herein. Additional details regarding the development of specific model terms can be found in S2 File. A few auxiliary functions (Equations 1–3) are used throughout the system of equations which represent an inhibitory function, $\Omega_{i}$, a hill function, $\Omega_{H}$, and a dose-dependent saturating function between 0 and 1, v, respectively. The inhibitory function and hill function are commonly used across models of inflammation [29–32].


$$\Omega_{i}\left(X;Y_{\text{1}},Y_{\text{1}}^{\infty },\ldots ,\;Y_{m},\;Y_{m}^{\infty }\right)=\frac{X}{\text{1}+{\left(\frac{Y_{\text{1}}}{Y_{\text{1}}^{\infty }}\right)}^{\text{2}}+\ldots +{\left(\frac{Y_{m}}{Y_{m}^{\infty }}\right)}^{\text{2}}}$$ (1)

$$\Omega_{H}\left(X,n,X_{H}\right)=\frac{X^{n}}{X_{H}^{n}+X^{n}}$$ (2)

$$v\left(t,D_{R};\gamma \right)=\left\{ \;\;\begin{array}{c} \text{1},\;\;{\;\;\;\;\;\;\;\;\;\;\;\;\;D}_{R}=\text{0}\\ \text{1}-\text{exp}\left(-\frac{\gamma }{D_{R}}t\right),\;\;D_{R}>\text{0} \end{array}\right.$$ (3)

### Damaged tissue and debris

Damaged tissue and cellular debris result from direct damage to cells from thermal fluence as well as collateral damage due to proteases and other tissue degrading molecules produced by neutrophils and M1 macrophages during the inflammatory phase [38–40]. The resulting cellular debris is then removed through phagocytosis by activated inflammatory cells (neutrophils and macrophages). Damaged tissue is then resolved through fibroblast proliferation which serves as the first step towards replacement of damaged tissue.


$$\begin{array}{c} \begin{array}{c} \frac{{\text{\textbackslash{}textit\{dDam}}_{tb}}{\}}\text{\textbackslash{}textit\{dt}\}=\underset{\begin{array}{c} \text{Collateral}\;\text{damage}\;\text{from}\\ \text{activated}\;\text{neutrophils} \end{array}}{\underbrace{k_{dn}\Omega_{H}\left(\Omega_{i}\left(N_{tb};L{\text{2}}_{tb},L{\text{2}}_{\text{2}}^{\infty }\right),\text{6},N_{H}\right)}}+\underset{\begin{array}{c} \text{Collateral}\;\text{damage}\;\text{from}\\ \text{activated}\;\text{M1}\;\text{macrophages} \end{array}}{\underbrace{k_{dm\text{1}}\Omega_{H}\left(\Omega_{i}\left(M{\text{1}}_{tb};L{\text{2}}_{tb},L{\text{2}}_{\text{2}}^{\infty }\right),\text{6},M{\text{1}}_{H}\right)}}\\ -\underset{\begin{array}{c} \text{Background}\;\text{resolution}\;\text{of}\\ \text{damage} \end{array}}{\underbrace{\rho_{dam}\Omega_{i}\left(Dam_{tb};Deb_{tb},Deb_{dam}^{\infty }\right)}}\\ -\underset{\begin{array}{c} \text{Resolution}\;\text{of}\;\text{damage}\;\text{by}\;\text{proliferating}\;\text{fibroblasts}\\ \left(\text{inhibited}\;\text{by}\;\text{radiation}\right) \end{array}}{\underbrace{\Omega_{i}\left(F_{tb};F_{st}^{d},\omega_{dam}\right)k_{df}\Omega_{i}\left(Dam_{tb};Deb_{tb},Deb_{dam}^{\infty }\right)}} \end{array} \end{array}$$ (4)

$$\begin{array}{c} \begin{array}{c} {\frac{\text{\textbackslash{}textit\{\{d}Deb}{\}}}_{tb}\}\text{\textbackslash{}textit\{dt}\}=\underset{\begin{array}{c} \text{Debris}\;\text{generated}\;\text{by}\\ \text{activated}\;\text{neutrophils} \end{array}}{\underbrace{k_{dn}\Omega_{H}\left(\Omega_{i}\left(N_{tb};L{\text{2}}_{tb},L{\text{2}}_{\text{2}}^{\infty }\right),\text{6},N_{H}\right)}}+\underset{\begin{array}{c} \text{Debris}\;\text{generated}\;\text{by}\\ \text{activated}\;\text{M1}\;\text{macrophages} \end{array}}{\underbrace{k_{dm\text{1}}\Omega_{H}\left(\Omega_{i}\left({M\text{1}}_{tb};L{\text{2}}_{tb},L{\text{2}}_{\text{2}}^{\infty }\right),\text{6},{M\text{1}}_{H}\right)}}\\ -\underset{\begin{array}{c} \text{Phagocytosis}\;\text{of}\;\text{debris}\;\text{by}\\ \text{activated}\;\text{neutrophils} \end{array}}{\underbrace{k_{dnp}N_{tb}\Omega_{H}\left(Deb_{tb},\text{1},Deb_{H}\right)}}-\underset{\begin{array}{c} \text{Phagocytosis}\;\text{of}\;\text{debris}\;\text{by}\\ \text{activated}\;\text{M1}\;\text{macrophages} \end{array}}{\underbrace{k_{dm\text{1}p}\Omega_{i}\left(M{\text{1}}_{tb};N_{tb},N_{\text{1}}^{\infty }\right)\Omega_{H}\left(Deb_{tb},\text{1},Deb_{H}\right)}}\\ -\underset{\begin{array}{c} \text{Phagocytosis}\;\text{of}\;\text{debris}\;\text{by}\\ \text{activated}\;\text{M2}\;\text{macrophages} \end{array}}{\underbrace{k_{dm\text{2}p}\Omega_{i}\left(M{\text{2}}_{tb};N_{tb},N_{\text{1}}^{\infty }\right)\Omega_{H}\left(Deb_{tb},\text{1},Deb_{H}\right)}}-\underset{\begin{array}{c} \text{Background}\;\text{removal}\\ \text{and}\;\text{decay} \end{array}}{\underbrace{d_{deb}Deb_{tb}}} \end{array} \end{array}$$ (5)

Although collagen is not explicitly modeled, damage resolution by activated fibroblasts captures early repair processes indicated by collagen deposition at the wound site. Collagen has been observed to be a good marker of tissue damage resolution in other mathematical models of wound healing [32]. Radiation has been evidenced to inhibit the deposition of collagen by fibroblasts [22,41,42], which is included as an inhibitory function in the repair term for the damage equation. The population of damaged fibroblasts in the surrounding tissue ($F_{st}^{d}$) inhibits the removal of damage.

### Neutrophils

Neutrophils are typically the first inflammatory cells to arrive at the wound site and are activated and recruited by damage-associated molecular patterns and other immune cells [40]. Neutrophils then work to phagocytize any debris and foreign matter.


$$\begin{array}{c} \frac{d\overset{―}{N}{\;}_{st}^{ud}}{dt}=\underset{\begin{array}{c} \text{Neutrophil}\;\text{recruitment}\\ \text{from}\;\text{the}\;\text{bloodstream} \end{array}}{\underbrace{v\left(t,Dose_{rad};\gamma_{n}\right)s_{nr}Dam_{tb}}}-\underset{\begin{array}{c} \text{Neutrophil}\\ \text{activation} \end{array}}{\underbrace{R_{n}\overset{―}{N}{\;}_{st}^{ud}}}-\underset{\begin{array}{c} \text{Decay} \end{array}}{\underbrace{d_{nr}\overset{―}{N}{\;}_{st}^{ud}}} \end{array}$$ (6)

$$\begin{array}{c} \frac{dN_{tb}}{dt}=\underset{\begin{array}{c} \text{Neutrophil}\\ \text{activation}\\ \text{at}\;\text{the}\;\text{injury}\\ \text{site} \end{array}}{\underbrace{R_{n}\overset{―}{N}{\;}_{st}^{ud}}}-\underset{\begin{array}{c} \text{Phagocytosis}\;\text{of}\;\text{neutrophils}\;\text{by}\\ \text{M1}\;\text{macrophages} \end{array}}{\underbrace{k_{nm\text{1}p}N_{tb}{\mathrm{\backslash itOmega}}_{i}\left(M{\text{1}}_{tb};N_{tb},N_{\text{1}}^{\infty }\right)}}-\underset{\begin{array}{c} \text{Phagocytosis}\;\text{of}\;\text{neutrophils}\;\text{by}\\ \text{M2}\;\text{macrophages} \end{array}}{\underbrace{k_{nm\text{2}p}N_{tb}{\mathrm{\backslash itOmega}}_{i}\left(M{\text{2}}_{tb};N_{tb},N_{\text{1}}^{\infty }\right)}}-\underset{\begin{array}{c} \text{Decay} \end{array}}{\underbrace{d_{n}N_{tb}}} \end{array}$$ (7)

$$R_{n}=\Omega_{i}\underset{\begin{array}{c} \text{Activation}\;\text{of}\;\text{neutrophils}\;\text{by}\\ \text{debris}\;\text{signals},\;\text{pathogen},\\ \text{and}\;\text{other}\;\text{neutrophils} \end{array}}{\underbrace{(k_{nd}{Deb}_{tb}+k_{np}P_{tb}+k_{nn}N_{tb}}};\underset{\begin{array}{c} \text{Inhibition}\;\text{from}\;\text{M2}\\ \text{macrophages}\;\text{and}\\ \text{L2}\;\text{lymphocytes} \end{array}}{\underbrace{M{\text{2}}_{tb},{M\text{2}}^{\infty },{L\text{2}}_{tb},{L\text{2}}_{\text{1}}^{\infty }}})$$ (8)

Localized ionizing radiation exposure damages the microvasculature [22,26] resulting in delayed infiltration of recruited neutrophils and other immune cells [25]. Revascularization is not explicitly modeled, so this delay is represented by a dose-dependent inhibitory function that decays over time and reduces the baseline influx of neutrophils.

### Monocytes and macrophages

Monocytes and macrophages are also recruited to the injury site to remove any debris and regulate the inflammatory response [40]. In our model, we use the term monocyte to represent the inactive form and macrophage to represent the activated form. Monocytes in the surrounding tissue are present at rest and have two states: undamaged or damaged, denoted by a ud or d superscript, respectively. Macrophages in the injury site can be activated on a spectrum presenting a range of pro- and anti-inflammatory behavior [43], which are generally labeled as M1 classically-activated and M2 alternatively-activated respectively. Both are used in the model to represent these general phenotypes.


$$\begin{array}{c} \frac{d\overset{―}{M}{\;}_{st}^{ud}}{dt}=\underset{\begin{array}{c} \text{Macrophage}\;\text{recruitment}\\ \text{from}\;\text{the}\;\text{bloodstream}\\ \left(\text{inhibited}\;\text{by}\;\text{radiation}\right) \end{array}}{\underbrace{v\left(t,D_{R};\gamma_{m}\right)s_{mr}}}-\underset{\begin{array}{c} \text{Activation}\;\text{to}\;\text{the}\\ \text{M1}\;\text{phenotype} \end{array}}{\underbrace{R_{m\text{1}}\overset{―}{M}{\;}_{st}^{ud}}}-\underset{\begin{array}{c} \text{Activation}\;\text{to}\;\text{the}\;\text{M2}\;\text{phenotype}\\ \left(\text{inhibited}\;\text{by}\;\text{radiation}\right) \end{array}}{\underbrace{{\mathrm{\backslash itOmega}}_{i}\left(R_{m\text{2}};{\overset{―}{M}}_{st}^{d},\omega_{m\text{2}}\right)\overset{―}{M}{\;}_{st}^{ud}}}-\;\underset{\begin{array}{c} \text{Decay} \end{array}}{\underbrace{d_{mr}^{ud}\overset{―}{M}{\;}_{st}^{ud}}} \end{array}$$ (9)

$$\begin{array}{c} \frac{d\overset{―}{M}{\;}_{st}^{d}}{dt}=\underset{\begin{array}{c} \text{Activation}\;\text{to}\;\text{the}\\ \text{M1}\;\text{phenotype} \end{array}}{\underbrace{-R_{m\text{1}}\overset{―}{M}{\;}_{st}^{d}}}-\underset{\begin{array}{c} \text{Activation}\;\text{to}\;\text{the}\;\text{M2}\;\text{phenotype}\\ \left(\text{inhibited}\;\text{by}\;\text{radiation}\right) \end{array}}{\underbrace{{\mathrm{\backslash itOmega}}_{i}\left(R_{m\text{2}};\overset{―}{M}{\;}_{st}^{d},\omega_{m\text{2}}\right)\overset{―}{M}{\;}_{st}^{d}}}-\;\underset{\begin{array}{c} \text{Decay} \end{array}}{\underbrace{d_{mr}^{d}\overset{―}{M}{\;}_{st}^{d}}} \end{array}$$ (10)

$$\begin{array}{c} \frac{d{M\text{1}}_{tb}}{dt}=\underset{\begin{array}{c} \text{Macrophage}\;\text{activation}\\ \text{at}\;\text{the}\;\text{injury}\;\text{site} \end{array}}{\underbrace{R_{m\text{1}}\left(\overset{―}{M}{\;}_{st}^{ud}+\overset{―}{M}{\;}_{st}^{d}\right)}}\;-\;\underset{\begin{array}{c} \text{Transition}\;\text{of}\;\text{M1}\;\text{macrophages}\;\text{to}\\ \text{M2}\;\text{macrophages} \end{array}}{\underbrace{\theta_{m\text{1}m\text{2}}{\;[k}_{nm\text{1}p}N_{tb}\Omega_{i}\left(M{\text{1}}_{tb};N_{tb},N_{\text{1}}^{\infty }\right)]}}\;-\;\underset{\begin{array}{c} \text{Decay} \end{array}}{\underbrace{d_{m\text{1}}M{\text{1}}_{tb}}} \end{array}$$ (11)

$$\begin{array}{c} \frac{d{M\text{2}}_{tb}}{dt}=\underset{\begin{array}{c} \text{Macrophage}\;\text{activation}\;\text{at}\;\text{the}\;\text{injury}\\ \text{site}\;(\text{inhibited}\;\text{by}\;\text{radiation}) \end{array}}{\underbrace{{\mathrm{\backslash itOmega}}_{i}\left(R_{m\text{2}};\overset{―}{M}{\;}_{st}^{d},\omega_{m\text{2}}\right)\left(\overset{―}{M}{\;}_{st}^{ud}+\overset{―}{M}{\;}_{st}^{d}\right)}}+\underset{\begin{array}{c} \text{Transition}\;\text{of}\;\text{M1}\;\text{macrophages}\;\text{to}\\ \text{M2}\;\text{macrophages} \end{array}}{\underbrace{\theta_{m\text{1}m\text{2}}{\;[k}_{nm\text{1}p}N_{tb}{\mathrm{\backslash itOmega}}_{i}\left(M{\text{1}}_{tb};N_{tb},N_{\text{1}}^{\infty }\right)]}}\;\;\;\;-\;\underset{\begin{array}{c} \text{Decay} \end{array}}{\underbrace{d_{m\text{2}}M{\text{2}}_{tb}}} \end{array}$$ (12)

$$\begin{array}{c} R_{m\text{1}}={𝛺}_{i}\underset{\begin{array}{c} \text{Activation}\;\text{by}\;\text{debris},\;\text{pathogen},\;\text{neutrophils}\\ \text{and}\;\text{macrophages} \end{array}}{\underbrace{(k_{m\text{1}d}{Deb}_{tb}+k_{m\text{1}p}P_{tb}+k_{m\text{1}n}N_{tb}+k_{m\text{1}m\text{1}}M{\text{1}}_{tb}}}+\underset{\begin{array}{c} \text{Activation}\;\text{by}\\ \text{lymphocytes} \end{array}}{\underbrace{k_{m\text{1}l\text{1}}L{\text{1}}_{tb}}};\;\underset{\begin{array}{c} \text{Inhibition}\;\text{by}\;\text{M2}\;\text{macrophages}\\ \text{and}\;\text{L2}\;\text{lymphocytes} \end{array}}{\underbrace{M{\text{2}}_{tb},M{\text{2}}^{\infty },L{\text{2}}_{tb},{L\text{2}}_{\text{1}}^{\infty }}}\;\;\;\;\;\;\;\;\;\;\;\;\;) \end{array}$$ (13)

$$\begin{array}{c} R_{m\text{2}}=\Omega_{i}\;\;\;\;\;\;\;\;\underset{\begin{array}{c} \text{Activation}\;\text{by}\;\text{macrophages}\;\text{and}\;\text{lymphocytes} \end{array}}{\underbrace{{(k}_{m\text{2}m\text{1}}M{\text{1}}_{tb}+k_{m\text{2}m\text{2}}M{\text{2}}_{tb}+k_{m\text{2}l\text{2}}L{\text{2}}_{tb}}};\;\underset{\begin{array}{c} \text{Inhibition}\;\text{by}\;\text{neutrophils},\\ \text{macrophages},\;\text{and}\\ \text{fibroblasts} \end{array}}{\underbrace{N_{tb},N_{\text{1}}^{\infty },M{\text{1}}_{tb},\;{M\text{1}}_{\text{1}}^{\infty },F_{tb},F^{\infty }}}) \end{array}$$ (14)

Undamaged monocytes in the surrounding tissue are recruited from the blood stream at a baseline constant rate, represented by the parameter $s_{mr}$ in the first term of Equation 9. Similar to the equation defined for neutrophils in the surrounding tissue (Equation 6), this baseline rate is inhibited when radiation injury occurs in a dose-dependent manner.

**Fig 2:**
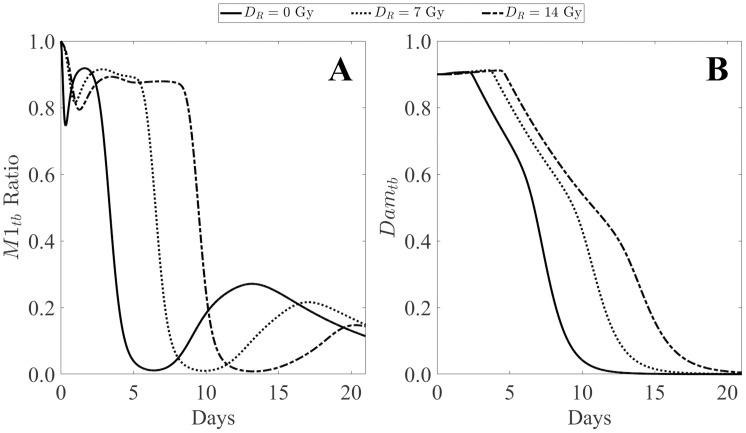
M1 macrophage ratio to total macrophages and damage variable with varying prompt radiation doses. The M1 ratio (panel A) denotes the total expression of M1 macrophages as a ratio of the total activated macrophage response such that 1 corresponds with 100% M1 activated macrophages and 0 corresponds with 0% M1 activated macrophages (100% M2 activated macrophages). Panel B shows the corresponding damage variable indicating damage resolution over time. Prolonged pro-inflammatory behavior has been widely evidenced to delay the wound healing process [48].

For prompt gamma radiation doses above 2 Gray (Gy), macrophages are polarized towards the classically activated M1 phenotype [44]. Given the prolific evidence of increased pro-inflammatory cytokines following irradiation [45–47], it is reasonable to assume that this overexpression of pro-inflammatory cytokines could be the mechanism by which activation towards the M1 phenotype occurs. For this reason, the alternative activation is dependent upon damaged macrophage cells in the surrounding tissue which includes all associated cytokines, chemokines, and other signals. This allows for both a time- and dose-dependent inhibition of the activation process toward the M2 phenotype. This can be observed in Fig 2. Panel A shows the scaled M1 ratio to total macrophages in the thermal burn compartment while panel B displays the corresponding damage variable over time. The plots exhibit the increased and prolonged activation toward the M1 phenotype with increasing radiation dose as well as the delayed wound healing exhibited by prolonged inflammation and delayed resolution of damage. This mechanism is represented in the first term of Equation 12. Consistent with current research [44], the activation towards the M2 phenotype is only inhibited for $D_{R}\geq \;\text{2}\;$ Gy, otherwise, activation occurs at the rate $R_{m\text{2}}$.

### T lymphocytes

T lymphocytes also infiltrate the injury during the inflammatory phase to aid in debris removal and inflammatory regulation, but typically at later time points than neutrophils and macrophages. Like monocytes, lymphocytes have inactive populations in the surrounding tissue and are highly radio sensitive [49,50]. Populations in the surrounding tissue will also be split between undamaged and damaged, denoted by a ud or d superscript, respectively. Only T-cell lymphocytes are considered in the present model which include the pro-inflammatory phenotypes (e.g., γδ T cells, TH1 cells, and T17 cells) and anti-inflammatory phenotypes (e.g., TH2 cells and regulatory T cells).


$$\begin{array}{c} \frac{dL_{st}^{ud}}{dt}=\underset{\begin{array}{c} \text{Lymphocyte}\;\text{recruitment}\\ \text{from}\;\text{the}\;\text{bloodstream}\\ \left(\text{inhibited}\;\text{by}\;\text{radiation}\right) \end{array}}{\underbrace{{v\left(t,Dose_{rad};\gamma_{l}\right)s}_{lr}}}\;-\;\underset{\begin{array}{c} \text{Activation}\;\text{to}\;\text{the}\\ \text{L1}\;\text{phenotype} \end{array}}{\underbrace{R_{l\text{1}}L_{st}^{ud}}}\;-\;\underset{\begin{array}{c} \text{Activation}\;\text{to}\;\text{the}\\ \text{L2}\;\text{phenotype} \end{array}}{\underbrace{R_{l\text{2}}L_{st}^{ud}}}\;-\underset{\begin{array}{c} \text{Decay} \end{array}}{\;\underbrace{d_{lr}^{ud}L_{st}^{ud}}} \end{array}$$ (15)

$$\begin{array}{c} \frac{dL_{st}^{d}}{dt}=\underset{\begin{array}{c} \text{Activation}\;\text{to}\;\text{the}\\ \text{L1}\;\text{phenotype} \end{array}}{\underbrace{-R_{l\text{1}}L_{st}^{d}}}\;-\;\underset{\begin{array}{c} \text{Activation}\;\text{to}\;\text{the}\\ \text{L2}\;\text{phenotype} \end{array}}{\underbrace{R_{l\text{2}}L_{st}^{d}}}\;-\;\underset{\begin{array}{c} \text{Decay} \end{array}}{\underbrace{d_{lr}^{d}L_{st}^{d}}} \end{array}$$ (16)

$$\begin{array}{c} \frac{d{L\text{1}}_{tb}}{dt}=\underset{\begin{array}{c} \text{Activation}\;\text{to}\;\text{the}\\ \text{L1}\;\text{phenotype} \end{array}}{\underbrace{R_{l\text{1}}\left(L_{st}^{ud}+L_{st}^{d}\right)}}\;-\;\underset{\begin{array}{c} \text{Decay} \end{array}}{\underbrace{d_{l}L{\text{1}}_{tb}}} \end{array}$$ (17)

$$\begin{array}{c} \frac{d{L\text{2}}_{tb}}{dt}=\underset{\begin{array}{c} \text{Activation}\;\text{to}\;\text{the}\\ \text{L2}\;\text{phenotype} \end{array}}{\underbrace{R_{l\text{2}}\left(L_{st}^{ud}+L_{st}^{d}\right)}}\;-\;\underset{\begin{array}{c} \text{Decay} \end{array}}{\underbrace{d_{l}L{\text{2}}_{tb}}} \end{array}$$ (18)

$$\begin{array}{c} R_{l\text{1}}=\;\underset{\begin{array}{c} \text{Activation}\;\text{by}\;\text{M1}\;\text{macrophages}\\ \text{with}\;\text{inhibition}\;\text{by}\;\text{L2}\;\text{lymphocytes} \end{array}}{\underbrace{\Omega_{i}\left(k_{l\text{1}}M{\text{1}}_{tb};L{\text{2}}_{tb},{L\text{2}}_{\text{1}}^{\infty }\right)}} \end{array}$$ (19)

$$R_{l\text{2}}=\;\underset{\begin{array}{c} \text{Activation}\;\text{by}\;\text{M2}\;\text{macrophages}\\ \text{with}\;\text{inhibition}\;\text{by}\;\text{fibroblasts} \end{array}}{\underbrace{\Omega_{i}\left(k_{l\text{2}}M{\text{2}}_{tb};F_{tb},F^{\infty }\right)}}$$ (20)

Lymphocyte function in the present model consists of general regulation of the macrophage response through inflammatory mediators and other signaling biomolecules. At present, damaged lymphocytes in the surrounding tissue do not affect lymphocyte function or polarization, as was seen in the macrophage equations, but the population of damaged lymphocytes is included due to their importance in models of radiation.

### Fibroblasts

The presence of fibroblasts in the injury site indicates the start of the proliferative phase which is marked by the deposition of new extracellular matrix to replace damaged tissue [40]. Fibroblasts in the surrounding tissue are recruited from the local connective tissues as well as through proliferation of resident populations.


$$\begin{array}{c} \frac{dF_{st}^{ud}}{dt}=\underset{\begin{array}{c} \text{Fibroblast}\;\text{recruitment}\\ \text{from}\;\text{the}\;\text{connective}\\ \text{tissues}\\ \left(\text{inhibited}\;\text{by}\;\text{radiation}\right) \end{array}}{\underbrace{{v\left(t,D_{R};\gamma_{f}\right)\;s}_{f}}}\;\;\;-\underset{\begin{array}{c} \text{Recruitment}\\ \text{of}\;\text{fibroblasts}\\ \text{to}\;\text{the}\;\text{injury}\;\text{site} \end{array}}{\underbrace{k_{sttb}^{ud}F_{st}^{ud}}}\;\;\;\;\;-\;\underset{\begin{array}{c} \text{Decay} \end{array}}{\underbrace{d_{fr}^{ud}F_{st}^{ud}}} \end{array}$$ (21)

$$\begin{array}{c} \frac{dF_{st}^{d}}{dt}=\;\underset{\begin{array}{c} \text{Recruitment}\\ \text{of}\;\text{fibroblasts}\\ \text{to}\;\text{the}\;\text{injury}\;\text{site} \end{array}}{\underbrace{-k_{sttb}^{d}F_{st}^{d}}}\;-\;\underset{\begin{array}{c} \text{Decay} \end{array}}{\underbrace{d_{fr}^{d}F_{st}^{d}}} \end{array}$$ (22)

$$\begin{array}{cc} \frac{dF_{tb}}{dt} & =\underset{\begin{array}{c} \text{Proliferation}\;\text{of}\;\text{fibroblasts}\;\text{by}\;\text{other}\;\text{fibroblasts},\;\text{damaged}\;\text{tissue},\;\text{and}\;\text{M2}\;\text{macrophages}\\ \left(\text{inhibited}\;\text{by}\;\text{inflammatory}\;\text{cells}\;\text{and}\;\text{radiation}\right) \end{array}}{\underbrace{{\mathrm{\backslash itOmega}}_{i}\left(\text{1};F_{st}^{d},\omega_{f}\right){\mathrm{\backslash itOmega}}_{i}\left(F_{tb};N_{tb},N_{\text{2}}^{\infty },M{\text{1}}_{tb},{M\text{1}}_{\text{2}}^{\infty }\right)\left(k_{f}+{\alpha_{dam}Dam_{tb}+\alpha }_{m\text{2}}M{\text{2}}_{tb}\right)}}\\ & \;+\underset{\begin{array}{c} \text{Recruitment}\;\text{of}\;\text{fibroblasts}\\ \text{to}\;\text{the}\;\text{injury}\;\text{site} \end{array}}{\underbrace{k_{sttb}^{ud}F_{st}^{ud}+k_{sttb}^{d}F_{st}^{d}}}-\underset{\begin{array}{c} \text{Decay} \end{array}}{\underbrace{d_{f}F_{tb}}} \end{array}$$ (23)

Recruitment of fibroblasts from the surrounding tissue is inhibited like the inflammatory cells in a dose-dependent manner to account for the microvascular damage associated with local tissue injury from radiation. Fibroblast proliferation is also inhibited by radiation injury as this has been observed to reduce the proliferative abilities [23,51]. Similar to previous equations, we use the population of damaged fibroblasts to drive this inhibition.

Equation 22 has an analytical solution solved using separation of variables and simple integration. Since this equation only has constant parameter rates, the final solution is only dependent on time. The solution is represented in Equation 24 and was used throughout simulations in place of Equation 22.


$$F_{st}^{d}(t)=F_{st,\text{0}}^{d}e^{-\left(k_{sttb}^{d}+d_{fr}^{d}\right)t}$$ (24)

### Pathogen

Ionizing radiation is associated with an increased risk of infection regardless of a localized [52] or whole-body exposure [53–56]. This risk is observed to be further elevated for irradiated burns [7] so pathogen dynamics must be accounted for in the model. The pathogen equation was generated using a common equation structure for bacterial populations [30]. The equation includes logistic growth and removal by phagocytosis from the model’s inflammatory cells and other background responses (e.g., mast cells and natural killer cells).


$$\begin{array}{cc} \frac{dP_{tb}}{dt} & =\underset{\begin{array}{c} \text{Logistic}\;\text{growth}\;\text{of}\\ \text{pathogen} \end{array}}{\underbrace{k_{pg}P_{tb}\left(\text{1}-\frac{P_{tb}}{P^{\infty }}\right)}}-\underset{\begin{array}{c} \text{Phagocytosis}\;\text{by}\\ \text{background}\\ \text{inflammatory}\\ \text{response} \end{array}}{\underbrace{\frac{k_{pb}s_{b}P_{tb}}{\mu_{b}+k_{bp}P_{tb}}}}-\underset{\begin{array}{c} \text{Phagocytosis}\\ \text{by}\;\text{neutrophils} \end{array}}{\underbrace{{k_{pn}P}_{tb}N_{tb}}}\\ & \;-\underset{\begin{array}{c} \text{Phagocytosis}\;\text{by}\;\text{M1}\\ \text{macrophages}\\ \left(\text{inhibited}\;\text{by}\;\text{neutrophils}\right) \end{array}}{\underbrace{k_{pm\text{1}}P_{tb}{\mathrm{\backslash itOmega}}_{i}\left({M\text{1}}_{tb};N_{tb},N_{\text{1}}^{\infty }\right)}}-\underset{\begin{array}{c} \text{Phagocytosis}\;\text{by}\;\text{M2}\\ \text{macrophages}\\ \left(\text{inhibited}\;\text{by}\;\text{neutrophils}\right) \end{array}}{\underbrace{k_{pm\text{2}}P_{tb}{\mathrm{\backslash itOmega}}_{i}\left({M\text{2}}_{tb};N_{tb},N_{\text{1}}^{\infty }\right)}} \end{array}$$ (25)

### Initial conditions

The model variables are initialized using a combination of thermal fluence, ionizing radiation dose, and steady state calculations. The cellular damage in the thermal burn injury is assumed to be a result of thermal fluence only and the damage in the surrounding tissue is assumed to be a result of radiation exposure only.

### Surrounding tissue compartment. 

The surrounding tissue compartment contains tissue resident immune cell populations and fibroblasts unaffected by the burn injury. Neutrophils have not been evidenced to have tissue resident populations [57], so this variable is initialized at zero. Monocytes, lymphocytes, and fibroblasts do have tissue resident populations at a homeostatic steady state within the system. The steady value for these variables is the ratio of the constant influx rate from the blood vessels to their decay rate. Thus, the steady state in the surrounding tissue for monocytes is represented by $\frac{s_{mr}}{d_{mr}}$ and the steady state for lymphocytes is represented by $\frac{s_{lr}}{d_{lr}}.$ The fibroblast population will approach the ratio of the influx rate from the connective tissues and proliferation in the surrounding tissue to the difference of the decay rate and the rate at which fibroblasts are recruited to the thermal burn compartment. Thus, the steady state in the surrounding tissue for fibroblasts is represented by$\frac{s_{f}}{\left(d_{fr}-k_{ftb}^{ud}\right)}.$

The steady state values calculated for the specific cell populations are then split between the undamaged and damaged variables dependent on the ionizing radiation dose. The proportion that is represented by the undamaged model variable is determined from linear quadratic cell survivability curves fit to experimental data [58–61]. The linear quadratic model was fit to experimental data using the fminsearch function in MATLAB using an objective function of the residual sum of squares between the model and the data and the default algorithm parameters for convergence. Calculations for the resulting initial conditions are show in Table 2 using the optimized parameters. The variable $D_{R}$ represents the radiation dose in Gy.

**Table 2:** Initial conditions for the variables in the surrounding tissue compartment.

Variable	Initial Condition
${\overset{―}{M}}_{st}^{ud}$	${\overset{―}{M}}_{st,\text{0}}(e^{-\text{0}.\text{1}\text{8}\text{2}\text{6}D_{R}})$
${\overset{―}{M}}_{st}^{d}$	${\overset{―}{M}}_{st,\text{0}}(\text{1}-e^{-\text{0}.\text{1}\text{8}\text{2}\text{6}D_{R}})$
$L_{st}^{ud}$	$L_{st,\text{0}}(e^{-\text{0}.\text{3}\text{4}\text{8}\text{1}D_{R}-\text{0}.\text{0}\text{7}\text{2}\text{3}D_{R}^{\text{2}}})$
$L_{st}^{d}$	$L_{st,\text{0}}(\text{1}-e^{-\text{0}.\text{3}\text{4}\text{8}\text{1}D_{R}-\text{0}.\text{0}\text{7}\text{2}\text{3}D_{R}^{\text{2}}})$
$F_{st}^{ud}$	$F_{st,\text{0}}(e^{-\text{0}.\text{5}\text{0}\text{8}\text{4}D_{R}-\text{0}.\text{0}\text{5}\text{4}\text{9}D_{R}^{\text{2}}})$
$F_{st}^{d}$	$F_{st,\text{0}}(\text{1}-e^{-\text{0}.\text{5}\text{0}\text{8}\text{4}D_{R}-\text{0}.\text{0}\text{5}\text{4}\text{9}D_{R}^{\text{2}}})$

The undamaged cell populations are initialized using the proportion of surviving cells and the damaged cells are initialized using one minus the proportion of surviving cells, as calculated by the respective linear quadratic model. State variables are assumed to be at steady state prior to and following irradiated thermal injury. Steady state values for macrophages (${\overset{―}{M}}_{st,\text{0}}$), lymphocytes ($L_{st,\text{0}}$), and fibroblasts ($F_{st,\text{0}}$) are strictly positive constants such that each is equal to its corresponding healed steady-state value.

### Thermal burn compartment. 

The thermal burn compartment includes the variables for damage and debris, the various inflammatory cells, fibroblasts, and pathogen. The damage and debris variables describe the severity of thermal injury and are initialized using thermal fluence alone. There is likely some radiation injury that occurs, but we assume that the thermal injury to cells causes complete cell death and thus supersedes any complete or partial DNA damage from ionizing radiation exposure. The population of inflammatory cells and fibroblasts are initialized to zero since we assume no viable cells will remain in the burn site.

Burn injuries considered in this model are caused by the direct absorption of radiant energy into the skin, referred to as flash burns [62]. The severity of the flash burn is used to initialize the damage and debris variables by linking the thermal fluence to a corresponding burn severity. To create a mapping of thermal fluence to burn severity, we estimated the deposition of radiant energy into the different layers of skin tissue, obtained a solution to the resultant bioheat transfer equation [10], and subsequently employed the Arrhenius damage equation [11] to determine the depth of severe cellular damage. Further details on this can be found in S5 File.

In our model, we associated the initial condition $Dam_{tb}(\text{0})=Deb_{tb}(\text{0})=\text{0}.\text{1}$ with a superficial thermal burn and $Dam_{tb}(\text{0})=Deb_{tb}(\text{0})=\text{0}.\text{9}$ with a superficial partial-thickness thermal burn. Due to the different cellular compositions of the epidermis and dermis, we developed a piecewise equation to model the level of tissue damage within the two layers. Equation 27 directly maps thermal fluence (*f*) to the expected damage constant for the equations for cellular damage and debris.


$$\begin{array}{c} Dam_{tb}[\text{0}](f)=\left\{ \begin{array}{cc} \text{0} & f<\text{5}.\text{0}\text{1}\text{6}\\ \frac{\text{1}\text{0}\text{0}}{\text{3}}\left(af^{\text{2}}+bf+c\right) & \text{5}.\text{0}\text{1}\text{6}\leq f\leq \text{5}.\text{1}\text{6}\text{8},\\ \frac{\text{8}\text{0}}{\text{3}}\left(af^{\text{2}}+bf+c\right)+\text{0}.\text{0}\text{2} & f>\text{5}.\text{1}\text{6}\text{8} \end{array}\right. \end{array}$$ (26)

where $a=-\text{8}.\text{9}\text{7}\text{9}\text{5}\times {\text{1}\text{0}}^{-\text{5}}$, $b=\text{7}.\text{2}\text{0}\text{1}\text{9}\times {\text{1}\text{0}}^{-\text{3}}$, and $c=-\text{3}.\text{1}\text{8}\text{2}\text{2}\times {\text{1}\text{0}}^{-\text{2}}$.

Pathogen is initialized using a combination of burn severity and radiation exposure. Radiation injury is associated with a risk of bacterial infiltration leading to infection [7,52,55] and we assume that more severe burns will provide greater opportunity for pathogen exposure from external sources. For ionizing radiation associated with concomitant wound infection, the organisms that cause most of the injury infections are skin flora organisms (*staphylococci* and *streptococci*) or environmentally acquired bacteria [63]. These bacteria are not significantly affected by ionizing radiation at the level considered in this model [64,65], so we assume that the ambient population is not reduced from the radiation environment.

In previous mathematical models featuring a pathogen population, the pathogen carrying capacity was typically used as the initial condition [29]. Risk and severity of infection-causing bacteria, however, varies directly with burn severity and radiation exposure [66,67]. We assume that there is no pathogen population when radiation exposure is not present, since superficial thermal burns alone are not associated with a significant risk of infection [66]. When radiation exposure does occur, we initialize the pathogen variable on a scale between zero and the carrying capacity where 0 is associated with no thermal injury and the carrying capacity, $P^{\infty }$, is associated with the most severe thermal injury considered here, associated with a thermal fluence of 19 J/cm^2^. Thus, we define the initial conditions for $P_{tb}$ using Equation 27 where $P_{tb,\text{0}}$ is the initial condition for the pathogen variable, $Dam_{tb,\text{0}}$ is the initial condition for the damage variable, and $Dam_{tb,max}$ is the initial condition for the damage variable associated with a thermal fluence of 19 J/cm^2^. This scheme ensures that more severe burns are associated with a higher initial bacterial load and a prolonged pathogen presence. This acts as additional stress on the inflammatory system and corresponds with the increased immunesuppression and bacterial risk as burn severity increases [68].


$$P_{tb,\text{0}}\left(Dam_{tb,\text{0}},D_{R}\right)=\left\{ \;\;\;\begin{array}{c} \;\;\;\;\;\;\text{0},\;\;\;\;\;\;\;\;\;\;\;\;\;\;\;\;\;\;\;\;\;\;\;\;\;\;\;\;\;\;\;\;\;\;D_{R}=\text{0}\;\\ P^{\infty }(Dam_{tb,\text{0}}/Dam_{tb,max}),\;\;\;\;D_{R}>\text{0} \end{array}\right.\;$$ (27)

### Sensitivity analysis and parameter selection

The model contains a large number of unknown parameters which must be carefully selected to ensure biological feasibility. Due to the nature of the injury profile, data either does not exist or is extremely limited. Sensitivity analyses and leveraging of minimal data and biological trends were combined to select plausible parameter sets for the model. For sensitivity analysis, we employed the global method Multi-test Extended Fourier Amplitude Sensitivity Test (MeFAST) [69], which uses the Extended Fourier Amplitude Sensitivity Test (eFAST) algorithm [70,71] along with the t-test suggested in Marino et al. [72], the analysis of variance (ANOVA) with the Tukey procedure, and the Wilcoxon rank sum test utilized in Dela et al. [69].

Parameter combinations were initially generated using Latin hypercube sampling (LHS). For each parameter combination, the model was simulated for both a superficial thermal burn and a superficial partial-thickness thermal burn, as defined by the initial conditions of the state variables for damage and debris. Parameter combinations were assessed for three criteria, reflecting biological feasibility of the model predictions: (a) the simulation produced no numerical errors, (b) debris resolved before damage, and (c) the superficial thermal burn healed within 4.667±0.5 days and the superficial partial-thickness burn healed within 7.833±0.5 days [66,67,73,74]. The parameter minimum and maximum values for the eFAST algorithm were calculated from the sets satisfying these criteria.

To perform the sensitivity analysis, the model was simulated at five time points corresponding to days one, three, seven, ten, and fourteen post-injury. These are common data points used for collection in experimental models of thermal burn injury [75] and they correspond with identified response and peak times for neutrophils (1–7 days) and macrophages (3–14 days). Radiation levels were also varied using 1, 7, and 14 Gy. Sensitivity indices were calculated for varying values set for the number of samples per search curve ($N_{S}$) and the resampling size ($N_{R}$) to ensure the selection did not significantly influence the algorithm results. The largest values for $N_{S}$ and $N_{R}$, 2000 and 32, respectively, were used in the final selection algorithm.

The sensitivity analysis results were used to reduce the estimated parameter space and aid in selection of a cohort of feasible parameter sets as well as a representative set of parameters that is indicative of the average model behavior. We developed an algorithm to select which parameters were considered highly influential (shown in S3 Fig). Those selected (shown in Table 3) were further sampled and outputs were assessed for biological feasibility. Non-influential parameters were set to their baseline value as determined by the average value of the initial sample range. Additional sampling details, including parameter inequalities that help to ensure stability of the healed state, can be found in S4 File.

**Table 3:** Parameters selected to be estimated or fixed using the sensitivity analysis results and the parameter selection algorithm.

PARAMETERS TO ESTIMATE	PARAMETERS TO FIX
***Inhibition***	
$N_{\text{1}}^{\infty }$, ${M\text{1}}_{\text{1}}^{\infty }$, $M{\text{2}}^{\infty }$	$Deb_{dam}^{\infty }$, $Deb_{H}$, $M{\text{1}}_{\text{2}}^{\infty }$, $F^{\infty }$, $L{\text{2}}_{\text{1}}^{\infty }$, $L{\text{2}}_{\text{2}}^{\infty }$, $N_{H}$, $M{\text{1}}_{H}$
***Inhibition (radiation-related)***	
$\gamma_{n}$, $\gamma_{m}$, $\omega_{f}$	$\gamma_{f}$, $\gamma_{l}$, $\omega_{m\text{2}}$, $\omega_{dam}$
***Damage and Debris***	
$k_{dnp}$, $k_{dm\text{1}p}$	$k_{dn}$, $\sigma_{dm\text{1}}$, $d_{deb}$, $\rho_{dam}$, $k_{df}$, $k_{dm\text{2}p}$
***Neutrophils***	
$k_{nd}$, $s_{nr}$, $k_{nm\text{1}p}$, $d_{n}$	$k_{np}$, $k_{nn}$, $k_{nm\text{2}p}$, $d_{nr}$
***Macrophages***	
$k_{m\text{1}d}$, $k_{m\text{1}n}$, $k_{m\text{2}m\text{1}}$, $s_{mr}$, $d_{mr}$, $d_{m\text{1}}$, $k_{m\text{1}p}$	$k_{m\text{1}l\text{1}}$, $k_{m\text{1}m\text{1}}$, $k_{m\text{2}m\text{2}}$, $k_{m\text{2}l\text{2}}$, $d_{mrd}$, $d_{m\text{2}}$, $\theta_{m\text{1}m\text{2}}$
***T Lymphocytes***	
$k_{l\text{1}}$	$s_{lr}$, $d_{l}$, $k_{l\text{2}}$, $d_{lr}$, $d_{lrd}$
***Fibroblasts***	
	$s_{f}$, $k_{ftb}^{ud}$, $k_{ftb}^{d}$, $k_{f}$, $\alpha_{dam}$, $d_{f}$, $\alpha_{m\text{2}}$, $d_{fr}$, $d_{frd}$
***Pathogen***	
$k_{pg}$, $P^{\infty }$, $k_{pb}$, $s_{b}$, $k_{bp}$, $k_{pn}$	$\mu_{b}$, $k_{pm\text{1}}$, $k_{pm\text{2}}$

Single parameter sets were solved numerically in approximately 0.05 seconds allowing us to simulate around 400,000 individual samples within a reasonable computational time and then assess the resulting transients for biological feasibility. A representative set was selected from the set of accepted sets by comparing the output of each parameter set to the temporal mean output of each model variable for the entire set of plausible parameter sets. The sets were compared quantitatively using both the weighted mean squared error between each set and the temporal mean as well as the coefficient of determination across all model variables.

## Results

### Model simulations

Model behavior was simulated using the representative set of parameters to demonstrate diverse model trajectories across initial conditions and outcomes associated with both the burn alone and the combined injury profile. The novelty of this model lies in the combined effects of burn with local irradiation, so various levels of radiation were explored. Simulations demonstrated delayed healing time, reduced immune cell infiltration, fibroblast dysfunction, and pathogen dynamics. All simulations use the initial conditions described in the methods section and include comparisons between the single injury (burn only) and the combined injury (burn and radiation).

### Delayed healing times

**Fig 3:**
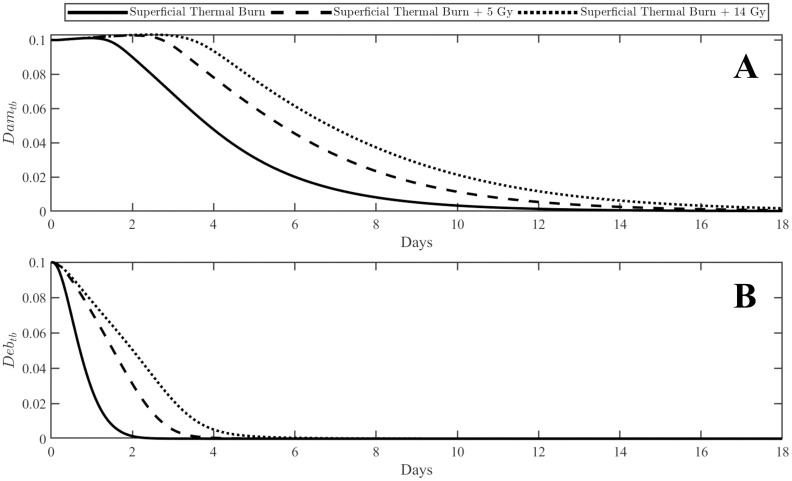
Plots of damage ($Dam_{tb}$) and debris ($Deb_{tb}$) for a superficial thermal burn with varying levels of radiation. Panel A shows $Dam_{tb}$ and panel B shows $Deb_{tb}$. Superficial thermal burns alone should heal fully within 4 to 5 days. The introduction of ionizing radiation delays the immune response and results in prolonged healing times.

An important feature of RCI is delayed healing for wounds [19–21]. This is captured by the model through increased healing times and prolonged presence of debris as shown in Fig 3. Both the 5 Gy and 14 Gy simulations exhibit increased duration of debris (Fig 3B) and slower resolution of damage (Fig 3A). The combined injury profiles also show a larger initial increase in damage as evidenced in Fig 3A by the initial positive slope. For the single injury profile, the initial positive slope resolves around 1.75 days, for the combined profile with 5 Gy, the slope remains positive until approximately 2.5 days and for injury with 14 Gy the slope is positive until around 3 days. This delayed healing is typically a result of delayed and prolonged immune cell infiltration at the wound site [22]. The prolonged presence of inflammatory cells also contributes to the initial increase in damage.

### Reduced immune cell infiltration

**Fig 4:**
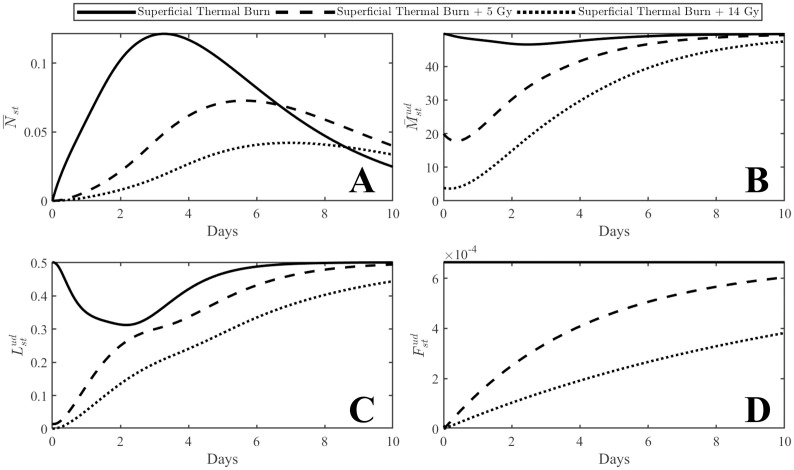
Plots of undamaged cells in the surrounding tissue supplied by the bloodstream. Panel A represents neutrophils, panel B represents macrophages, panel C represents lymphocytes, and panel D represents fibroblasts. Populations in the surrounding tissue are affected by both the initial cellular injury from ionizing radiation as well as reduced influx due to microvasculature damage in the surrounding tissue.

Local radiation injury is known to delay the infiltration of immune cells from the bloodstream and into the wound due to the damage of microvasculature in the affected tissues [24]. This is included in the model by inhibiting influx rates into the surrounding tissue from the bloodstream which results in reduced resource pools available for activation at the wound. Fig 4 shows the cells in the surrounding tissue supplied by the populations in the bloodstream. As shown, combined radiation injury results in reduced initial levels of undamaged cells (due to the initial radiation injury) which is further inhibited by microvasculature damage in the early stages of inflammation (within the first few days). These delays propagate to the corresponding activated populations at the thermal injury site.

For the combined injury profiles, all immune cells in the surrounding tissue remain below the single injury levels, aside from neutrophils. This population returns to zero once the wound is healed; since healing is delayed in the combined injuries, levels remain elevated. An initial dip in the cell population in the surrounding tissue is expected, like those shown in Fig 4B and 4C, since immune cells will be attracted to the injury. In the combined injury profiles, extravasation from the blood stream is necessary to support both activation of immune cells in the wound as well as replacement of the damaged resting population.

**Fig 5:**
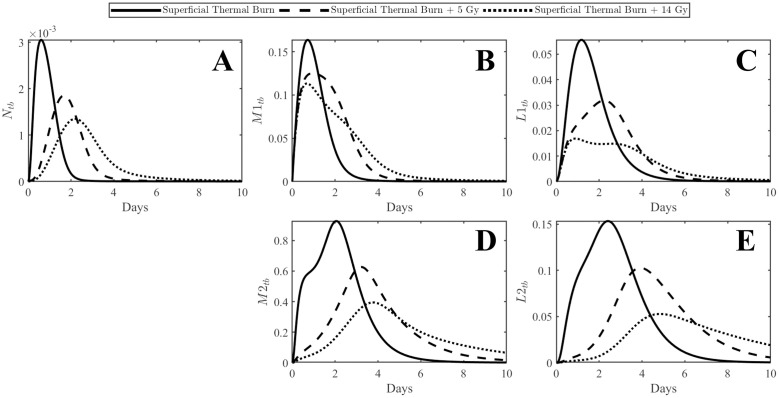
Plots of immune cells in the injury compartment. Panel A represents neutrophils, panel B represents M1 macrophages, panel C represents L1 lymphocytes, panel D represents M2 macrophages, and panel E represents L2 lymphocytes. Immune cell response in the injury is delayed due to ionizing radiation exposure.

Similar trends can be observed in the thermal burn compartment (Fig 5). All variables show marked delays in immune cell peaks as well as reduced magnitude of response when radiation is present. M1 macrophages and L1 lymphocytes actually exhibited some differing behavior with peaks occurring slightly earlier with radiation exposure of 14 Gy. This can be a result of the radiation exposure encouraging an increased inflammatory environment and delaying the switch to anti-inflammatory phenotypes represented as M2 macrophages and L2 lymphocytes. Both are significantly delayed with the 5 Gy and 14 Gy exposures.

### Fibroblast dysfunction

**Fig 6:**
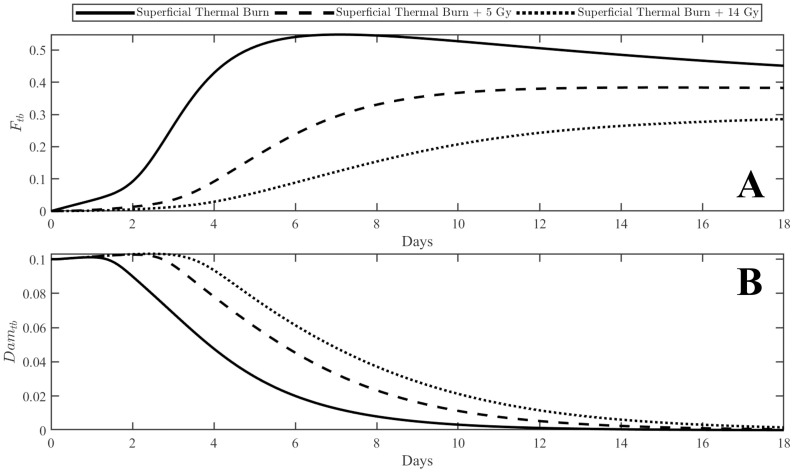
Plots of fibroblasts (F_tb_) and damage (Dam_tb_) in the thermal injury. Ionizing radiation exposure alters fibroblast function resulting in reduced proliferative abilities and irregular collagen deposition, delaying damage resolution.

Ionizing radiation affects dermal fibroblasts by reducing proliferative abilities [23]. Fibroblast dysfunction, along with reduced infiltration as shown in Fig 6, results in reduced fibroblast populations and irregular collagen deposition. As shown, these effects increase with ionizing radiation levels and correlate with delayed healing times.

### Sustained pathogen population

**Fig 7:**
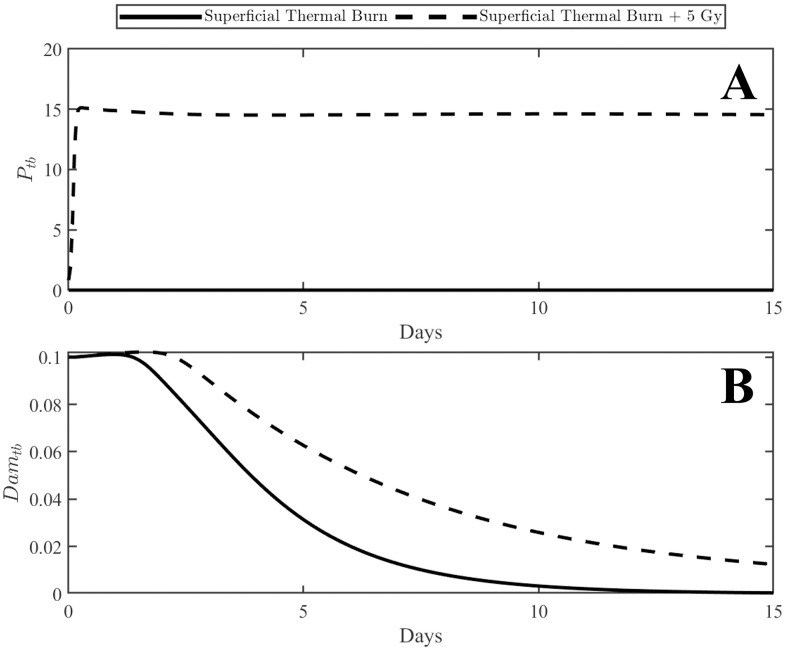
Plots of pathogen (Ptb) and damage (Dam_tb) at the thermal injury site. Since ionizing radiation is associated with risk of bacterial infiltration leading to infection, the model includes the capability to capture prolonged bacterial presence and delayed healing which could result in a non-healing wound and chronic inflammation.

RCI is associated with an increased risk of bacterial infiltration and sustained infection [2,7]. Superficial thermal burns, on the other hand, are not typically associated with a risk of infection [66]. As shown in Fig 7, the model has the capacity to induce prolonged pathogen presence that could result in a systemic infection if spread to the bloodstream. Without resolution of the pathogen variable, the thermal injury will fail to heal, resulting in chronic inflammation.

## Discussion

We developed a model for a combined injury profile consisting of a superficial thermal burn and locally confined ionizing radiation exposure. The model consists of a system of ODEs representing key inflammatory cells and fibroblast populations in the thermal injury and surrounding tissue compartments. A mechanistic model for combined injury provides time-dependent outputs that can capture the synergistic effects of combined injury for a wide range of inputs while also offering pathways for further predictive capabilities associated with the injury. This model serves as an initial exploration into modeling combined injury using a mechanistic model. A mechanistic model presents significant advantages over previously developed empirical models that are only able to predict single variables, such as injury prevalence [27] or probability of survival [28]. Additionally, mechanistic models can be used to better understand the underlying mechanisms involved in RCI, such as specific immune cell dynamics and pathogen dynamics. This model will be further developed to improve predictions of first responder capabilities in a nuclear incident.

The formulated model presents a mechanistic modeling approach, which relies on known biological mechanisms involved in inflammation and early proliferation, as well as experimental observations and assumptions from animal trials and first responder reports from historical events. Due to the nature of the injury, real world data is not available. Despite this, the model still uses a number of qualitative assessments to ensure biological feasibility of the results. The simulations of the single injury profile (burn only) were restricted to those that healed within the previously determined healing times for superficial and superficial partial thickness thermal burns [66,67,73,74]. The model formulation and simulations also rely on known dynamics for inflammation and activation, including both timing of peaks in specific cell populations and interactions between the cells. Observed behavior in the combined injury profile was also controlled for in parameter selection (S4 File) and verified qualitatively in model outputs. While this does not provide quantitative measures of model verification, this form of modeling can still offer useful predictions about the biological behavior that can be further improved if data becomes available for the model outputs or model parameters. The present model serves as the first iteration of this type of modeling which will continue to be improved.

The model was able to recreate various known dynamics of RCI with burn including increased healing times, reduced immune cell infiltration, fibroblast dysfunction, and sustained pathogen. Experimental models for RCI, both with burn [8,76,77], and other injuries (laceration) [78–80], consistently report prolonged healing times and reduced immune cell activity. Fibroblast dysfunction, including both reduced proliferation and irregular collagen deposition, has also been evidenced in the experimental studies [23,51] and further contributes to delayed and deficient wound healing.

Pathogen dynamics are particularly important in combined injury models involving burn injury. In superficial thermal burns alone, there is little to no risk of infection [66,67], but ionizing radiation is associated with a risk of infection regardless of whether there is a localized [52] or whole-body exposure [53–56]. This risk is further elevated for irradiated burns [7]. The majority of accepted parameter sets exhibited quick resolution of any foreign infiltrates, as would be expected for less severe burns, but the model also contains the capacity for a sustained pathogen response (Fig 7). The pathogen equation (Eq. 25) accounts for minor immunosuppression during radiation exposure from reduced availability of macrophages due to cellular death. This, however, has not been accounted for in the background immune response, which is mainly mediated by mast cells and natural killer cells [81]. For this reason, the current formulation may not accurately predict sepsis risk and should be revisited in future iterations and when considering burns with larger total body surface area and/or penetration depth or those with whole-body radiation exposure. Since infection observed in radiation injury could also be the result of bacteria entering the blood stream from damage to the gastrointestinal tract [82], it is important to continue to consider pathogen dynamics when models of whole-body radiation exposures are considered for RCI.

Our modeling approach does have some limitations. First, this initial model formulation contains some necessary assumptions to support modeling, including instantaneous exposure to thermal and ionizing radiation at the injury site and some cell based assumptions. The present model serves as the first iteration of this type of approach for combined injury and will continue to be improved upon. Second, the model is confined to the local domain. This prevents the model from representing more severe injuries that result in systemic effects [3,76] and whole-body radiation which results in hematopoietic disruption and additional systemic effects [18,82]. Third, the model does not account for additional injuries, such as lacerations or fractures, which can put additional stress on the body, potentially exacerbating immune response delays. Fourth, the model does not account for specifics of the thermal burn including location and percent total body surface area. Since we assume the thermal injury does not initiate a systemic response, percent total body surface area is assumed to be relatively low. Location is also important in determining burn severity since burns are typically more severe on the face and hands.

Despite its limitations, the model can still offer useful applications. One application we’ve explored is using immune cell levels at the wound site to predict medical outcomes, namely hyperalgesia. The model variables describing the inflammatory response can be used to predict the experience of inflammatory pain by estimating the biological concentration of signals detected by nociceptors, sensory neurons that respond to damaging stimuli. Inflammatory pain is associated with tissue damage where signaling biomolecules are released from damaged cells and inflammatory cells which prime nociceptors and increase the sensitivity of the nerve endings [83]. The signaling molecules causing an inflammatory pain response and hyperalgesia include IL-1β, TNF-α, and IL-6, which are mediated and released by active inflammatory cells. Model variables can estimate this response and estimate the occurrence of pain in RCI profiles.

To further develop the combined injury framework, future models should extend to systemic injuries including both more severe burn injuries and widespread radiation exposure. When considering widespread radiation, the hematopoietic system and gastrointestinal tract are particularly sensitive and should be included in whole-body exposure models. Systemic injuries would also allow for the estimation of systemic symptoms affecting medical outcomes. This could include fatiguability and weakness which largely result from increased circulating cytokines [84] and upper-gastrointestinal distress which results from major cell death in intestinal crypts [82] and serotonin release in the gut [85–87]. Mechanistic models for RCI can be further developed to include these systemic effects and serve as sophisticated modeling tools to estimate casualties and capabilities of first responders in the event of nuclear incident.

## Conclusions

Radiation combined injuries present a formidable modeling problem since single injury and empirical models are unable to properly capture the synergistic effects associated with concurrent injuries and provide accurate extrapolatory predictions. We developed a mechanistic model of RCI including local radiation exposure and burn injury which allows for estimation of injury effects outside the restriction of available data. A mechanistic model also provides a means to explore medical outcomes associated with the inflammatory response that could affect first responder performance. This model serves as a basis for developing mechanistic combined injury models of systemic injury associated with whole-body radiation exposure and more severe injuries such as larger burns and major fractures. Further development to include systemic effects would improve estimations, providing tools for medical planning and triage in a nuclear incident.

## Supporting information

S1 Table Parameters with descriptions and selected values. Model parameters including descriptions and units. The listed numerical value was determined by the representative set.
(PDF)

S2 File Detailed description of model equations. (PDF)

S3 Fig Flowchart of parameter selection algorithm to determine whether a parameter should be fixed or further sampled. (TIF)

S4 File Parameter sampling and acceptance criteria. (PDF)

S5 File Mapping thermal fluence to damage and debris. (PDF)

S6 File Sensitivity analysis results. (PDF)

## References

[1] DiCarlo AL, Hatchett RJ, Kaminski JM, Ledney GD, Pellmar TC, Okunieff P, et al. Medical countermeasures for radiation combined injury: radiation with burn, blast, trauma and/or sepsis. report of an NIAID Workshop, March 26-27, 2007. Radiat Res. 2008;169(6):712–21. doi: 10.1667/RR1295.1 18494548 PMC8409135

[2] DiCarlo AL, Ramakrishnan N, Hatchett RJ. Radiation combined injury: overview of NIAID research. Health Phys. 2010;98(6):863–7. doi: 10.1097/HP.0b013e3181a6ee32 20445395 PMC8771911

[3] Pellmar TC, Ledney GD. Combined Injury: Radiation in Combination with Trauma, Infectious Disease, or Chemical Exposures. NATO TRG. 2005.

[4] Antonic V, Jackson IL, Ganga G, Shea-Donohue T, Vujaskovic Z. Development of A Novel Murine Model of Combined Radiation and Peripheral Tissue Trauma Injuries. Radiat Res. 2017;187(2):241–50. doi: 10.1667/RR14557.1 28118112 PMC5414022

[5] Cheng T, Chen Z, Yan Y, Ran X, Su Y, Ai G. Experimental studies on the treatment and pathological basis of combined radiation and burn injury. Chin Med J (Engl). 2002;115(12):1763–6. 12622919

[6] Kiang JG, Jiao W, Cary LH, Mog SR, Elliott TB, Pellmar TC, et al. Wound trauma increases radiation-induced mortality by activation of iNOS pathway and elevation of cytokine concentrations and bacterial infection. Radiat Res. 2010;173(3):319–32. doi: 10.1667/RR1892.1 20199217 PMC10113926

[7] Ledney GD, Madonna GS, Elliott TB, Moore MM, Jackson WE 3rd. Therapy of infections in mice irradiated in mixed neutron/photon fields and inflicted with wound trauma: a review of current work. Radiat Res. 1991;128(1 Suppl):S18–28. doi: 10.2307/3577997 1924743

[8] Yan Y, Ran X, Wei S. Changes of immune functions after radiation, burns and combined radiation-burn injury in rats. Chin Med Sci J. 1995;10(2):85–9. 7647325

[9] Elliott TB, Bolduc DL, Ledney GD, Kiang JG, Fatanmi OO, Wise SY, et al. Combined immunomodulator and antimicrobial therapy eliminates polymicrobial sepsis and modulates cytokine production in combined injured mice. Int J Radiat Biol. 2015;91(9):690–702. doi: 10.3109/09553002.2015.1054526 25994812 PMC4673550

[10] Dai W, Wang H, Jordan PM, Mickens RE, Bejan A. A mathematical model for skin burn injury induced by radiation heating. International Journal of Heat and Mass Transfer. 2008;51(23–24):5497–510. doi: 10.1016/j.ijheatmasstransfer.2008.01.006

[11] Askarizadeh H, Ahmadikia H. Analytical study on the transient heating of a two-dimensional skin tissue using parabolic and hyperbolic bioheat transfer equations. Applied Mathematical Modelling. 2015;39(13):3704–20. doi: 10.1016/j.apm.2014.12.003

[12] Yang Q, Berthiaume F, Androulakis IP. A quantitative model of thermal injury-induced acute inflammation. Math Biosci. 2011;229(2):135–48. doi: 10.1016/j.mbs.2010.08.003 20708022 PMC3239409

[13] Richard M, Kirkby KJ, Webb RP, Kirkby NF. A mathematical model of response of cells to radiation. Nuclear Instruments and Methods in Physics Research Section B: Beam Interactions with Materials and Atoms. 2007;255(1):18–22. doi: 10.1016/j.nimb.2006.11.077 PMC2151736 19050735

[14] Bodgi L, Canet A, Pujo-Menjouet L, Lesne A, Victor J-M, Foray N. Mathematical models of radiation action on living cells: From the target theory to the modern approaches. A historical and critical review. J Theor Biol. 2016;394:93–101. doi: 10.1016/j.jtbi.2016.01.018 26807808

[15] Manabe Y, Ichikawa K, Bando M. A Mathematical Model for Estimating Biological Damage Caused by Radiation. J Phys Soc Jpn. 2012;81(10):104004. doi: 10.1143/jpsj.81.104004

[16] Jones TD, Morris MD, Young RW. A Mathematical Model for Radiation-Induced Myelopoiesis. Radiation Research. 1991;128(3):258. doi: 10.2307/3578048 1961922

[17] Zukhbaya TM, Smirnova OA. An experimental and mathematical analysis of lymphopoiesis dynamics under continuous irradiation. Health Phys. 1991;61(1):87–95. doi: 10.1097/00004032-199107000-00009 1829440

[18] Smirnova OA. Comparative analysis of the dynamics of thrombocytopoietic, granulocytopoietic, and erythropoietic systems in irradiated humans: a modeling approach. Health Phys. 2012;103(6):787–801. doi: 10.1097/HP.0b013e31826021bb 23111526

[19] Stromberg LW, Woodward KT, Mahin DT, Donati RM. Combined surgical and radiation injury. The effect of timing of wounding and whole body gamma irradiation on 30 day mortality and rate of wound contracture in the rodent. Ann Surg. 1968;167(1):18–22. doi: 10.1097/00000658-196801000-00003 5635182 PMC1387210

[20] Zelman D, Song IC, Porteous DD, Bromberg BE. The effect of total body irradiation on wound healing and the hematopoietic system in mice. Bull N Y Acad Med. 1969;45(3):293–300. 5251250 PMC1750260

[21] Zawaski JA, Yates CR, Miller DD, Kaffes CC, Sabek OM, Afshar SF, et al. Radiation combined injury models to study the effects of interventions and wound biomechanics. Radiat Res. 2014;182(6):640–52. doi: 10.1667/RR13751.1 25409125

[22] Vegesna V, Withers HR, Holly FE, McBride WH. The effect of local and systemic irradiation on impairment of wound healing in mice. Radiat Res. 1993;135(3):431–3. doi: 10.2307/3578885 8378536

[23] Qu J, Cheng T, Shi C, Lin Y, Ran X. A study on the activity of fibroblast cells in connection with tissue recovery in the wounds of skin injury after whole-body irradiation. J Radiat Res. 2004;45(2):341–4. doi: 10.1269/jrr.45.341 15304979

[24] Yang X, Ren H, Guo X, Hu C, Fu J. Radiation-induced skin injury: pathogenesis, treatment, and management. Aging (Albany NY). 2020;12(22):23379–93. doi: 10.18632/aging.103932 33202382 PMC7746368

[25] Sweeney JF, Nguyen PK, Omann GM, Hinshaw DB. Ultraviolet irradiation accelerates apoptosis in human polymorphonuclear leukocytes: protection by LPS and GM-CSF. J Leukoc Biol. 1997;62(4):517–23. doi: 10.1002/jlb.62.4.517 9335323

[26] Iddins CJ, DiCarlo AL, Ervin MD, Herrera-Reyes E, Goans RE. Cutaneous and local radiation injuries. J Radiol Prot. 2022;42(1). doi: 10.1088/1361-6498/ac241a 34488201 PMC8785213

[27] Knebel AR, Coleman CN, Cliffer KD, Murrain-Hill P, McNally R, Oancea V, et al. Allocation of scarce resources after a nuclear detonation: setting the context. Disaster Med Public Health Prep. 2011;5 Suppl 1:S20–31. doi: 10.1001/dmp.2011.25 21402809

[28] Casagrande R, Wills N, Kramer E, Sumner L, Mussante M, Kurinsky R, et al. Using the model of resource and time-based triage (MORTT) to guide scarce resource allocation in the aftermath of a nuclear detonation. Disaster Med Public Health Prep. 2011;5 Suppl 1:S98–110. doi: 10.1001/dmp.2011.16 21402818

[29] Torres M, Wang J, Yannie PJ, Ghosh S, Segal RA, Reynolds AM. Identifying important parameters in the inflammatory process with a mathematical model of immune cell influx and macrophage polarization. PLoS Comput Biol. 2019;15(7):e1007172. doi: 10.1371/journal.pcbi.1007172 31365522 PMC6690555

[30] Reynolds A, Rubin J, Clermont G, Day J, Vodovotz Y, Bard Ermentrout G. A reduced mathematical model of the acute inflammatory response: I. Derivation of model and analysis of anti-inflammation. J Theor Biol. 2006;242(1):220–36. doi: 10.1016/j.jtbi.2006.02.016 16584750

[31] Minucci S, Heise RL, Valentine MS, Kamga Gninzeko FJ, Reynolds AM. Mathematical modeling of ventilator-induced lung inflammation. J Theor Biol. 2021;526:110738. doi: 10.1016/j.jtbi.2021.110738 33930440 PMC8277755

[32] Segal RA, Diegelmann RF, Ward KR, Reynolds A. A differential equation model of collagen accumulation in a healing wound. Bull Math Biol. 2012;74(9):2165–82. doi: 10.1007/s11538-012-9751-z 22810171

[33] Su B, Zhou W, Dorman KS, Jones DE. Mathematical Modelling of Immune Response in Tissues. Computational and Mathematical Methods in Medicine. 2008;10(1):9–38. doi: 10.1080/17486700801982713

[34] Cooper AK, Kim PS. A Cellular Automata and a Partial Differential Equation Model of Tumor–Immune Dynamics and Chemotaxis. Springer Proceedings in Mathematics & Statistics. Springer New York. 2014:21–46. doi: 10.1007/978-1-4939-1793-8_2

[35] Chiacchio F, Pennisi M, Russo G, Motta S, Pappalardo F. Agent-based modeling of the immune system: NetLogo, a promising framework. Biomed Res Int. 2014;2014:907171. doi: 10.1155/2014/907171 24864263 PMC4016927

[36] Folcik VA, Broderick G, Mohan S, Block B, Ekbote C, Doolittle J, et al. Using an agent-based model to analyze the dynamic communication network of the immune response. Theor Biol Med Model. 2011;8:1. doi: 10.1186/1742-4682-8-1 21247471 PMC3032717

[37] Folcik VA, An GC, Orosz CG. The Basic Immune Simulator: an agent-based model to study the interactions between innate and adaptive immunity. Theor Biol Med Model. 2007;4:39. doi: 10.1186/1742-4682-4-39 17900357 PMC2186321

[38] Schultz GS, Chin GA, Moldawer L, Diegelmann RF. Principles of Wound Healing. University of Adelaide Press. 2011. 30485016

[39] Namas RA, Mi Q, Namas R, Almahmoud K, Zaaqoq AM, Abdul-Malak O, et al. Insights into the Role of Chemokines, Damage-Associated Molecular Patterns, and Lymphocyte-Derived Mediators from Computational Models of Trauma-Induced Inflammation. Antioxid Redox Signal. 2015;23(17):1370–87. doi: 10.1089/ars.2015.6398 26560096 PMC4685502

[40] Diegelmann RF, Evans MC. Wound healing: an overview of acute, fibrotic and delayed healing. Front Biosci. 2004;9:283–9. doi: 10.2741/1184 14766366

[41] Dormand E-L, Banwell PE, Goodacre TEE. Radiotherapy and wound healing. Int Wound J. 2005;2(2):112–27. doi: 10.1111/j.1742-4801.2005.00079.x 16722862 PMC7951225

[42] Liu X, Liu J-Z, Zhang E, Li P, Zhou P, Cheng T-M, et al. Impaired wound healing after local soft x-ray irradiation in rat skin: time course study of pathology, proliferation, cell cycle, and apoptosis. J Trauma. 2005;59(3):682–90. 16361913

[43] Duffield JS. The inflammatory macrophage: a story of Jekyll and Hyde. Clinical Science. 2002;104(1):27–38. doi: 10.1042/cs1040027 12519085

[44] Rödel F, Frey B, Multhoff G, Gaipl U. Contribution of the immune system to bystander and non-targeted effects of ionizing radiation. Cancer Lett. 2015;356(1):105–13. doi: 10.1016/j.canlet.2013.09.015 24139966

[45] Schäffer M, Weimer W, Wider S, Stülten C, Bongartz M, Budach W, et al. Differential Expression of Inflammatory Mediators in Radiation-Impaired Wound Healing. Journal of Surgical Research. 2002;107(1):93–100. doi: 10.1006/jsre.2002.6494 12384069

[46] Kiang JG, Garrison BR, Gorbunov NV. Radiation Combined Injury: DNA Damage, Apoptosis, and Autophagy. Adapt Med. 2010;2(1):1–10. doi: 10.4247/AM.2010.ABA004 34616567 PMC8491956

[47] DiCarlo AL, Bandremer AC, Hollingsworth BA, Kasim S, Laniyonu A, Todd NF, et al. Cutaneous Radiation Injuries: Models, Assessment and Treatments. Radiat Res. 2020;194(3):315–44. doi: 10.1667/RADE-20-00120.1 32857831 PMC7525796

[48] Loots MA, Lamme EN, Zeegelaar J, Mekkes JR, Bos JD, Middelkoop E. Differences in cellular infiltrate and extracellular matrix of chronic diabetic and venous ulcers versus acute wounds. J Invest Dermatol. 1998;111(5):850–7. doi: 10.1046/j.1523-1747.1998.00381.x 9804349

[49] Ionizing Radiation, Levels and Effects, United Nations Scientific Committee on the Effects of Atomic Radiation (UNSCEAR) 1972 Report. UN. 1972. doi: 10.18356/5513731d-en

[50] Heylmann D, Ponath V, Kindler T, Kaina B. Comparison of DNA repair and radiosensitivity of different blood cell populations. Sci Rep. 2021;11(1):2478. doi: 10.1038/s41598-021-81058-1 33510180 PMC7843614

[51] Rudolph R, Vande Berg J, Schneider JA, Fisher JC, Poolman WL. Slowed growth of cultured fibroblasts from human radiation wounds. Plast Reconstr Surg. 1988;82(4):669–77. doi: 10.1097/00006534-198810000-00019 3420190

[52] Hill A, Hanson M, Bogle MA, Duvic M. Severe radiation dermatitis is related to Staphylococcus aureus. Am J Clin Oncol. 2004;27(4):361–3. doi: 10.1097/01.coc.0000071418.12121.c2 15289728

[53] Elliott TB, Brook I, Stiefel SM. Quantitative study of wound infection in irradiated mice. Int J Radiat Biol. 1990;58(2):341–50. doi: 10.1080/09553009014551671 1974580

[54] Landauer MR, Elliott TB, King GL, Bouhaouala SS, Wilhelmsen CL, Ferrell JL, et al. Performance Decrement after Combined Exposure to Ionizing Radiation and Shigella sonnei. Military Medicine. 2001;166(suppl_2):71–3. doi: 10.1093/milmed/166.suppl_2.71 11778444

[55] Ran X, Cheng T, Lin Y, Qu J, Liu D, Ai G, et al. Dose-effect relationships in total body irradiation on the healing of cutaneous wounds. Chin Med J (Engl). 2003;116(6):878–82. 12877799

[56] Carter SR, Chen MM, Palmer JL, Wang L, Ramirez L, Plackett TP, et al. Neutrophil Accumulation in the Small Intestine Contributes to Local Tissue Destruction Following Combined Radiation and Burn Injury. J Burn Care Res. 2016;37(2):97–105. doi: 10.1097/BCR.0000000000000220 25501789 PMC4465066

[57] Nguyen AV, Soulika AM. The Dynamics of the Skin’s Immune System. Int J Mol Sci. 2019;20(8):1811. doi: 10.3390/ijms20081811 31013709 PMC6515324

[58] Geara FB, Peters LJ, Ang KK, Wike JL, Sivon SS, Guttenberger R, et al. Intrinsic radiosensitivity of normal human fibroblasts and lymphocytes after high- and low-dose-rate irradiation. Cancer Res. 1992;52(22):6348–52. 1423281

[59] Kushiro J, Nakamura N, Kyoizumi S, Nishiki M, Dohi K, Akiyama M. Absence of correlations between radiosensitivities of human T-lymphocytes in G0 and skin fibroblasts in log phase. Radiat Res. 1990;122(3):326–32. doi: 10.2307/3577763 2356287

[60] Teresa Pinto A, Laranjeiro Pinto M, Patrícia Cardoso A, Monteiro C, Teixeira Pinto M, Filipe Maia A, et al. Ionizing radiation modulates human macrophages towards a pro-inflammatory phenotype preserving their pro-invasive and pro-angiogenic capacities. Sci Rep. 2016;6:18765. doi: 10.1038/srep18765 26735768 PMC4702523

[61] Tabraue C, Lara PC, De Mirecki-Garrido M, De La Rosa JV, López-Blanco F, Fernández-Pérez L, et al. LXR Signaling Regulates Macrophage Survival and Inflammation in Response to Ionizing Radiation. Int J Radiat Oncol Biol Phys. 2019;104(4):913–23. doi: 10.1016/j.ijrobp.2019.03.028 30922944

[62] Glasstone S, Dolan PJ. The Effects of Nuclear Weapons. U.S. Department of Defense. 1977.

[63] Brook I, Elliott TB, Ledney GD. Infection after Ionizing Radiation. Handbook of Animal Models of Infection. Elsevier. 1999:151–61. doi: 10.1016/b978-012775390-4/50156-1

[64] Trampuz A, Piper KE, Steckelberg JM, Patel R. Effect of gamma irradiation on viability and DNA of Staphylococcus epidermidis and Escherichia coli. J Med Microbiol. 2006;55(Pt 9):1271–5. doi: 10.1099/jmm.0.46488-0 16914659

[65] Binte Atique F, Ahmed KT, Asaduzzaman SM, Hasan KN. Effects of gamma irradiation on bacterial microflora associated with human amniotic membrane. Biomed Res Int. 2013;2013:586561. doi: 10.1155/2013/586561 24063009 PMC3770025

[66] Abazari M, Ghaffari A, Rashidzadeh H, Badeleh SM, Maleki Y. A Systematic Review on Classification, Identification, and Healing Process of Burn Wound Healing. Int J Low Extrem Wounds. 2022;21(1):18–30. doi: 10.1177/1534734620924857 32524874

[67] Jeschke MG, van Baar ME, Choudhry MA, Chung KK, Gibran NS, Logsetty S. Burn injury. Nat Rev Dis Primers. 2020;6(1):11. doi: 10.1038/s41572-020-0145-5 32054846 PMC7224101

[68] Maslova E, Eisaiankhongi L, Sjöberg F, McCarthy RR. Burns and biofilms: priority pathogens and in vivo models. NPJ Biofilms Microbiomes. 2021;7(1):73. doi: 10.1038/s41522-021-00243-2 34504100 PMC8429633

[69] Dela A, Shtylla B, de Pillis L. Multi-method global sensitivity analysis of mathematical models. J Theor Biol. 2022;546:111159. doi: 10.1016/j.jtbi.2022.111159 35577102

[70] Saltelli A, Tarantola S, Campolongo F, Ratto M. Sensitivity Analysis in Practice: A Guide to Assessing Scientific Models. Wiley. 2004.

[71] Saltelli A, Tarantola S, Chan KP-S. A Quantitative Model-Independent Method for Global Sensitivity Analysis of Model Output. Technometrics. 1999;41(1):39–56. doi: 10.1080/00401706.1999.10485594

[72] Marino S, Hogue IB, Ray CJ, Kirschner DE. A methodology for performing global uncertainty and sensitivity analysis in systems biology. J Theor Biol. 2008;254(1):178–96. doi: 10.1016/j.jtbi.2008.04.011 18572196 PMC2570191

[73] Evers LH, Bhavsar D, Mailänder P. The biology of burn injury. Exp Dermatol. 2010;19(9):777–83. doi: 10.1111/j.1600-0625.2010.01105.x 20629737

[74] Peng D, Huang W, Ai S, Wang S. Clinical significance of leukocyte infiltrative response in deep wound of patients with major burns. Burns. 2006;32(8):946–50. doi: 10.1016/j.burns.2006.03.003 16901653

[75] Lateef Z, Stuart G, Jones N, Mercer A, Fleming S, Wise L. The Cutaneous Inflammatory Response to Thermal Burn Injury in a Murine Model. Int J Mol Sci. 2019;20(3):538. doi: 10.3390/ijms20030538 30696002 PMC6387172

[76] Palmer JL, Deburghgraeve CR, Bird MD, Hauer-Jensen M, Kovacs EJ. Development of a combined radiation and burn injury model. J Burn Care Res. 2011;32(2):317–23. doi: 10.1097/BCR.0b013e31820aafa9 21233728 PMC3062624

[77] Jadhav SS, Meeks CJ, Mordwinkin NM, Espinoza TB, Louie SG, diZerega GS, et al. Effect of combined radiation injury on cell death and inflammation in skin. Apoptosis. 2015;20(7):892–906. doi: 10.1007/s10495-015-1116-2 25772546

[78] Wang H, Adamzadeh M, Burgei WA, Foley SE, Zhou H. Extracting Human Reaction Time from Observations in the Method of Constant Stimuli. JAMP. 2022;10(11):3316–45. doi: 10.4236/jamp.2022.1011220

[79] Ran X, Cheng T, Shi C, Xu H, Qu J, Yan G, et al. The effects of total-body irradiation on the survival and skin wound healing of rats with combined radiation-wound injury. J Trauma. 2004;57(5):1087–93. doi: 10.1097/01.ta.0000141885.72033.c7 15580037

[80] Kiang JG, Garrison BR, Burns TM, Zhai M, Dews IC, Ney PH, et al. Wound trauma alters ionizing radiation dose assessment. Cell Biosci. 2012;2(1):20. doi: 10.1186/2045-3701-2-20 22686656 PMC3469379

[81] Murphy K, Weaver W. Janeway’s Immunobiology. Garland Science. 2017.

[82] López M, Martín M. Medical management of the acute radiation syndrome. Rep Pract Oncol Radiother. 2011;16(4):138–46. doi: 10.1016/j.rpor.2011.05.001 24376971 PMC3863169

[83] Dubin AE, Patapoutian A. Nociceptors: the sensors of the pain pathway. J Clin Invest. 2010;120(11):3760–72. doi: 10.1172/JCI42843 21041958 PMC2964977

[84] Yang T, Yang Y, Wang D, Li C, Qu Y, Guo J, et al. The clinical value of cytokines in chronic fatigue syndrome. J Transl Med. 2019;17(1):213. doi: 10.1186/s12967-019-1948-6 31253154 PMC6599310

[85] Camilleri M. LX-1031, a tryptophan 5-hydroxylase inhibitor, and its potential in chronic diarrhea associated with increased serotonin. Neurogastroenterol Motil. 2011;23(3):193–200. doi: 10.1111/j.1365-2982.2010.01643.x 21159063 PMC3076306

[86] Gale JD. Serotonergic mediation of vomiting. J Pediatr Gastroenterol Nutr. 1995;21 Suppl 1:S22–8. doi: 10.1097/00005176-199501001-00008 8708863

[87] Mawe GM, Hoffman JM. Serotonin signalling in the gut--functions, dysfunctions and therapeutic targets. Nat Rev Gastroenterol Hepatol. 2013;10(8):473–86. doi: 10.1038/nrgastro.2013.105 23797870 PMC4048923

