# Supplementary material for: Mathematical modeling of the combined effects of thermal burn and local irradiation
Source: PLoS One. 2026 Feb 10;21(2):e0341595. doi: 10.1371/journal.pone.0341595 (PMC12890176; doi:10.1371/journal.pone.0341595)
Supplement: S1 Table — Model parameters including descriptions and units. The listed numerical value was determined by the representative set. (PDF) [file pone.0341595.s001.pdf]

PARAMETER		DESCRIPTION	UNITS	VALUE
Inhibition (non-radiation related)	Deb_{dam}^{∞}	Constant controlling the effectiveness of Deb_{tb} at inhibiting damage repair	D-units	0.0100
	Deb_H	Constant controlling the effectiveness of Deb_{tb} at inhibiting phagocytic removal of debris by N_{tb}, $M1_{tb}$ and $M2_{tb}$	D-units	0.0610
	N_1^{∞}	Constant controlling the effectiveness of N_{tb} at inhibiting the activity on other immune cells	N-units	0.1692
	N_2^{∞}	Constant controlling the effectiveness of N_{tb} at inhibiting proliferation and activation of F_{tb}	N-units	0.1000
	$M1_1^{\infty}$	Constant controlling the effectiveness of $M1_{tb}$ at inhibiting activation of $M2_{tb}$	M-units	0.1552
	$M1_2^{\infty}$	Constant controlling the effectiveness of $M1_{tb}$ at inhibiting proliferation and activation of F_{tb}	M-units	0.0843
	$M2^{\infty}$	Constant controlling the effectiveness of $M2_{tb}$ at inhibiting activation of N_{tb} and $M1_{tb}$	M-units	0.5739
	F^{∞}	Constant controlling the effectiveness of F_{tb} at inhibiting the activation of $M2_{tb}$ and $L2_{tb}$	F-units	0.5314
	$L2_1^{\infty}$	Constant controlling the effectiveness of $L2_{tb}$ at inhibiting the activation of N_{tb}, $M1_{tb}$ and $L1_{tb}$	L-units	0.4738
	$L2_2^{\infty}$	Constant controlling the effectiveness of $L2_{tb}$ at inhibiting collateral damage associated with N_{tb} and $M1_{tb}$	L-units	0.4738

PARAMETER		DESCRIPTION	UNITS	VALUE
Inhibition (radiation-related)	ω_{dam}	Constant controlling the effectiveness of F_{st}^{d} at inhibiting removal of Dam_{tb} by F_{tb}	F-units	0.0200
	ω_{m2}	Constant controlling the effectiveness of $\bar{M}_{st}^{d}$ at inhibiting activation of $\bar{M}_{st}^{ud}$ and $\bar{M}_{st}^{d}$ to $M2_{tb}$	M-units	10
	ω_{f}	Constant controlling the effectiveness of F_{st}^{d} at inhibiting proliferation of F_{tb}	F-units	0.0332
	γ_{n}	Constant controlling the effectiveness of $Dose_{rad}$ at inhibiting neutrophil infiltration to the surrounding tissue	Gy/h	0.1123
	γ_{m}	Constant controlling the effectiveness of $Dose_{rad}$ at inhibiting monocyte infiltration to the surrounding tissue	Gy/h	0.1834
	γ_{l}	Constant controlling the effectiveness of $Dose_{rad}$ at inhibiting lymphocyte infiltration to the surrounding tissue	Gy/h	0.1667
	γ_{f}	Constant controlling the effectiveness of $Dose_{rad}$ at inhibiting fibroblast infiltration to the surrounding tissue	Gy/h	0.0500
Damage/Debris	k_{dn}	Maximum collateral tissue damage rate caused by N_{tb} at the local wound site	D-units/h	1.7500×10^{-4}
	k_{dm1}	Maximum collateral tissue damage rate caused by $M1_{tb}$ at the local wound site	D-units/h	$\sigma_{dm1} \cdot k_{dn}$
	σ_{dm1}	Tissue damage scaling coefficient for $M1_{tb}$	Unitless	0.5000
	N_{H}	Hill constant for tissue damage associated with N_{tb}	N-units	0.0600
	$M1_{H}$	Hill constant for tissue damage associated with $M1_{tb}$	M-units	0.0600
	ρ_{dam}	Baseline tissue damage repair rate via a combination of repair, resolution, and regeneration	1/h	0.0040

PARAMETER		DESCRIPTION	UNITS	VALUE
	k_{df}	Repair rate on Dam_{tb} by F_{tb}	1/(F-units·h)	0.0200
	k_{dnp}	Phagocytosis rate of Deb_{tb} by N_{tb}	D-units/(N-units·h)	0.0167
	k_{dm1p}	Phagocytosis rate of Deb_{tb} by $M1_{tb}$	D-units/(M-units·h)	0.0327
	k_{dm2p}	Phagocytosis rate of Deb_{tb} by $M2_{tb}$	D-units/(M-units·h)	0.0101
	d_{deb}	Decay rate of Deb_{tb}	1/h	0.0001
Neutrophils	s_{nr}	Recruitment rate of circulating neutrophils to the local wound site	N-units/h	0.0133
	d_{nr}	Decay rate of $\bar{N}_{st}^{ud}$	1/h	0.0190
	d_n	Decay rate of N_{tb}	1/h	0.0291
	k_{nd}	Activation rate of resting neutrophils $\bar{N}_{st}^{ud}$ to N_{tb} by Deb_{tb}	1/(D-units·h)	0.5820
	k_{np}	Activation rate of resting neutrophils $\bar{N}_{st}^{ud}$ to N_{tb} by P_{tb}	1/(P-units·h)	7.325
	k_{nn}	Activation rate of resting neutrophils $\bar{N}_{st}^{ud}$ to N_{tb} by N_{tb} and its byproducts	1/(N-units·h)	0.0169
	k_{nm1p}	Phagocytosis rate of N_{tb} by $M1_{tb}$	1/(M-units·h)	0.0573
	k_{nm2p}	Phagocytosis rate of N_{tb} by $M2_{tb}$	1/(M-units·h)	0.3628
Macrophages	s_{mr}	Recruitment rate of fixed tissue and circulating monocytes to the local wound site	M-units/h	1.0247
	d_{mr}	Decay rate of $\bar{M}_{st}^{ud}$	1/h	0.0151
	d_{mr}^{d}	Decay rate of $\bar{M}_{st}^{d}$	1/h	0.0050
	d_{m1}	Decay rate of $M1_{tb}$	1/h	0.1033

PARAMETER		DESCRIPTION	UNITS	VALUE
	d_{m2}	Decay rate of $M2_{tb}$	1/h	0.1236
	k_{m1d}	Activation rate of resting monocytes $\bar{M}_{st}^{ud}$ and $\bar{M}_{st}^{d}$ to $M1_{tb}$ by Deb_{tb}	1/(D-units·h)	0.0021
	k_{m1p}	Activation rate of resting monocytes $\bar{M}_{st}^{ud}$ and $\bar{M}_{st}^{d}$ to $M1_{tb}$ by P_{tb}	1/(P-units·h)	2.9303×10^{-4}
	k_{m1n}	Activation rate of resting monocytes $\bar{M}_{st}^{ud}$ and $\bar{M}_{st}^{d}$ to $M1_{tb}$ by N_{tb} and its byproducts	1/(N-units·h)	0.0353
	k_{m1m1}	Activation rate of resting monocytes $\bar{M}_{st}^{ud}$ and $\bar{M}_{st}^{d}$ to $M1_{tb}$ by $M1_{tb}$ and its byproducts	1/(M-units·h)	3.7879×10^{-5}
	k_{m1l1}	Activation rate of resting monocytes $\bar{M}_{st}^{ud}$ and $\bar{M}_{st}^{d}$ to $M1_{tb}$ by $L1_{tb}$ and its byproducts	1/(L-units·h)	1.5542×10^{-4}
	k_{m2m1}	Activation rate of resting monocytes $\bar{M}_{st}^{ud}$ and $\bar{M}_{st}^{d}$ to $M2_{tb}$ by $M1_{tb}$ and its byproducts	1/(M-units·h)	0.0051
	k_{m2m2}	Activation rate of resting monocytes $\bar{M}_{st}^{ud}$ and $\bar{M}_{st}^{d}$ to $M2_{tb}$ by $M2_{tb}$ and its byproducts	1/(M-units·h)	6.7667×10^{-4}
	k_{m2l2}	Activation rate of resting monocytes $\bar{M}_{st}^{ud}$ and $\bar{M}_{st}^{d}$ to $M2_{tb}$ by $L2_{tb}$ and its byproducts	1/(L-units·h)	0.0049
	θ_{m1m2}	Transition rate of $M1_{tb}$ to $M2_{tb}$	M-units/N-units	0.6901
Fibroblasts	s_f	Non-injury recruitment rate of fibroblasts to the local wound site	F-units/h	0.0014
	k_{ftb}^{ud}	Rate of transition of undamaged fibroblasts $\left(F_{st}^{ud}\right)$ from the surrounding tissue to the thermal burn	1/h	0.0042
	k_{ftb}^{d}	Rate of transition of damaged fibroblasts $\left(F_{st}^{ud}\right)$ from the surrounding tissue to the thermal burn	1/h	0.0012

PARAMETER		DESCRIPTION	UNITS	VALUE
	d_{fr}^{ud}	Decay rate of F_{st}^{ud}	1/h	0.0091
	d_{fr}^{d}	Decay rate of F_{st}^{d}	1/h	9.0909×10^{-4}
	d_f	Decay rate of F_{tb}	1/h	0.0091
	k_f	Baseline proliferation and activation rate of F_{tb}	1/h	0.0053
	α_{m2}	Constant controlling the up-regulation of F_{tb} proliferation and activation by $M2_{tb}$	1/(M-units·h)	0.0455
	α_{dam}	Constant controlling the up-regulation of F_{tb} proliferation and activation by Dam_{tb}	1/(D-units·h)	0.0667
T Lymphocytes	$s_{\ell r}$	Recruitment rate of circulating T lymphocytes to the local wound site	L-units/h	0.0283
	$d_{\ell r}^{ud}$	Decay rate of L_{st}^{ud}	1/h	0.0565
	$d_{\ell r}^{d}$	Decay rate of L_{st}^{d}	1/h	0.0283
	k_{l1}	Induction rate of L_{st}^{ud} and L_{st}^{d} to $L1_{tb}$ by $M1_{tb}$	1/(M-units·h)	0.0698
	k_{l2}	Induction rate of L_{st}^{ud} and L_{st}^{d} to $L2_{tb}$ by $M2_{tb}$	1/(M-units·h)	0.0369
	d_ℓ	Decay rate of $L1_{tb}$ and $L2_{tb}$	1/h	0.0594
Pathogen	k_{pg}	Growth rate of P_{tb}	1/h	0.4681
	P^∞	Carrying capacity of P_{tb}	P-units	32.7541
	k_{pb}	Phagocytosis rate of P_{tb} by the background immune response	1/(B-units·h)	1.1047
	s_b	Recruitment rate of circulating background immune cells	B-units/h	0.0082

PARAMETER		DESCRIPTION	UNITS	VALUE
	μ_b	Decay rate of background immune cells	1/h	0.0020
	k_{bp}	Phagocytosis rate of cells in the background immune response by P_{tb}	1/(M-units·h)	0.0083
	k_{pn}	Phagocytosis rate of P_{tb} by N_{tb}	N-units/h	2.9755
	k_{pm1}	Phagocytosis rate of P_{tb} by $M1_{tb}$	M-units/h	0.0947
	k_{pm2}	Phagocytosis rate of P_{tb} by $M2_{tb}$	M-units/h	0.0947

Notation:

- Dam_{tb} and Deb_{tb} have the same units- D is used to denote their units.
- N, M, F, L, and P denote the units of neutrophils (N_{st}^{ud}, N_{st}^{d}, and N_{tb}), macrophages ($\bar{M}_{st}^{ud}$, $\bar{M}_{st}^{d}$, $M1_{tb}$, and $M2_{tb}$), fibroblasts (F_{st}^{ud}, F_{st}^{d}, and F_{tb}), T lymphocytes (L_{st}^{ud}, L_{st}^{d}, $L1_{tb}$, and $L2_{tb}$), and pathogen (P_{tb}) respectively.
- B denotes the units for the background immune response including mast cells and natural killer cells.
- Gy denotes the units for radiation dose (Gray).
