# Supplementary material for: Mathematical modeling of the combined effects of thermal burn and local irradiation
Source: PLoS One. 2026 Feb 10;21(2):e0341595. doi: 10.1371/journal.pone.0341595 (PMC12890176; doi:10.1371/journal.pone.0341595)
Supplement: S2 File — (PDF) [file pone.0341595.s002.pdf]

# S2 File. Detailed description of model equations.

## Damaged tissue and debris

The equations for the rate of change in damage and debris at the thermal burn injury site are given by the following equations.

$$\frac{dDam_{tb}}{dt} = \underbrace{k_{dn}\Omega_H(\Omega_i(N_{tb}; L2_{tb}, L2_2^{\infty}), 6, N_H)}_{\text{Collateral damage from activated neutrophils}} + \underbrace{k_{dm1}\Omega_H(\Omega_i(M1_{tb}; L2_{tb}, L2_2^{\infty}), 6, M1_H)}_{\text{Collateral damage from activated M1 macrophages}}$$
$$- \underbrace{\rho_{dam}\Omega_i(Dam_{tb}; Deb_{tb}, Deb_{dam}^{\infty})}_{\text{Background resolution of damage}} \qquad (1)$$
$$- \underbrace{\Omega_i\left(F_{tb}; F_{st}^{d}, \omega_{dam}\right)k_{df}\Omega_i(Dam_{tb}; Deb_{tb}, Deb_{dam}^{\infty})}_{\text{Resolution of damage by proliferating fibroblasts (inhibited by radiation)}}$$

$$\frac{dDeb_{tb}}{dt} = \underbrace{k_{dn}\Omega_H(\Omega_i(N_{tb}; L2_{tb}, L2_2^{\infty}), 6, N_H)}_{\text{Debris generated by activated neutrophils}} + \underbrace{k_{dm1}\Omega_H(\Omega_i(M1_{tb}; L2_{tb}, L2_2^{\infty}), 6, M1_H)}_{\text{Debris generated by activated M1 macrophages}}$$
$$- \underbrace{k_{dnp}N_{tb}\Omega_H(Deb_{tb}, 1, Deb_H)}_{\text{Phagocytosis of debris by activated neutrophils}} - \underbrace{k_{dm1p}\Omega_i(M1_{tb}; N_{tb}, N_1^{\infty})\Omega_H(Deb_{tb}, 1, Deb_H)}_{\text{Phagocytosis of debris by activated M1 macrophages}} \qquad (2)$$
$$- \underbrace{k_{dm2p}\Omega_i(M2_{tb}; N_{tb}, N_1^{\infty})\Omega_H(Deb_{tb}, 1, Deb_H)}_{\text{Phagocytosis of debris by activated M2 macrophages}} - \underbrace{d_{deb}Deb_{tb}}_{\text{Background removal and decay}}$$

Damaged tissue and cellular debris result from direct damage to cells from thermal fluence as well as collateral damage due to proteases and other tissue degrading molecules produced by neutrophils and M1 macrophages during the inflammatory phase [1–3]. This process results in further damage and generation of cellular debris during the wound healing process. The initial extent of injury caused by the thermal fluence is characterized by the initial conditions of these two variables. Additional damage and debris caused by the inflammatory cells are described by the first two terms in Equations 1 and 2. This process is modeled using a saturating hill function, which has been employed in other wound healing models [4,5]. Here we used an exponent value of 6 which creates a steep increasing function such that low levels of inflammatory cells result in little to no additional damage and higher levels (as observed during the inflammatory phase) result in additional collateral damage.

Damage in the thermal injury site is resolved through a fibroblast independent term and a fibroblast dependent term. These resolution pathways are not modeled explicitly and are instead accounted for with representative expressions in the third and fourth terms, respectively, in Equation 1. Although it is not explicitly modeled, damage resolution by activated fibroblasts captures early repair processes indicated by collagen deposition at the wound site. Collagen has been observed to be a good marker of tissue damage resolution in other mathematical models of

wound healing [6]. This equation term also includes an inhibitory function dependent on the population of damaged fibroblasts in the surrounding tissue. Radiation has been evidenced to inhibit the deposition of collagen by fibroblasts [7–9]. Early in the healing process, we assume that some damaged fibroblasts from the surrounding tissue migrate to the wound and perform deficiently, accounted for by the inhibitory multiplier which scales with the population of damaged fibroblasts.

Debris is removed through phagocytosis by inflammatory cells (neutrophils and macrophages). This is directly modeled by terms three through five in Equation 2. Debris is also removed by additional inflammatory processes not specifically included in the model variables. This is accounted for by the final term in Equation 2.

## Neutrophils

The differential equations for the rate of change in the neutrophil populations are represented by the following equations in both the thermal burn and surrounding tissue compartments.

$$\frac{d\bar{N}_{st}^{ud}}{dt} = \underbrace{v(t, Dose_{rad}; \gamma_n) s_{nr}}_{\text{Neutrophil recruitment from the bloodstream}} - \underbrace{R_n \bar{N}_{st}^{ud}}_{\text{Neutrophil activation}} - \underbrace{d_{nr} \bar{N}_{st}^{ud}}_{\text{Decay}} \tag{3}$$

$$\begin{aligned} \frac{dN_{tb}}{dt} = \frac{dN_{tb}}{dt} = & \underbrace{R_n \bar{N}_{st}^{ud}}_{\text{Neutrophil activation at the injury site}} - \underbrace{k_{nm1p} N_{tb} \Omega_i(M1_{tb}; N_{tb}, N_1^{\infty})}_{\text{Phagocytosis of neutrophils by M1 macrophages}} \\ & - \underbrace{k_{nm2p} N_{tb} \Omega_i(M2_{tb}; N_{tb}, N_1^{\infty})}_{\text{Phagocytosis of neutrophils by M2 macrophages}} - \underbrace{d_n N_{tb}}_{Decay} \end{aligned} \tag{4}$$

$$R_n = \Omega_i(\underbrace{k_{nd} Deb_{tb} + k_{np} P_{tb} + k_{nn} N_{tb}}_{\text{Activation of neutrophils by debris signals, pathogen, and other neutrophils}}; \underbrace{M2_{tb}, M2^{\infty}, L2_{tb}, L2_1^{\infty}}_{\text{Inhibition from M2 macrophages and L2 lymphocytes}} \tag{5}$$

Neutrophils in the surrounding tissue are recruited from the blood stream at a baseline constant rate, $s_{nr}$, represented in the first term of Equation 3. Localized ionizing radiation exposure damages the microvasculature [7,10] resulting in delayed infiltration of recruited neutrophils and other immune cells [11]. Because the process of revascularization is not modeled explicitly, this delay is represented by an inhibitory function that decays over time, and modifies the baseline influx rate of the first term of Equation 3. The inhibition function is dose dependent, ensuring that large radiation doses result in increased vascular damage to the surrounding tissue and prolonged inhibition of immune cell influx.

Neutrophils are then activated and recruited to the wound site from the surrounding tissue by signaling biomolecules, including cytokines and chemokines, produced by debris, foreign

infiltrates, and other neutrophils in the wound. The activation rate is inhibited by the anti-inflammatory immune cells (M2 macrophages and L2 lymphocytes) through anti-inflammatory signals. The rate of activation is described by Equation 5 and is represented in the second term of Equation 3 and the first term of Equation 4. Neutrophils in the surrounding tissue also experience intrinsic decay represented by the last term in Equation 3.

Neutrophils at the thermal injury site undergo apoptosis after performing phagocytosis of debris and are later phagocytized by activated M1 and M2 macrophages at the wound site. This is represented by the second and third terms in Equation 4. Neutrophils may also be removed by other processes which are collectively represented by the final term in Equation 4.

## Monocytes and macrophages

The equations for the rate of change of inactivated monocytes and activated macrophages are represented by Equations 6-11 in the thermal burn and surrounding tissue compartments.

$$\frac{d\bar{M}_{st}^{ud}}{dt} = \underbrace{v(t, D_R; \gamma_m) s_{mr}}_{\substack{\text{Macrophage recruitment} \\ \text{from the bloodstream} \\ \text{(inhibited by radiation)}}} - \underbrace{R_{m1}\bar{M}_{st}^{ud}}_{\substack{\text{Activation to the} \\ \text{M1 phenotype}}} - \underbrace{\Omega_i\left(R_{m2}; \bar{M}_{st}^{d}, \omega_{m2}\right)\bar{M}_{st}^{ud}}_{\substack{\text{Activation to the M2 phenotype} \\ \text{(inhibited by radiation)}}} - \underbrace{d_{mr}^{ud}\bar{M}_{st}^{ud}}_{\text{Decay}} \tag{6}$$

$$\frac{d\bar{M}_{st}^{d}}{dt} = \underbrace{-R_{m1}\bar{M}_{st}^{d}}_{\substack{\text{Activation to the} \\ \text{M1 phenotype}}} - \underbrace{\Omega_i\left(R_{m2}; \bar{M}_{st}^{d}, \omega_{m2}\right)\bar{M}_{st}^{d}}_{\substack{\text{Activation to the M2 phenotype} \\ \text{(inhibited by radiation)}}} - \underbrace{d_{mr}^{d}\bar{M}_{st}^{d}}_{\text{Decay}} \tag{7}$$

$$\frac{dM1_{tb}}{dt} = \underbrace{R_{m1}\left(\bar{M}_{st}^{ud} + \bar{M}_{st}^{d}\right)}_{\substack{\text{Macrophage activation} \\ \text{at the injury site}}} - \underbrace{\theta_{m1m2}\left[k_{nm1p}N_{tb}\Omega_i(M1_{tb}; N_{tb}, N_1^{\infty})\right]}_{\substack{\text{Transition of M1 macrophages to} \\ \text{M2 macrophages}}} - \underbrace{d_{m1}M1_{tb}}_{\text{Decay}} \tag{8}$$

$$\frac{dM2_{tb}}{dt} = \underbrace{\Omega_i\left(R_{m2}; \bar{M}_{st}^{d}, \omega_{m2}\right)\left(\bar{M}_{st}^{ud} + \bar{M}_{st}^{d}\right)}_{\substack{\text{Macrophage activation at the injury} \\ \text{site (inhibited by radiation)}}} + \underbrace{\theta_{m1m2}\left[k_{nm1p}N_{tb}\Omega_i(M1_{tb}; N_{tb}, N_1^{\infty})\right]}_{\substack{\text{Transition of M1 macrophages to} \\ \text{M2 macrophages}}} - \underbrace{d_{m2}M2_{tb}}_{\text{Decay}} \tag{9}$$

$$R_{m1} = \Omega_i(\underbrace{k_{m1d}Deb_{tb} + k_{m1p}P_{tb} + k_{m1n}N_{tb} + k_{m1m1}M1_{tb}}_{\substack{\text{Activation by debris, pathogen, neutrophils} \\ \text{and macrophages}}} + \underbrace{k_{m1l1}L1_{tb}}_{\substack{\text{Activation by} \\ \text{lymphocytes}}};\ \underbrace{M2_{tb}, M2^{\infty}, L2_{tb}, L2_1^{\infty}}_{\substack{\text{Inhibition by M2 macrophages} \\ \text{and L2 lymphocytes}}}) \tag{10}$$

$$R_{m2} = \Omega_i(\underbrace{k_{m2m1}M1_{tb} + k_{m2m2}M2_{tb} + k_{m2l2}L2_{tb}}_{\text{Activation by macrophages and lymphocytes}}\,;\underbrace{N_{tb}, N_1^{\infty}, M1_{tb}, M1_1^{\infty}, F_{tb}, F^{\infty}}_{\substack{\text{Inhibition by neutrophils,}\\ \text{macrophages, and}\\ \text{fibroblasts}}}) \tag{11}$$

Monocytes in the surrounding tissue have two states: undamaged or damaged, denoted by a $ud$ or $d$ superscript, respectively. Undamaged monocytes in the surrounding tissue are recruited from the blood stream at a baseline constant rate, represented by the parameter $s_{mr}$ in the first term of Equation 6. Similar to the equation defined for neutrophils in the surrounding tissue (Equation 3), this baseline rate is inhibited when radiation injury occurs in a dose-dependent manner.

Undamaged monocytes can then be recruited to the thermal burn compartment and activated towards the M1 classically activated phenotype or the M2 alternatively activated phenotype represented by the second and third terms in Equation 6, respectively. Activated macrophages can display a spectrum of pro- to anti-inflammatory behavior [12], but for simplicity, we assume that all macrophages will be either exclusively pro-inflammatory (M1) or anti-inflammatory (M2). The M1 pro-inflammatory phenotype is involved in phagocytosis of debris and apoptotic neutrophils [12]. Activation towards this phenotype is stimulated by signaling biomolecules associated with the presence of debris, foreign pathogen, neutrophils, other M1 macrophages, and L1 lymphocytes. Activation is inhibited by the presence of M2 macrophages and L2 lymphocytes. Details regarding activation towards this phenotype are in Equation 10. The M2 anti-inflammatory phenotype is involved in phagocytosis of any debris generated from neutrophils and M1 macrophages and down-regulation of the pro-inflammatory response. Activation toward the M2 phenotype is stimulated by M1 macrophages and other M2 macrophages. Activation is inhibited by neutrophils and M1 macrophages due to their pro-inflammatory signaling and the presence of fibroblasts during the proliferation phase. This is represented in Equation 11.

Damaged monocytes in the surrounding tissue inhibit the activation of monocytes towards the M2 phenotype. For prompt gamma radiation doses above 2 Gray (Gy), macrophages are polarized towards the M1 phenotype [13]. Current research involving radiation-induced macrophage polarization has not described the exact mechanisms by which this switch in activation occurs. Given the prolific evidence of increased pro-inflammatory cytokines following irradiation [14–16], it is reasonable to assume that this overexpression of pro-inflammatory cytokines could be the mechanism by which activation towards the M1 phenotype occurs. For this reason, the alterative activation is dependent upon the variable associated with damaged macrophage cells in the surrounding tissue which includes all associated cytokines, chemokines, and other signals. This allows for both a time- and dose-dependent inhibition of the activation process toward the M2 phenotype. This mechanism is represented in the first term of Equation 9. Consistent with current research [13], the activation towards the M2 phenotype is only inhibited for $D_R \geq 2$ Gy, otherwise, activation occurs at the rate $R_{m2}$. Undamaged monocytes can also simply decay in the surrounding tissue, represented by the last term in Equation 6.

Damaged monocytes represent an instantaneous damage state initiated by a prompt radiation dose. The radiation induces DNA damage causing cells to perform abnormally and initiate an early death. Damaged monocytes can still be activated towards the M1 or M2 phenotypes and we assume that this rate is the same as for undamaged monocytes. Damaged monocytes can also decay in the surrounding tissue, and we assume that this rate is higher than for undamaged monocytes.

Equations 8 and 9 represent the activated form of macrophages represented as M1 or M2 macrophages in the thermal burn compartment. The first term in each equation represents the activation terms matching those in the monocyte equations in the surrounding tissue. The second term in each equation represents the switch of M1 activated macrophages to M2 activated macrophages used in previous mathematical models involving macrophage activation [17]. The switch in macrophage phenotypes is typically the result of M1 phagocytosis of apoptotic neutrophils [12], which are not explicitly modeled, so instead we model this rate using an interaction term between neutrophil and M1 macrophage populations. This mechanism is also inhibited by the presence of neutrophils since their pro-inflammatory signals will promote the M1 phenotype and inhibit activation towards the M2 phenotype. The final term in each equation represents intrinsic decay.

## T lymphocytes

The equations for the rate of change of inactivated lymphocytes and activated lymphocytes are represented by the following equations in the thermal burn and surrounding tissue compartments.

$$\frac{dL_{st}^{ud}}{dt} = \underbrace{v(t, Dose_{rad}; \gamma_l) s_{lr}}_{\substack{\text{Lymphocyte recruitment} \\ \text{from the bloodstream} \\ \text{(inhibited by radiation)}}} - \underbrace{R_{l1} L_{st}^{ud}}_{\substack{\text{Activation to the} \\ \text{L1 phenotype}}} - \underbrace{R_{l2} L_{st}^{ud}}_{\substack{\text{Activation to the} \\ \text{L2 phenotype}}} - \underbrace{d_{lr}^{ud} L_{st}^{ud}}_{\text{Decay}} \tag{12}$$

$$\frac{dL_{st}^{d}}{dt} = \underbrace{-R_{l1} L_{st}^{d}}_{\substack{\text{Activation to the} \\ \text{L1 phenotype}}} - \underbrace{R_{l2} L_{st}^{d}}_{\substack{\text{Activation to the} \\ \text{L2 phenotype}}} - \underbrace{d_{lr}^{d} L_{st}^{d}}_{\text{Decay}} \tag{13}$$

$$\frac{dL1_{tb}}{dt} = \underbrace{R_{l1}\left(L_{st}^{ud} + L_{st}^{d}\right)}_{\substack{\text{Activation to the} \\ \text{L1 phenotype}}} - \underbrace{d_l L1_{tb}}_{\text{Decay}} \tag{14}$$

$$\frac{dL2_{tb}}{dt} = \underbrace{R_{l2}\left(L_{st}^{ud} + L_{st}^{d}\right)}_{\substack{\text{Activation to the} \\ \text{L2 phenotype}}} - \underbrace{d_l L2_{tb}}_{\text{Decay}} \tag{15}$$

$$R_{l1} = \underbrace{\Omega_i(k_{l1}M1_{tb}; L2_{tb}, L2_1^{\infty})}_{\substack{\text{Activation by M1 macrophages} \\ \text{with inhibition by L2 lymphocytes}}} \tag{16}$$

$$R_{l2} = \underbrace{\Omega_i(k_{l2}M2_{tb}; F_{tb}, F^{\infty})}_{\substack{\text{Activation by M2 macrophages} \\ \text{with inhibition by fibroblasts}}} \tag{17}$$

Only T-cell lymphocytes are considered in the present model which include the pro-inflammatory phenotypes (e.g. γδ T cells, TH1 cells, and T17 cells) and anti-inflammatory phenotypes (e.g. TH2 cells and regulatory T cells). Lymphocyte cells are highly radiosensitive [18,19] and have tissue resident populations in the surrounding tissue, thus we include an undamaged and damaged population indicated by $ud$ or $d$ in the superscript, respectively. Lymphocyte function in the present model consists of general regulation of the macrophage response through inflammatory mediators and other signaling biomolecules. At present, damaged lymphocytes in the surrounding tissue do not affect lymphocyte function or polarization, as was seen in the macrophage function, but the population of damaged lymphocytes is used to determine the occurrence of observable medical outcomes.

Undamaged lymphocytes are recruited from the blood vessels at a baseline constant rate represented by the parameter $s_{lr}$ in Equation 12. This rate is inhibited by the same mechanism presented for neutrophils and macrophages reflected in the first term. Lymphocytes can then be activated to either the L1 classically activated or L2 alternatively activated lymphocytes, represented by the second and third terms in Equation 12 and the first and second terms in Equation 13. L1 lymphocytes are activated by the presence of M1 macrophages and inhibited by the presence of L2 lymphocytes (shown in Equation 16). L2 lymphocytes are activated by the presence of M2 macrophages and inhibited by the presence of fibroblasts which signify the transition to the proliferation phase of wound healing (shown in Equation 17). The final term in Equations 12 and 13 model intrinsic decay in the surrounding tissue where those in the damaged population have a larger decay rate than those in the undamaged population. Lymphocytes in the thermal burn can also decay at a rate $d_l$, represented in the last term of Equations 14 and 15.

## Fibroblasts

The equations for the rate of change of tissue resident fibroblasts and activated fibroblasts are represented by the following equations in the thermal burn and surrounding tissue compartments.

$$\frac{dF_{st}^{ud}}{dt} = \underbrace{v\big(t, D_R; \gamma_f\big)\, s_f}_{\substack{\text{Fibroblast recruitment} \\ \text{from the connective} \\ \text{tissues} \\ \text{(inhibited by radiation)}}} - \underbrace{k_{sttb}^{ud} F_{st}^{ud}}_{\substack{\text{Recruitment} \\ \text{of fibroblasts} \\ \text{to the injury site}}} - \underbrace{d_{fr}^{ud} F_{st}^{ud}}_{\text{Decay}} \tag{18}$$

$$\frac{dF_{st}^d}{dt} = \underbrace{-k_{sttb}^d F_{st}^d}_{\substack{\text{Recruitment}\\ \text{of fibroblasts}\\ \text{to the injury site}}} - \underbrace{d_{fr}^d F_{st}^d}_{\text{Decay}} \tag{19}$$

$$\frac{dF_{tb}}{dt} = \underbrace{\Omega_i\left(1; F_{st}^d, \omega_f\right)\Omega_i\left(F_{tb}; N_{tb}, N_2^\infty, M1_{tb}, M1_2^\infty\right)\left(k_f + \alpha_{dam} Dam_{tb} + \alpha_{m2} M2_{tb}\right)}_{\substack{\text{Proliferation of fibroblasts by other fibroblasts, damaged tissue, and M2 macrophages}\\ \text{(inhibited by inflammatory cells and radiation)}}}$$
$$+ \underbrace{k_{sttb}^{ud} F_{st}^{ud} + k_{sttb}^d F_{st}^d}_{\substack{\text{Recruitment of fibroblasts}\\ \text{to the injury site}}} - \underbrace{d_f F_{tb}}_{\text{Decay}} \tag{20}$$

Fibroblasts are known to have a resident population in the surrounding tissue, so they are represented by an undamaged and damaged population in the tissue. Undamaged fibroblasts in the surrounding tissue are recruited from the local connective tissues as well as through proliferation of resident populations at a baseline constant rate $s_f$. Again, there is inhibition of this rate dependent on the radiation dose which represents vascular damage associated with local tissue injury from radiation, represented by the first term in Equation 18. Fibroblasts can then be recruited to the thermal burn compartment, represented by the second term in Equation 18, or decay, represented by the last term. Damaged fibroblasts in the surrounding tissue can either be recruited to the thermal burn compartment, represented by the first term in Equation 19, or decay, represented by the last term.

Fibroblasts in the thermal burn compartment are recruited from the surrounding tissue, represented by the first and second term in Equation 20. Fibroblasts in the thermal burn compartment also proliferate in response to a number of mechanisms. This is represented by a background constant rate, $k_f$, proliferation caused by a lack of contact inhibition brought on by the presence of damaged tissue, $\alpha_{dam}$, and from anti-inflammatory signaling from M2 macrophages, $\alpha_{m2}$. Proliferation is inhibited by the presence of neutrophils and M1 macrophages, as these cells signal pro-inflammatory behavior. Fibroblast proliferation is further inhibited by radiation injury as this has been observed to reduce proliferative abilities [20,21]. Similar to previous equations, we use the population of damaged fibroblasts to drive this inhibition. The final term in Equation 20 represents intrinsic decay at the thermal burn site.

As shown with damaged T lymphocytes in the surrounding tissue compartment, Equation 19 also has an analytical solution solved using separation of variables and simple integration. Additionally, since this equation only has constant parameter rates, the final solution is only dependent on time. The solution is represented in Equation 21.

$$F_{st}^d(t) = F_{st,0}^d e^{-\left(k_{sttb}^d + d_{fr}^d\right)t} \tag{21}$$

## Pathogen

The equation for the rate of change of foreign pathogen is represented by the following equation in the thermal burn compartment.

$$\frac{dP_{tb}}{dt} = \underbrace{k_{pg}P_{tb}\left(1-\frac{P_{tb}}{P^{\infty}}\right)}_{\substack{\text{Logistic growth of}\\ \text{pathogen}}} - \underbrace{\frac{k_{pb}s_bP_{tb}}{\mu_b + k_{bp}P_{tb}}}_{\substack{\text{Phagocytosis by}\\ \text{background}\\ \text{inflammatory}\\ \text{response}}} - \underbrace{k_{pn}P_{tb}N_{tb}}_{\substack{\text{Phagocytosis}\\ \text{by neutrophils}}} - \underbrace{k_{pm1}P_{tb}\Omega_i(M1_{tb};N_{tb},N_1^{\infty})}_{\substack{\text{Phagocytosis by M1}\\ \text{macrophages}\\ \text{(inhibited by neutrophils)}}} - \underbrace{k_{pm2}P_{tb}\Omega_i(M2_{tb};N_{tb},N_1^{\infty})}_{\substack{\text{Phagocytosis by M2}\\ \text{macrophages}\\ \text{(inhibited by neutrophils)}}} \tag{22}$$

Ionizing radiation is associated with an increased risk of infection regardless of a localized [22] or whole-body exposure [23–26]. This risk is observed to be further elevated for irradiated burns [27]. Following the approach of other wound healing models [5,17,28], this ODE accounts for logistic pathogen growth and removal via phagocytosis by neutrophils and macrophages. The first term in Equation 22 represents growth of a pathogen with a carrying capacity $P^{\infty}$ and growth rate $k_{pg}$. Logistic growth is a widely accepted model for describing exponential growth with competition for resources [29,30]. The second term describes the removal of pathogen by the local background immune response which includes mast cells and natural killer cells whose populations are not explicitly modeled [31]. The derivation of this term, which is based on a quasi-steady state assumption, has been described in previous mathematical models [4]. The final three terms account for removal of the pathogen by neutrophils and macrophages. Neutrophil activity contributes to an oxidative environment that inhibits phagocytosis by macrophages shown in the fourth and fifth terms.

References


1. Schultz GS, Chin GA, Moldawer L, Diegelmann RF. Principles of Wound Healing. Mechanisms of Vascular Disease: A Reference Book for Vascular Specialists. University of Adelaide Press; 2011. Available: https://www.ncbi.nlm.nih.gov/books/NBK534261/

2. Namas RA, Mi Q, Namas R, Almahmoud K, Zaaqoq AM, Abdul-Malak O, et al. Insights into the Role of Chemokines, Damage-Associated Molecular Patterns, and Lymphocyte-Derived Mediators from Computational Models of Trauma-Induced Inflammation. Antioxid Redox Signal. 2015;23: 1370–1387. doi:10.1089/ars.2015.6398

3. Diegelmann R, Evans M. Wound healing: An overview of acute, fibrotic, and delayed healing. 2004; 283–289.

4. Reynolds A, Rubin J, Clermont G, Day J, Vodovotz Y, Bard Ermentrout G. A reduced mathematical model of the acute inflammatory response: I. Derivation of model and

analysis of anti-inflammation. Journal of Theoretical Biology. 2006;242: 220–236. doi:10.1016/j.jtbi.2006.02.016

5. Menke NB, Cain JW, Reynolds A, Chan DM, Segal RA, Witten TM, et al. An in silico approach to the analysis of acute wound healing. Wound Repair and Regeneration. 2010;18: 105–113. doi:10.1111/j.1524-475X.2009.00549.x

6. Segal RA, Diegelmann RF, Ward KR, Reynolds A. A Differential Equation Model of Collagen Accumulation in a Healing Wound. Bull Math Biol. 2012;74: 2165–2182. doi:10.1007/s11538-012-9751-z

7. Vegesna V, Withers RH, Holly EF, McBride WH. The Effect of Local and Systemic Irradiation on Impairment of Wound Healing in Mice. Radiation Research. 1993;135: 431–433.

8. Dormand E-L, Banwell PE, Goodacre TE. Radiotherapy and wound healing. International Wound Journal. 2005;2: 112–127. doi:10.1111/j.1742-4801.2005.00079.x

9. Liu X, Liu J-Z, Zhang E, Li P, Zhou P, Cheng T-M, et al. Impaired Wound Healing after Local Soft X-Ray Irradiation in Rat Skin: Time Course Study of Pathology, Proliferation, Cell Cycle, and Apoptosis. Journal of Trauma and Acute Care Surgery. 2005;59: 682. doi:10.1097/01.ta.0000177674.55388.40

10. Iddins CJ, DiCarlo AL, Ervin MD, Herrera-Reyes E, Goans RE. Cutaneous and Local Radiation Injuries. J Radiol Prot. 2022;42: 10.1088/1361-6498/ac241a. doi:10.1088/1361-6498/ac241a

11. Sweeney JF, Nguyen PK, Omann GM, Hinshaw DB. Ultraviolet irradiation accelerates apoptosis in human polymorphonuclear leukocytes: protection by LPS and GM-CSF. Journal of Leukocyte Biology. 1997;62: 517–523. doi:10.1002/jlb.62.4.517

12. Mosser DM, Edwards JP. Exploring the full spectrum of macrophage activation. Nat Rev Immunol. 2008;8: 958–969. doi:10.1038/nri2448

13. Rödel F, Frey B, Multhoff G, Gaipl U. Contribution of the immune system to bystander and non-targeted effects of ionizing radiation. Cancer Letters. 2015;356: 105–113. doi:10.1016/j.canlet.2013.09.015

14. Schäffer M, Weimer W, Wider S, Stülten C, Bongartz M, Budach W, et al. Differential Expression of Inflammatory Mediators in Radiation-Impaired Wound Healing. Journal of Surgical Research. 2002;107: 93–100. doi:10.1006/jsre.2002.6494

15. Kiang JG, Garrison BR, Gorbunov NV. Radiation Combined Injury: DNA Damage, Apoptosis, and Autophagy. Adapt Med. 2010;2: 1–10. doi:10.4247/AM.2010.ABA004

16. DiCarlo AL, Bandremer AC, Hollingsworth BA, Kasim S, Laniyonu A, Todd NF, et al. Cutaneous Radiation Injuries: Models, Assessment and Treatments. Radiation Research. 2020;194: 315–344. doi:10.1667/RADE-20-00120.1

17. Torres M, Wang J, Yannie PJ, Ghosh S, Segal RA, Reynolds AM. Identifying important parameters in the inflammatory process with a mathematical model of immune cell influx and macrophage polarization. PLOS Computational Biology. 2019;15: e1007172. doi:10.1371/journal.pcbi.1007172

18. United Nations Scientific Committee on the Effects of Atomic Radiation. Ionizing Radiation, Levels and Effects, United Nations Scientific Committee on the Effects of Atomic Radiation (UNSCEAR) 1972 Report: Report to the General Assembly, with Scientific Annexes. United Nations; 1972 Aug. doi:10.18356/5513731d-en

19. Heylmann D, Ponath V, Kindler T, Kaina B. Comparison of DNA repair and radiosensitivity of different blood cell populations. Sci Rep. 2021;11: 2478. doi:10.1038/s41598-021-81058-1

20. Qu J, Cheng T, Shi C, Lin Y, Ran X. A Study on the Activity of Fibroblast Cells in Connection with Tissue Recovery in the Wounds of Skin Injury after Whole-body Irradiation. Journal of Radiation Research. 2004;45: 341–344. doi:10.1269/jrr.45.341

21. Rudolph R, Vande Berg J, Schneider JA, Fisher JC, Poolman WL. Slowed growth of cultured fibroblasts from human radiation wounds. Plast Reconstr Surg. 1988;82: 669–677. doi:10.1097/00006534-198810000-00019

22. Hill A, Hanson M, Bogle MA, Duvic M. Severe Radiation Dermatitis is Related to Staphylococcus Aureus. American Journal of Clinical Oncology. 2004;27: 361. doi:10.1097/01.COC.0000071418.12121.C2

23. Elliott TB, Brook I, Stiefel SM. Quantitative study of wound infection in irradiated mice. Int J Radiat Biol. 1990;58: 341–350. doi:10.1080/09553009014551671

24. Landauer MR, Elliott TB, King GL, Bouhaouala SS, Wilhelmsen CL, Ferrell JL, et al. Performance Decrement after Combined Exposure to Ionizing Radiation and Shigella sonnei. Military Medicine. 2001;166: 71–73. doi:10.1093/milmed/166.suppl_2.71

25. Ran X, Cheng T, Lin Y, Qu J, Liu D, Al G, et al. Dose-effect relationships in total body irradiation on the healing of cutaneous wounds. Chinese Medical Journal. 2003;116: 878–882. doi:10.3760/cma.j.issn.0366-6999.2003.06.118

26. Carter SR, Chen MM, Palmer JL, Wang L, Ramirez L, Plackett TP, et al. Neutrophil Accumulation in the Small Intestine Contributes to Local Tissue Destruction Following Combined Radiation and Burn Injury. Journal of Burn Care & Research. 2016;37: 97–105. doi:10.1097/BCR.0000000000000220

27. Ledney GD, Madonna GS, Elliott TB, Moore MM, Jackson W 3rd. Therapy of infections in mice irradiated in mixed neutron/photon fields and inflicted with wound trauma: a review of current work. Radiat Res. 1991;128: S18-28.

28. Cooper RL, Segal RA, Diegelmann RF, Reynolds AM. Modeling the effects of systemic mediators on the inflammatory phase of wound healing. Journal of Theoretical Biology. 2015;367: 86–99. doi:10.1016/j.jtbi.2014.11.008

29. Murray JD. Mathematical Biology: I. An Introduction. Springer Science & Business Media; 2007.

30. Edelstein-Keshet L. Mathematical Models in Biology. Society for Industrial and Applied Mathematics; 2005.

31. Murphy K, Weaver W. Janeway's Immunobiology. Garland Science; 2017.
