# Supplementary figures and images for: Mathematical modeling of the combined effects of thermal burn and local irradiation
Source: PLoS One. 2026 Feb 10;21(2):e0341595. doi: 10.1371/journal.pone.0341595 (PMC12890176; doi:10.1371/journal.pone.0341595)

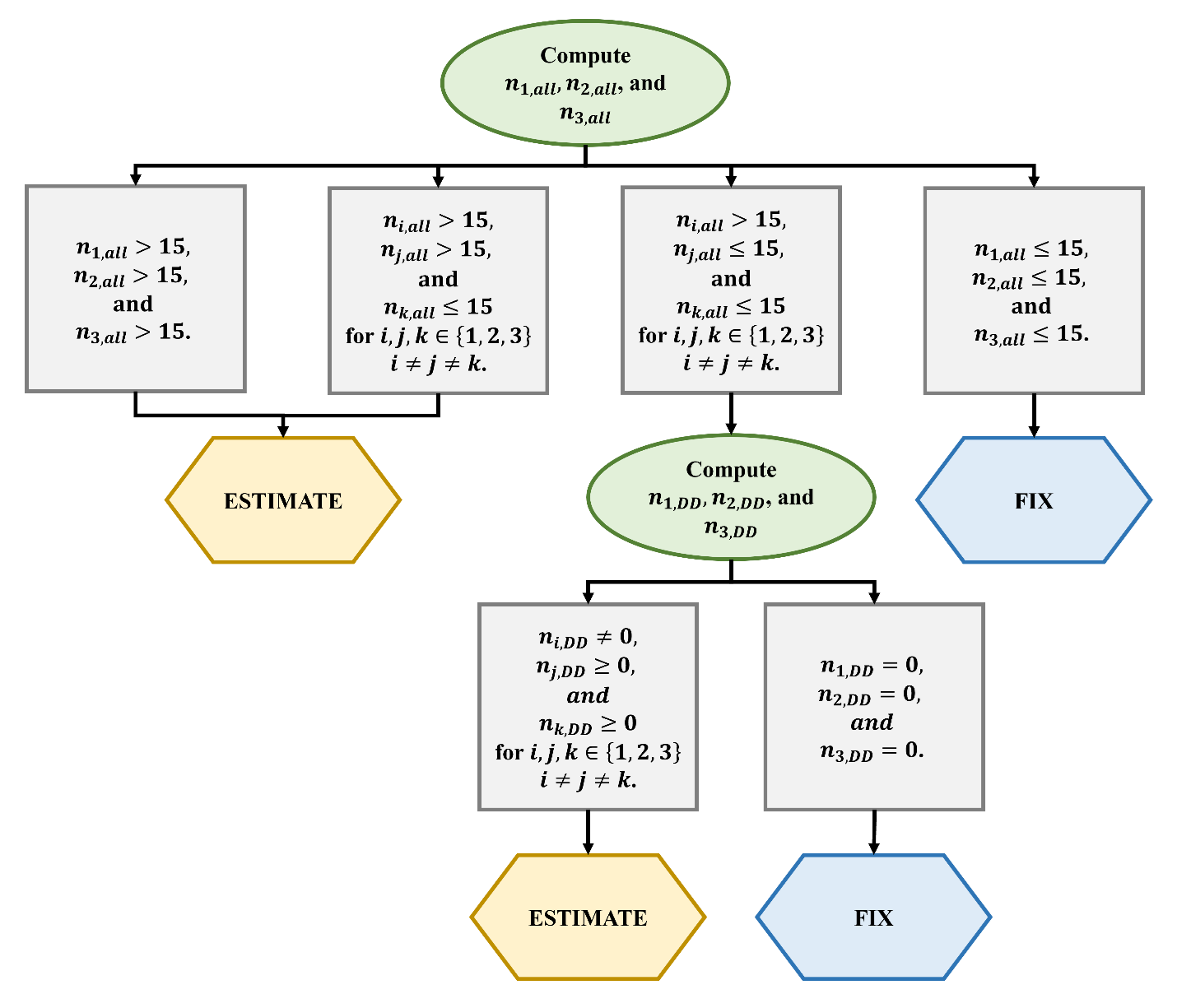

Supplement: S3 Fig — (TIF) [file pone.0341595.s003.tif]
