# Supplementary material for: Mathematical modeling of the combined effects of thermal burn and local irradiation
Source: PLoS One. 2026 Feb 10;21(2):e0341595. doi: 10.1371/journal.pone.0341595 (PMC12890176; doi:10.1371/journal.pone.0341595)
Supplement: S4 File — (PDF) [file pone.0341595.s004.pdf]

# S4 File. Parameter sampling and acceptance criteria.

Influential parameters were sampled on a uniform distribution over the same range used for sensitivity analysis for a total of 400,000 samples. Some parameter values were restricted based on assumed dynamics and steady state calculations. DNA damage caused by ionizing radiation initiates early cell death [1], thus we assume that damaged cells in the surrounding tissue have higher decay rates relative to undamaged cells. Some additional criteria were set to ensure the existence and stability of the healed steady state. The healed steady state exists where all variables in the thermal burn compartment are zero except fibroblasts which have a small positive value. The value of fibroblasts at steady state is calculated by the parameter values and includes $d_f - k_f$ in the denominator. Thus, we sample such that $k_f < d_f$ to ensure that this value exists. Due to the complexity of this model, it is not possible to analytically assess stability of the healed state, but we were able to calculate an inequality specific to the pathogen growth. We ensure that $k_{pg} < \frac{k_{pb}s_b}{\mu_b}$ otherwise pathogen does not resolve. This inequality has been used across other mathematical models involving foreign pathogen [2,3]. Additional acceptance criteria are shown in Table 1.

**Table 1. Acceptability criteria for parameter combinations prescribed under Latin hypercube sampling.**

| CRITERIA | BIOLOGICAL DESCRIPTION | MATHEMATICAL REPRESENTATION | REFS. |
|---|---|---|---|
| AC1 | Debris must be removed before damaged tissue can be repaired (this is a natural result of the progression of the stages of wound healing) | • $\tau_{deb} < \tau_{dam}$ | [4,5] |
| AC2 | In the absence of radiation, the irradiated thermal burn model collapses to the thermal burn model, satisfying previous estimates for healing time | • For a superficial thickness burn ($Dam_{tb}(0) = Deb_{tb}(0) = 0.1$),<br>▪ $\tau_{dam} = 4.667 \pm 0.5$ days<br>▪ $\left\|\boldsymbol{X}_{0.1}^{tail} - \boldsymbol{X}^*\right\|_2 < 0.3$ **<br>• For a minor superficial partial-thickness burn ($Dam_{tb}(0) = Deb_{tb}(0) = 0.9$),<br>▪ $\tau_{dam} = 7.833 \pm 0.5$ days<br>▪ $\left\|\boldsymbol{X}_{0.9}^{tail} - \boldsymbol{X}^*\right\|_2 < 0.3$ ** | [6–9] |
| AC3 | In the absence of radiation, superficial burns heal without scarring | • $\left\|\boldsymbol{X}_{0.1}^{tail} - \boldsymbol{X}^*\right\|_2 < 0.3$<br>• $\left\|\boldsymbol{X}_{0.9}^{tail} - \boldsymbol{X}^*\right\|_2 < 0.3$ | [10] |
| AC4 | Radiation delays wound healing in a dose-dependent manner | • $\tau_{0Gy} < \tau_{1Gy} < \tau_{7Gy} < \tau_{14Gy}$ | [11–14] |

| CRITERIA | BIOLOGICAL DESCRIPTION | MATHEMATICAL REPRESENTATION | REFS. |
|---|---|---|---|
| AC5 | Damage to the surrounding tissue delays immune cell infiltration to the wound site | • $\tau_{0Gy}^{Nmax} < \tau_{1Gy}^{Nmax} < \tau_{7Gy}^{Nmax} < \tau_{14Gy}^{Nmax}$<br>• $\tau_{0Gy}^{Mmax} < \tau_{1Gy}^{Mmax} < \tau_{7Gy}^{Mmax} < \tau_{14Gy}^{Mmax}$<br>• $\tau_{0Gy}^{Lmax} < \tau_{1Gy}^{Lmax} < \tau_{7Gy}^{Lmax} < \tau_{14Gy}^{Lmax}$<br>• $\tau_{0Gy}^{Fmax} < \tau_{1Gy}^{Fmax} < \tau_{7Gy}^{Fmax} < \tau_{14Gy}^{Fmax}$ | [12,15] |
| AC6 | Radiation induces macrophage polarization to the classically-activated ($M1_{tb}$) phenotype | • $\tau_{0Gy}^{M2} < \tau_{1Gy}^{M2} < \tau_{7Gy}^{M2} < \tau_{14Gy}^{M2}$ | [16] |

Notation: Let $\tau_{dam}$ and $\tau_{deb}$ be the time points such that $Dam(\tau_{dam}) < 0.01$ and $Deb(\tau_{deb}) < 0.01$, respectively. Let $\tau_{0Gy}$, $\tau_{1Gy}$, $\tau_{7Gy}$, and $\tau_{14Gy}$ be the time points such that $Dam(\tau_{0Gy}) < 0.01$, $Dam(\tau_{1Gy}) < 0.01$, $Dam(\tau_{7Gy}) < 0.01$, and $Dam(\tau_{14Gy}) < 0.01$ for a thermal burn irradiated with 0 Gy, 1 Gy, 7 Gy, or 14 Gy, respectively.

Let $\tau_{iGy}^{Nmax}, \tau_{iGy}^{Mmax}, \tau_{iGy}^{Lmax}, \tau_{iGy}^{Fmax}$ for $i = 0, 1, 7, 14$ be the time points such that $N_{tb}$, $M1_{tb} + M2_{tb}$, $L1_{tb} + L2_{tb}$, and $F_{tb}$, respectively, reach their maximum values.

Let $\tau_{iGy}^{M2}$ for $i = 0, 1, 7, 14$ be the time points such that $\frac{M2_{tb}}{M1_{tb}} > 1$, thus the time points at which the concentration of alternatively-activated macrophages ($M2_{tb}$) is larger than the concentration of classically-activated macrophages ($M1_{tb}$).

** $\|\cdot\|_2$ represents the 2-norm of a matrix, which computes the maximum absolute row sum. $\boldsymbol{X}_{0.1}^{tail}$ and $\boldsymbol{X}_{0.9}^{tail}$ represent the "tail" numerical solution over $t \in [0, 100 * 24]$ (in hours) of all state variables under superficial thickness burn ($Dam_{tb}(0) = Deb_{tb}(0) = 0.1$) and superficial partial-thickness burn ($Dam_{tb}(0) = Deb_{tb}(0) = 0.1$), respectively. The healed steady-state is that in which all state variables within the thermal burn, expect fibroblasts, are zero, i.e., $(Dam_{tb}^*, Deb_{tb}^*, N_{tb}^*, M1_{tb}^*, M2_{tb}^*, L1_{tb}^*, L2_{tb}^*, F_{tb}^*, P_{tb}^*)^T = \left(0,0,0,0,0,0,0, \frac{k_{ftb}^{ud}}{d_f - k_f} \cdot \frac{s_f}{k_{ftb}^{ud} + d_{fr}}, 0\right)^T$ where $T$ denotes the transpose of a matrix. Thus, $\boldsymbol{X}^*$ is taken to be $(Dam_{tb}^*, Deb_{tb}^*, N_{tb}^*, M1_{tb}^*, M2_{tb}^*, L1_{tb}^*, L2_{tb}^*, F_{tb}^*, P_{tb}^*)^T$, whence $\|\boldsymbol{X}_{0.1}^{tail} - \boldsymbol{X}^*\|_2$ and $\|\boldsymbol{X}_{0.9}^{tail} - \boldsymbol{X}^*\|_2$.

# References


1. Norbury CJ, Zhivotovsky B. DNA damage-induced apoptosis. Oncogene. 2004;23: 2797–2808. doi:10.1038/sj.onc.1207532

2. Reynolds A, Rubin J, Clermont G, Day J, Vodovotz Y, Bard Ermentrout G. A reduced mathematical model of the acute inflammatory response: I. Derivation of model and analysis of anti-inflammation. Journal of Theoretical Biology. 2006;242: 220–236. doi:10.1016/j.jtbi.2006.02.016

3. Cooper RL, Segal RA, Diegelmann RF, Reynolds AM. Modeling the effects of systemic mediators on the inflammatory phase of wound healing. Journal of Theoretical Biology. 2015;367: 86–99. doi:10.1016/j.jtbi.2014.11.008

4. Schultz GS, Chin GA, Moldawer L, Diegelmann RF. Principles of Wound Healing. Mechanisms of Vascular Disease: A Reference Book for Vascular Specialists. University of Adelaide Press; 2011. Available: https://www.ncbi.nlm.nih.gov/books/NBK534261/

5. Goldberg SR, Diegelmann RF. Wound Healing Primer. Surgical Clinics of North America. 2010;90: 1133–1146. doi:10.1016/j.suc.2010.08.003

6. Abazari M, Ghaffari A, Rashidzadeh H, Badeleh SM, Maleki Y. A systematic review on classification, identification, and healing process of burn wound healing. International Journal of Lower Extremity Wounds. 2020; 1–13.

7. Evers LH, Bhavsar D, Mailänder P. The biology of burn injury. Experimental Dermatology. 2010;19: 777–783. doi:10.1111/j.1600-0625.2010.01105.x

8. Jeschke MG, van Baar ME, Choudhry MA, Chung KK, Gibran NS, Logsetty S. Burn injury. Nat Rev Dis Primers. 2020;6: 11. doi:10.1038/s41572-020-0145-5

9. Peng D, Huang W, Ai S, Wang S. Clinical significance of leukocyte infiltrative response in deep wound of patients with major burns. Burns. 2006;32: 946–950. doi:10.1016/j.burns.2006.03.003

10. Finnerty CC, Jeschke MG, Branski LK, Barret JP, Dziewulski P, Herndon DN. Hypertrophic scarring: the greatest unmet challenge following burn injury. Lancet. 2016;388: 1427–1436. doi:10.1016/S0140-6736(16)31406-4

11. Chen Y, Zhang Q, Wu Y, Branch-Brooks CD, Butler CE. Short-term influences of radiation on musculofascial healing in a laparotomy rat model. Sci Rep. 2019;9: 11896. doi:10.1038/s41598-019-48201-5

12. Iddins CJ, DiCarlo AL, Ervin MD, Herrera-Reyes E, Goans RE. Cutaneous and Local Radiation Injuries. J Radiol Prot. 2022;42: 10.1088/1361-6498/ac241a. doi:10.1088/1361-6498/ac241a

13. Liu X, Liu J-Z, Zhang E, Li P, Zhou P, Cheng T-M, et al. Impaired Wound Healing after Local Soft X-Ray Irradiation in Rat Skin: Time Course Study of Pathology, Proliferation, Cell Cycle, and Apoptosis. Journal of Trauma and Acute Care Surgery. 2005;59: 682. doi:10.1097/01.ta.0000177674.55388.40

14. United Nations Scientific Committee on the Effects of Atomic Radiation. Ionizing Radiation, Sources and Biological Effects, United Nations Scientific Committee on the Effects of Atomic Radiation (UNSCEAR) 1982 Report: Report to the General Assembly, with Scientific Annexes. United Nations; 1982 Dec. doi:10.18356/34127272-en

15. Vegesna V, Withers RH, Holly EF, McBride WH. The Effect of Local and Systemic Irradiation on Impairment of Wound Healing in Mice. Radiation Research. 1993;135: 431–433.

16. Rödel F, Frey B, Multhoff G, Gaipl U. Contribution of the immune system to bystander and non-targeted effects of ionizing radiation. Cancer Letters. 2015;356: 105–113. doi:10.1016/j.canlet.2013.09.015
