# Supplementary material for: Mathematical modeling of the combined effects of thermal burn and local irradiation
Source: PLoS One. 2026 Feb 10;21(2):e0341595. doi: 10.1371/journal.pone.0341595 (PMC12890176; doi:10.1371/journal.pone.0341595)
Supplement: S5 File — (PDF) [file pone.0341595.s005.pdf]

S5 File. Mapping thermal fluence to damage and debris.

The burn injuries considered in this model are those which result from the direct absorption of radiant energy, referred to as flash burns [1]. Severity of flash burn injury is determined by the wavelength spectrum of thermal energy at the location of an exposed individual as well as the duration of their exposure [2]. Our mapping from thermal fluence to initial damage and debris levels at the burn site therefore incorporates effects that are distinctive to thermal radiation (i.e., the production of a thermal spectrum and pulse) and their impact on human skin through the deposition of energy and its subsequent biological processes.

To incorporate the effects of thermal radiation, we identified *a priori* a spectral profile to be used in conjunction with thermal fluence to determine severity of an individual's flash burn injury. This spectral profile was created using a DOD-controlled software (validated with data from Glasstone & Dolan (1977) [1]) which calculates thermal pulses and spectral data from a nuclear incident when provided with characteristics of the explosion and environment such as radiation yield, height of burst, and slant range. The profile chosen for our mapping, shown in Figure 1, is considered to be representative of the thermal spectrum expected to result in superficial and partial-superficial thickness flash burns.

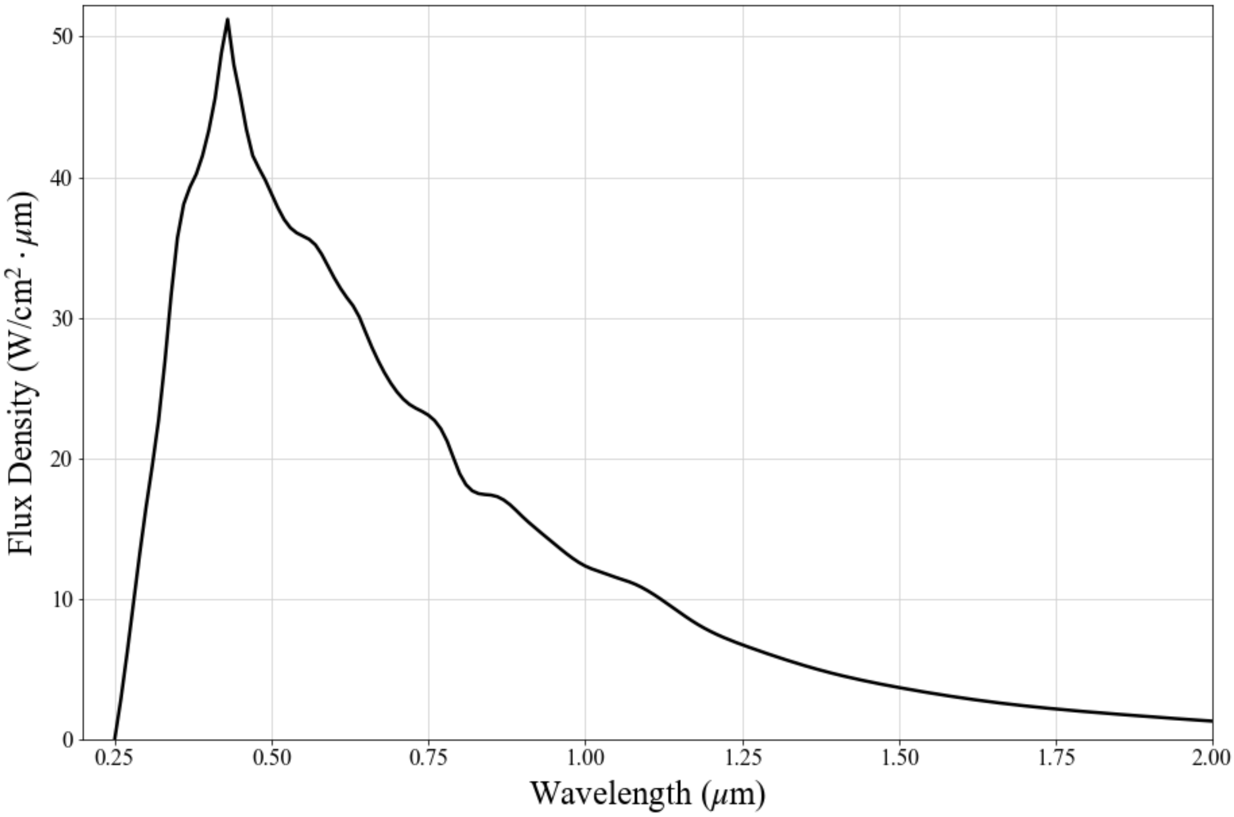

Figure 1. Shape of the spectral profile used as a representative thermal spectrum expected to result in superficial and partial superficial thickness thermal burns. This spectral profile is used in conjunction with thermal fluence to compute the thermal radiant energy deposited into each layer of skin tissue.

It has been observed that different wavelengths of light are reflected, scattered, and absorbed differently by the three primary layers of the skin (the epidermis, dermis, and subcutaneous tissue), and that the penetration depth of light generally increases with wavelength [3–7]. To construct the energy deposition profile, the penetration depth of each wavelength band can be used to convert the incident energy in various wavelength bands to an energy deposited into each tissue layer. Note that there are two steps required to complete this conversion: first, it is necessary to compute the incident energy in each band from the given thermal spectrum and fluence; and second, the penetration depth of the various wavelengths must be characterized in a more quantitative manner.

The incident energy in each band is obtained by integrating the spectrum output (as represented by Figure 1) over the different wavelength bands and subsequently computing the fractional power in each band. The fractional power in each band is then used in conjunction with wavelength penetration depth to determine energy deposition in the various tissue layers.

To quantitatively characterize penetration depth of the various wavelengths, data from Anderson and Parrish [3] that approximates penetration depth into fair-colored skin to a value of $1/e$ (37%) of the incident energy was used to fit parameters to the following logistic growth function that maps wavelength of light (W) to the depth of penetration into human skin (D_P):

$$D_P(W) = \frac{2.25657 \times 10^3}{1 + e^{\left(-6.69087 \times 10^{-3}(W - 8.05556 \times 10^2)\right)}}, \tag{1}$$

Figure 2 shows the logistic growth function given by Equation 1 plotted against the data obtained from Anderson and Parrish [3].

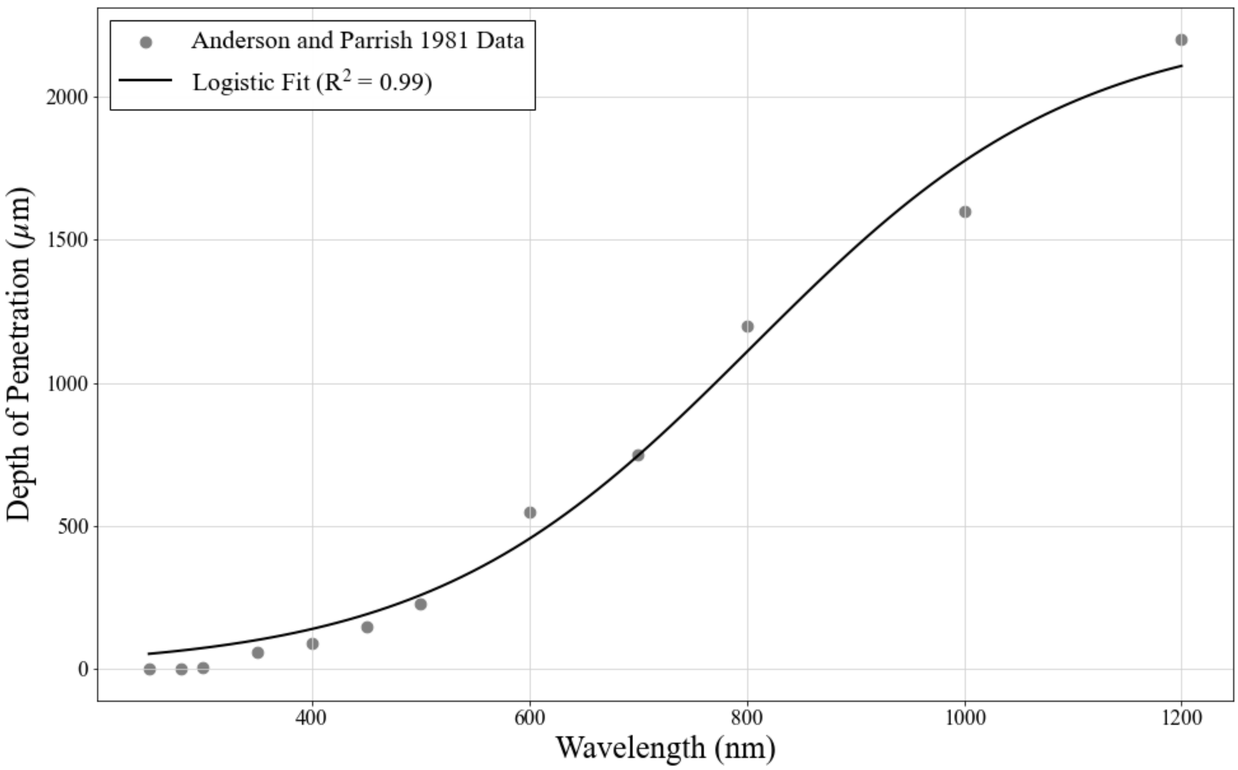

Figure 2. Curve fit of the wavelength-dependent penetration of light into human skin. Note that the R^2 value for the fitted logistic curve is 0.99, indicating a good fit to the Anderson and Parrish (1981) **[3]** data. The penetration depth predicted by this curve is used to convert incident radiant energy in each wavelength band to energy deposited in each tissue layer.

Thus, incident energy in each band can be converted to an energy deposited in each tissue layer, with penetration depths specified as *e*-folding lengths. This calculation is performed with a suitably normalized exponential function over the range of depths, which can be done analytically and does not require numerical integration. Finally, the energy deposited per unit volume (measured in units of J/cm^3), was calculated for each skin layer and each wavelength band using layer thickness data.

Note that skin thickness is highly dependent on body location and individual variability [8,9]. As such, a single position on the body was chosen for the examination of light penetration. The skin of the cheek was chosen as the face is unlikely to be covered by fluence obstructing clothing. A review of the literature was performed to obtain the typical thicknesses of skin layers in the human cheek, summarized in Table 1.

Table 1. Topographic thickness of skin layers in the human cheek. Subcutaneous tissue values were computed based on the thickness of other layers of skin. These values for skin layer thickness are used to compute the energy deposited into each layer of skin tissue.

Skin Layer	Typical Thickness Range (µm)	Assumed Thickness (µm)	Assumed Depth of Bottom of Layer from Skin Surface (µm)	Reference

Stratum Corneum	10-15	10	10	[9]
Lower Epidermis	20-50	20	30	[8]
Papillary Dermis	300-650	300	330	[8]
Reticular Dermis	450-1000	450	780	[8]
Subcutaneous Tissue	>2000	2000	2780	-

Once the deposited energy in each skin layer has been identified, the resulting temperature increase (ΔT) in each layer can be calculated by Equation 2.

$$\Delta T = \frac{E_d}{\rho c_p}, \tag{2}$$

where E_d is the deposited energy, ρ is the tissue density, and c_p is the heat capacity.

It is worth noting that the speed at which energy is deposited into the skin depends on the duration of the thermal pulse. Shorter pulse durations will deliver total energy per unit area more rapidly than longer pulses, leaving less time for heat dissipation by conduction and blood transport, therefore resulting in higher local skin temperature [1,2]. For the model presented herein, we consider a relatively short thermal pulse, and therefore the effect of skin cooling due to blood flow during energy deposition is expected to be negligible. As such, we assume an instantaneous deposition of energy and the temperature increase in each layer of the skin was treated as an initial condition to a bioheat transfer equation, given by the following:

$$\rho C \frac{d}{dt}[u(x,t)] = k \frac{d^2}{dx^2}[u(x,t)], \tag{3}$$

$$u(x,0) = T_0(x), \tag{4}$$

$$\frac{d}{dx}[u(0,t)] = \frac{h_a}{k}\left(u_{air} - u(0,t)\right), \tag{5}$$

$$u(L,t) = u_{blood}, \tag{6}$$

where the effective thermal conductivity k, measured in W/(cmºC), is defined as

$$k = \frac{k_{epd}L_{epd} + k_{derm}L_{derm}}{k_{epd} + L_{derm}}, \tag{7}$$

and the effective heat capacity C, measured in J/(gºC), is defined as

$$C = \frac{C_{epd}L_{epd} + C_{derm}L_{derm}}{L_{epd} + L_{derm}}, \tag{8}$$

The parameters of the bioheat transfer equations, along with their descriptions, units, and values, are provided in Table 2.

Table 2. Symbols, definitions, units, and values for the bioheat transfer equation. The bioheat transfer equation is used to obtain the temperature of tissue as a function of time and depth beneath the skin surface.

Symbol	Description	Units	Value	Reference
k_{epd}	Epidermal thermal conductivity	W/(cmºC)	0.0021	[10]
k_{derm}	Dermal thermal conductivity	W/(cmºC)	0.0037	[10]
C_{epd}	Epidermal heat capacity	J/(gºC)	3.6	[10]
C_{derm}	Dermal heat capacity	J/(gºC)	3.4	[10]
ρ	Density	g/cm^3	1.2	[10]
L_{epd}	Epidermal depth	cm	0.003	[8]
L_{derm}	Dermal depth	cm	0.075	[8]
L_{SC}	Stratum corneum depth	cm	0.001	[9]
L_{LE}	Lower epidermis depth	cm	0.002	[8]
L_{PD}	Papillary dermis depth	cm	0.03	[8]
L_{RD}	Reticular dermis depth	cm	0.045	[8]
L_{ST}	Subcutaneous tissue depth	cm	0.1	[8]
h_a	Connective heat transfer coefficient of unforced air	W/cm^2	0.001	[10]
u_{blood}	Blood temperature	ºC	37	-
u_{air}	Air temperature	ºC	27	-
L	Inner boundary depth	cm	0.178	-
Δx	Spatial grid step size	cm	0.001194631	-
Δt	Temporal grid step size	s	0.001	-
t_{final}	Total simulation time	s	50	-

The solution to the bioheat transfer equation provides a measure for the temperature of tissue as a function of both time and depth beneath the skin surface. This temperature can then be used with the Arrhenius damage equation to determine the depth of tissue damage initially present at the wound site.

The Arrhenius injury model is derived from standard physical-chemical thermal dynamics to describe the evolution of thermal damage over time for a given temperature [11]. It has been used for years to assess tissue damage at the burn site [12,13] and continues to be used as a valid reference for assessing damage related to temperature [10,14–16]. The Arrhenius damage equation is given by:

$$\frac{\partial}{\partial t}[\Omega(x,t)] = Ae^{-\frac{\Delta E}{Ru(x,t)}} \tag{9}$$

where Ω is the damage integral predicted by the model given a tissue temperature $u(x, t)$ in Kelvin, and the parameters R, A, and ΔE are defined as in Table 3.

Table 3. Symbols, definitions, units, and values for parameters of the Arrhenius damage equation. The Arrhenius damage equation is used to assess damage at the burn site for a given tissue temperature.

Symbol	Description	Units	Value	Reference
R	Universal gas constant	J/mol°K	8.314472	[14]
A	Frequency factor	1/s	3.1×10^{98}	[14]
ΔE	Activation energy of protein denaturization reaction	J/mol	6.28×10^{5}	[14]

Figure 3 displays the maximum burn depth predicted by the Arrhenius damage equation, $\Omega \geq 1$, for thermal fluence values ranging from 1 J/cm^2 to 19 J/cm^2.

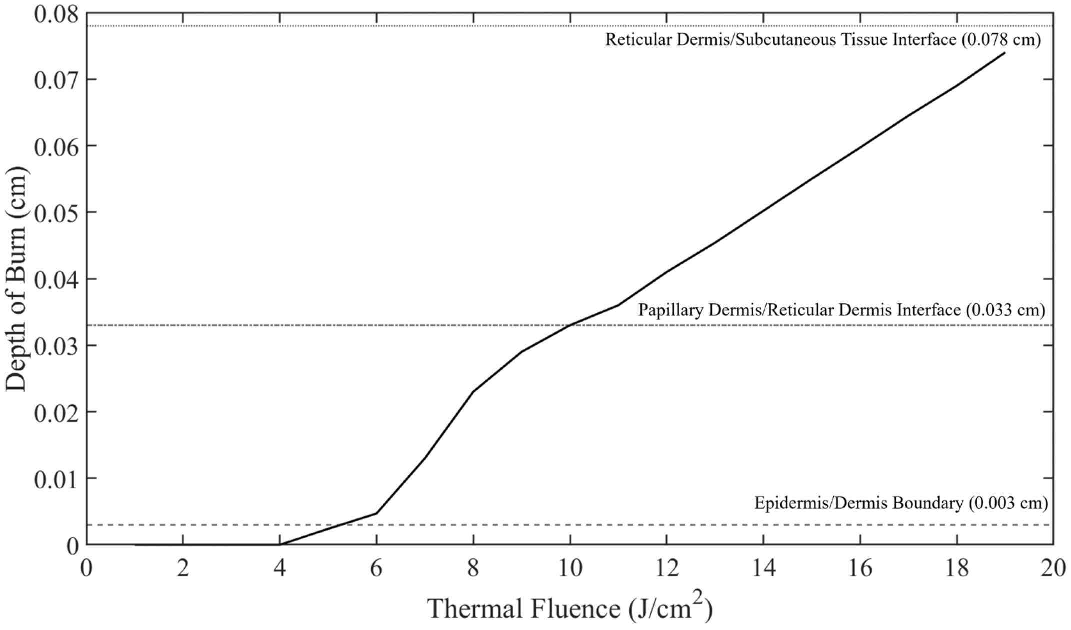

Figure 3. Plot of the Arrhenius injury model mapping thermal fluence (J/cm^2) to depth of burn (cm). Superficial burns extend into the epidermis (≤0.003cm) and superficial partial thickness burns extend into the dermis (≤0.078cm). More severe burns extending into the subcutaneous tissue are not considered in the present model.

The depth of severe damage predicted by the Arrhenius damage equation for thermal fluence values ranging from 1 J/cm^2 to 19 J/cm^2 was then used to fit a quadratic polynomial function, given by the following:

$$D(f) = -0.000089795f^2 + 0.0072019f - 0.031822, \tag{10}$$

where $D(f)$ is the predicted maximum depth of severe damage for a given thermal fluence $f > 5.016$ J/cm^2; for values of $f \leq 5.016$ J/cm^2, there is predicted to be no burn damage. Figure 4 compares burn depth predicted by the Arrhenius damage equation to burn depth predicted by Equation 10 for thermal fluence values ranging from 1 J/cm^2 to 19 J/cm^2.

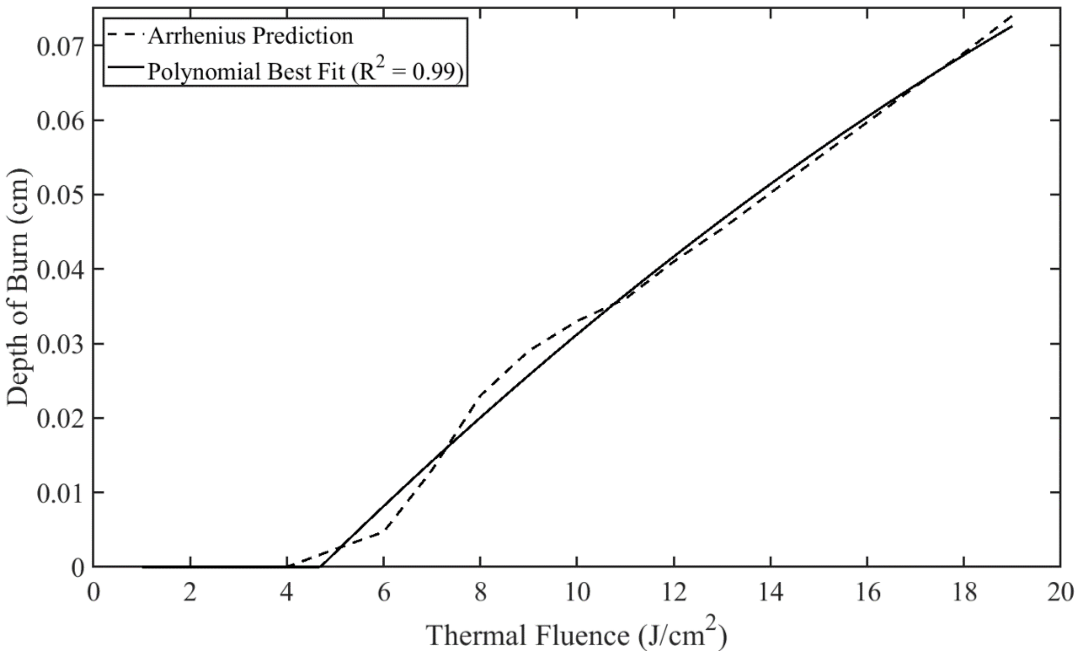

Figure 4. Comparison of burn depth predicted by the Arrhenius damage equation to burn depth predicted by the polynomial best fit function (Equation 10). Note that the R^2 value for the best fit function (Equation 10) is 0.99, indicating a good fit to predictions made by the Arrhenius damage equation. Equation 10 is used to compute the depth of severe burn predicted for a given thermal fluence, which can then be mapped to initial damage and debris levels for our model.

In our model, the initial condition $Dam_{tb}(0) = Deb_{tb}(0) = 0.1$ is associated with a superficial thermal burn and $Dam_{tb}(0) = Deb_{tb}(0) = 0.9$ with a superficial partial-thickness thermal burn. Due to the different cellular compositions of the epidermis and dermis, we developed a piecewise equation to model the level of tissue damage within the two layers. The depth marking the border between the epidermis and dermis was set to 30 μm (0.003 cm) based on data from facial skin tissue [1]. Equation 11 maps burn depth in centimeters to the damage and debris levels required for initialization of the model.

$$Dam_{tb}[0](D) = \begin{cases} \frac{100}{3}D & 0 \leq D \leq 0.003 \\ \frac{80}{3}D + 0.02 & 0.003 \leq D \end{cases} \tag{11}$$

By combining Equations 10 and 11, thermal fluence (in J/cm^2) can be directly mapped to initial conditions for the damage and debris variables of our TDPM, as shown in Equation 12.

$$Dam_{tb}[0](D) = \begin{cases} 0 & f < 5.016 \\ \frac{100}{3}(af^2 + bf + c) & 5.016 \leq f \leq 5.168 \\ \frac{80}{3}(af^2 + bf + c) + 0.02 & f > 5.168 \end{cases}, \quad (12)$$

where $a = -8.9795 \times 10^{-5}$, $b = 7.2019 \times 10^{-3}$, and $c = -3.1822 \times 10^{-2}$.

References

1. Glasstone S, Dolan PJ. The Effects of Nuclear Weapons. U.S. Department of Defense; 1977.

2. Reeves GI. Chapter 14: Effects on Personnel. EM-1: Capabilities of Nuclear Weapons. DTRA-EM-1-CH-14 (R1), Defense Threat Reduction Agency, Fort Belvoir, VA; 2015.

3. Anderson RR, Parrish JA. The Optics of Human Skin. Journal of Investigative Dermatology. 1981;77: 13–19. doi:10.1111/1523-1747.ep12479191

4. Ash C, Dubec M, Donne K, Bashford T. Effect of wavelength and beam width on penetration in light-tissue interaction using computational methods. Lasers Med Sci. 2017;32: 1909–1918. doi:10.1007/s10103-017-2317-4

5. Clement M, Daniel G, Trelles M. Optimising the design of a broad-band light source for the treatment of skin. Journal of Cosmetic and Laser Therapy. 2005;7: 177–189. doi:10.1080/14764170500344575

6. Gupta A, Avci P, Dai T, Huang Y-Y, Hamblin MR. Ultraviolet Radiation in Wound Care: Sterilization and Stimulation. Adv Wound Care (New Rochelle). 2013;2: 422–437. doi:10.1089/wound.2012.0366

7. Lim HW, Kohli I, Ruvolo E, Kolbe L, Hamzavi IH. Impact of visible light on skin health: The role of antioxidants and free radical quenchers in skin protection. J Am Acad Dermatol. 2022;86: S27–S37. doi:10.1016/j.jaad.2021.12.024

8. Chopra K, Calva D, Sosin M, Tadisina KK, Banda A, De La Cruz C, et al. A Comprehensive Examination of Topographic Thickness of Skin in the Human Face. Aesthetic Surgery Journal. 2015;35: 1007–1013. doi:10.1093/asj/sjv079

9. Böhling A, Bielfeldt S, Himmelmann A, Keskin M, Wilhelm K-P. Comparison of the stratum corneum thickness measured in vivo with confocal Raman spectroscopy and confocal reflectance microscopy. Skin Research and Technology. 2014;20: 50–57. doi:10.1111/srt.12082

10. Dai W, Wang H, Jordan PM, Mickens RE, Bejan A. A mathematical model for skin burn injury induced by radiation heating. International Journal of Heat and Mass Transfer. 2008;51: 5497–5510. doi:10.1016/j.ijheatmasstransfer.2008.01.006

11. Pearce JA. Models for thermal damage in tissues: processes and applications. Crit Rev Biomed Eng. 2010;38: 1–20. doi:10.1615/critrevbiomedeng.v38.i1.20

12. Cook MD, Haskins PJ. The development of a new Arrhenius-based burn model for both homogeneous and heterogeneous explosives. AIP Conference Proceedings. 1998;429: 337–340. doi:10.1063/1.55489

13. Orgill DP, Solari MG, Barlow MS, O'Connor NE. A Finite-Element Model Predicts Thermal Damage in Cutaneous Contact Burns. The Journal of Burn Care & Rehabilitation. 1998;19: 203–209. doi:10.1097/00004630-199805000-00003

14. Askarizadeh H, Ahmadikia H. Analytical study on the transient heating of a two-dimensional skin tissue using parabolic and hyperbolic bioheat transfer equations. Applied Mathematical Modelling. 2015;39: 3704–3720. doi:10.1016/j.apm.2014.12.003

15. Johnson LA. Laser diode burn-in and reliability testing. IEEE Communications Magazine. 2006;44: 4–7. doi:10.1109/MCOM.2006.1593543

16. Rylander MN, Feng Y, Zimmermann K, Diller KR. Measurement and mathematical modeling of thermally induced injury and heat shock protein expression kinetics in normal and cancerous prostate cells. International Journal of Hyperthermia. 2010;26: 748–764. doi:10.3109/02656736.2010.486778
