# Supplementary material for: Mathematical modeling of the combined effects of thermal burn and local irradiation
Source: PLoS One. 2026 Feb 10;21(2):e0341595. doi: 10.1371/journal.pone.0341595 (PMC12890176; doi:10.1371/journal.pone.0341595)
Supplement: S6 File — (PDF) [file pone.0341595.s006.pdf]

S6 File. Sensitivity analysis results.

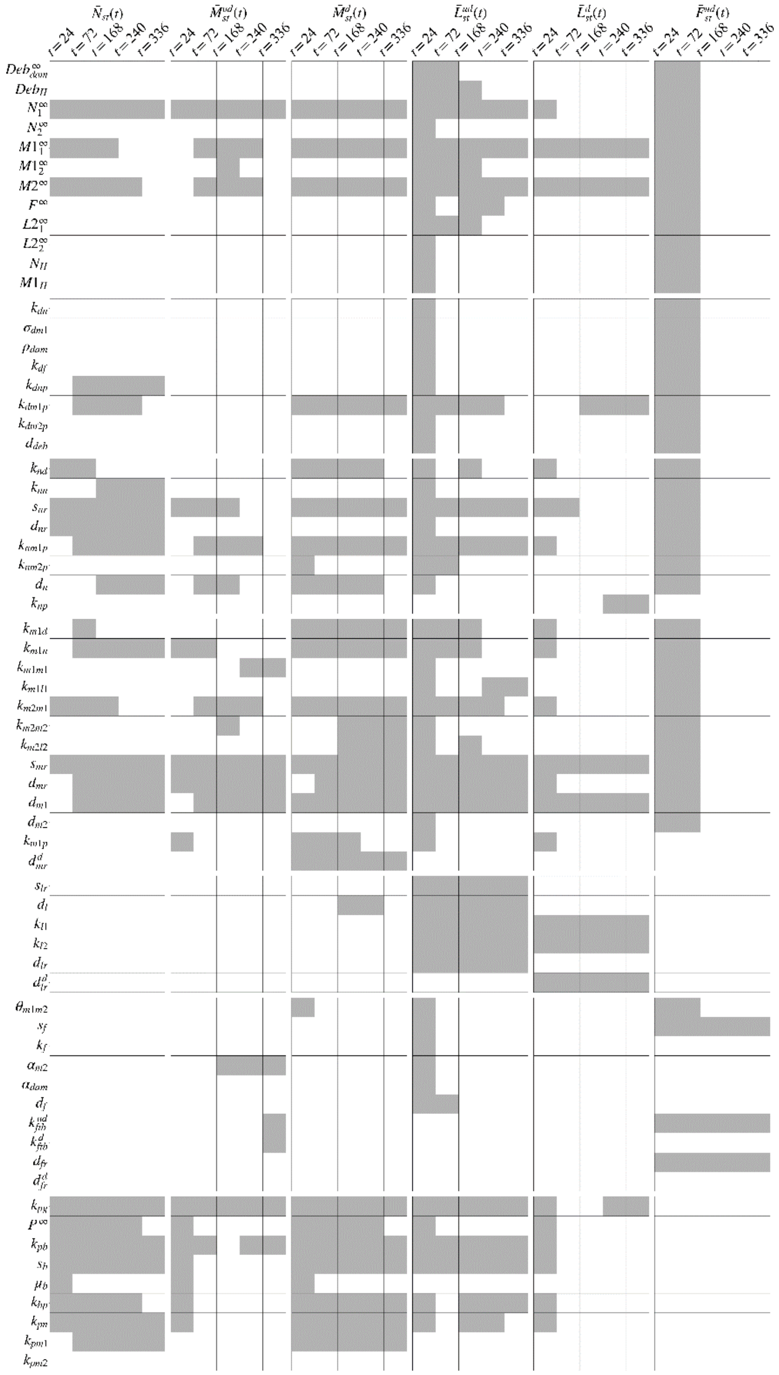

Figure 1. Sensitivity analysis results for the variables in the surrounding tissue compartment with a radiation dose of 1 Gy. Boxes are assigned to each parameter, variable, and time point. Boxes are shaded gray if the parameter was deemed influential for that variable at that time point. A parameter was deemed influential if the total sensitivity index was larger than the total sensitivity index assigned to the dummy parameter and the value was statistically significant (α=0.05) in at least two of the three prescribed statistical tests (t-test with Bonferroni correction, ANOVA with Tukey procedure, and Wilcoxon rank sum test).

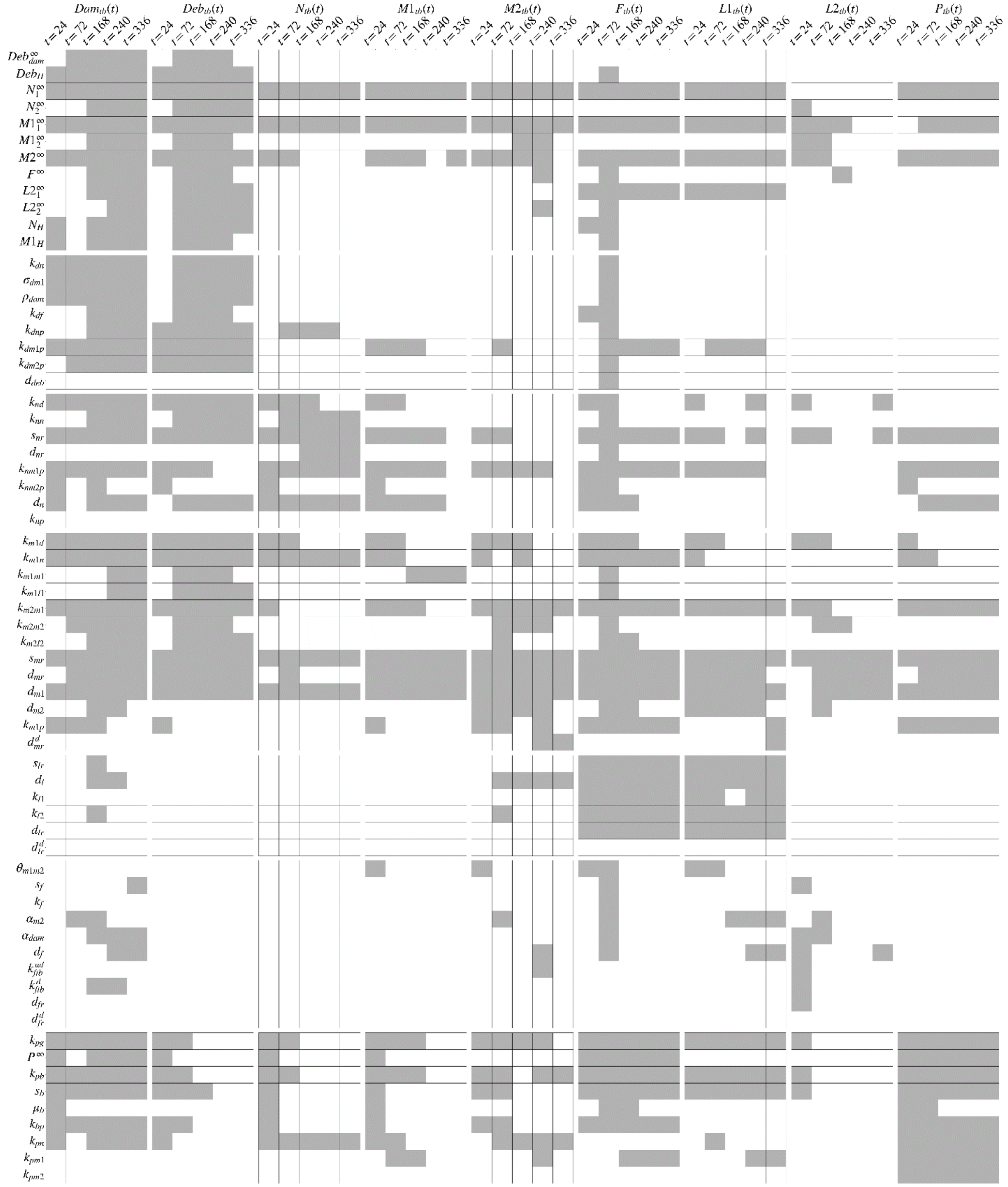

Figure 2. Sensitivity analysis results for the variables in the thermal burn compartment with a radiation dose of 1 Gy. Boxes are assigned to each parameter, variable, and time point. Boxes are shaded gray if the parameter was deemed influential for that variable at that time point. A parameter was deemed influential if the total sensitivity index was larger than the total sensitivity index assigned to the dummy parameter and the value was statistically significant (α=0.05) in at least two of the three prescribed statistical tests (t-test with Bonferroni correction, ANOVA with Tukey procedure, and Wilcoxon rank sum test).

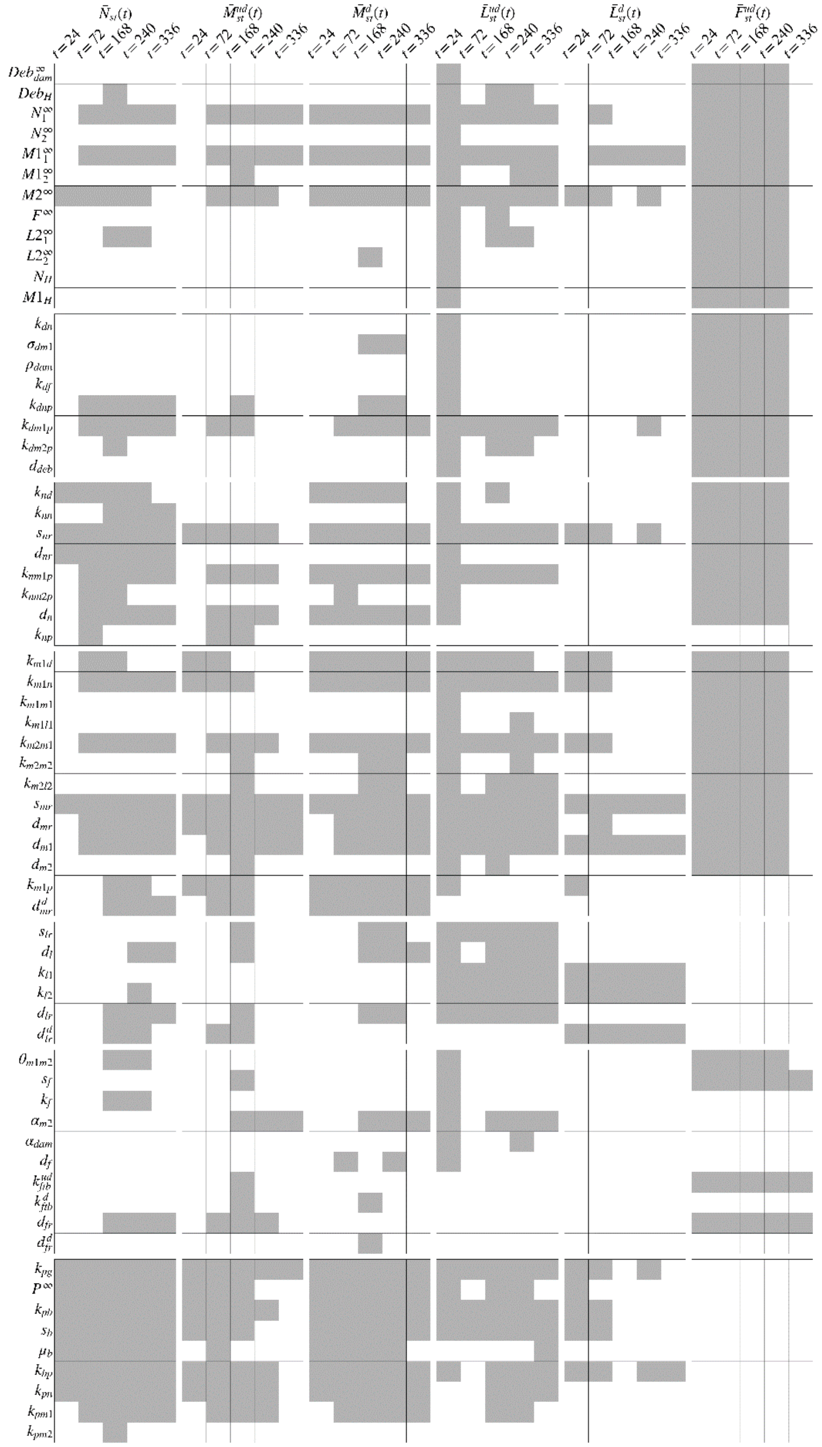

Figure 3. Sensitivity analysis results for the variables in the surrounding tissue compartment with a radiation dose of 7 Gy. Boxes are assigned to each parameter, variable, and time point. Boxes are shaded gray if the parameter was deemed influential for that variable at that time point. A parameter was deemed influential if the total sensitivity index was larger than the total sensitivity index assigned to the dummy parameter and the value was statistically significant (α=0.05) in at least two of the three prescribed statistical tests (t-test with Bonferroni correction, ANOVA with Tukey procedure, and Wilcoxon rank sum test).

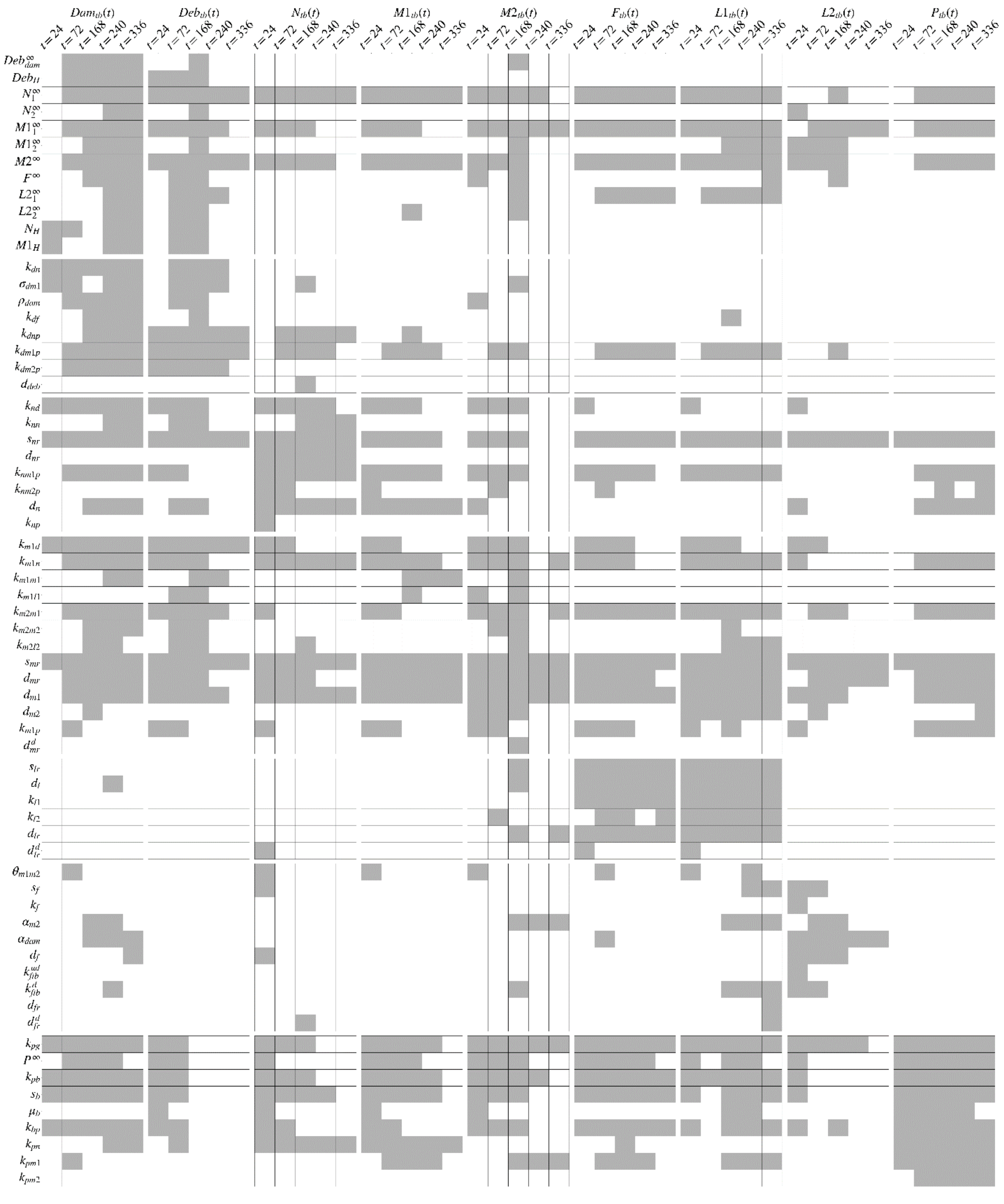

Figure 4. Sensitivity analysis results for the variables in the thermal burn compartment with a radiation dose of 7 Gy. Boxes are assigned to each parameter, variable, and time point. Boxes are shaded gray if the parameter was deemed influential for that variable at that time point. A parameter was deemed influential if the total sensitivity index was larger than the total sensitivity index assigned to the dummy parameter and the value was statistically significant (α=0.05) in at least two of the three prescribed statistical tests (t-test with Bonferroni correction, ANOVA with Tukey procedure, and Wilcoxon rank sum test).

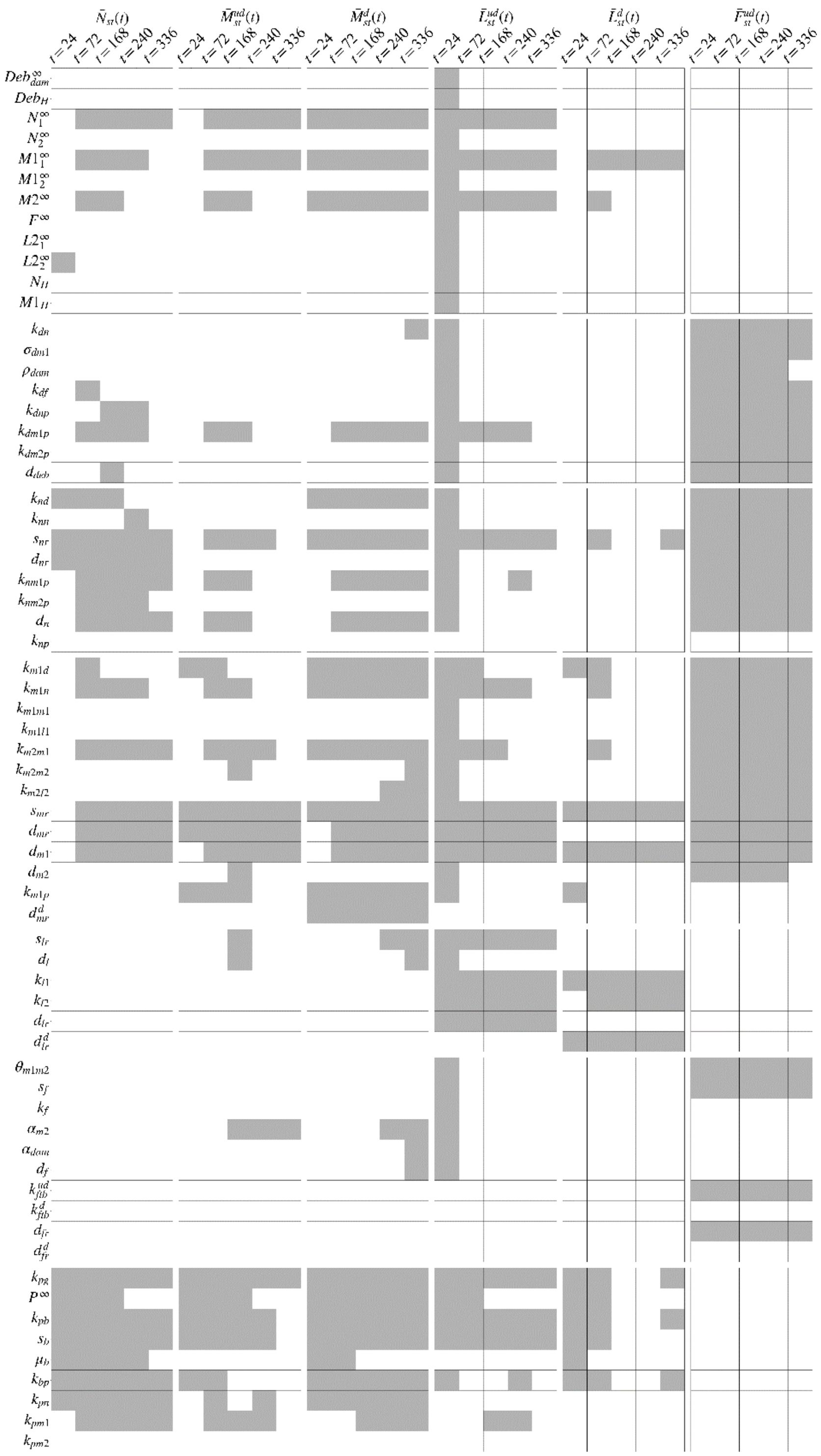

Figure 5. Sensitivity analysis results for the variables in the surrounding tissue compartment with a radiation dose of 14 Gy. Boxes are assigned to each parameter, variable, and time point. Boxes are shaded gray if the parameter was deemed influential for that variable at that time point. A parameter was deemed influential if the total sensitivity index was larger than the total sensitivity index assigned to the dummy parameter and the value was statistically significant (α=0.05) in at least two of the three prescribed statistical tests (t-test with Bonferroni correction, ANOVA with Tukey procedure, and Wilcoxon rank sum test).

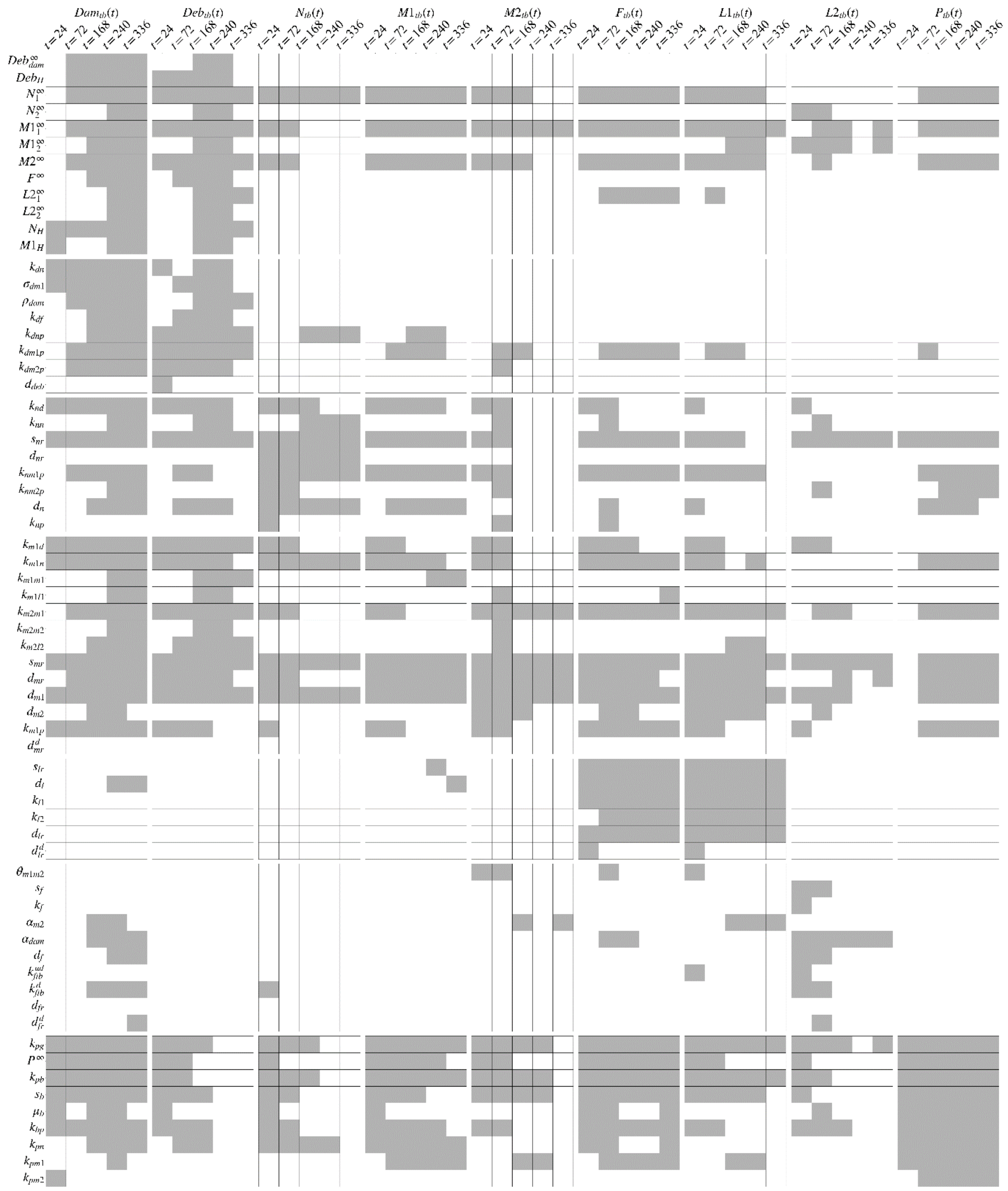

Figure 6. Sensitivity analysis results for the variables in the thermal burn compartment with a radiation dose of 14 Gy. Boxes are assigned to each parameter, variable, and time point. Boxes are shaded gray if the parameter was deemed influential for that variable at that time point. A parameter was deemed influential if the total sensitivity index was larger than the total sensitivity index assigned to the dummy parameter and the value was statistically significant (α=0.05) in at least two of the three prescribed statistical tests (t-test with Bonferroni correction, ANOVA with Tukey procedure, and Wilcoxon rank sum test).
